# Qualitative Analysis of Microbial Dynamics during Anaerobic Digestion of Microalgal Biomass in a UASB Reactor

**DOI:** 10.1155/2017/5291283

**Published:** 2017-11-13

**Authors:** Anna Doloman, Yousef Soboh, Andrew J. Walters, Ronald C. Sims, Charles D. Miller

**Affiliations:** ^1^Department of Biological Engineering, Utah State University, Old Main Hill 4105, Logan, UT 84322-4105, USA; ^2^Department of Food Processing, Palestine Technical Colleges, Arroub, P.O. Box 14, West Bank, State of Palestine

## Abstract

Anaerobic digestion (AD) is a microbiologically coordinated process with dynamic relationships between bacterial players. Current understanding of dynamic changes in the bacterial composition during the AD process is incomplete. The objective of this research was to assess changes in bacterial community composition that coordinates with anaerobic codigestion of microalgal biomass cultivated on municipal wastewater. An upflow anaerobic sludge blanket reactor was used to achieve high rates of microalgae decomposition and biogas production. Samples of the sludge were collected throughout AD and extracted DNA was subjected to next-generation sequencing using methanogen* mcrA* gene specific and universal bacterial primers. Analysis of the data revealed that samples taken at different stages of AD had varying bacterial composition. A group consisting of Bacteroidales, Pseudomonadales, and Enterobacteriales was identified to be putatively responsible for the hydrolysis of microalgal biomass. The methanogenesis phase was dominated by* Methanosarcina mazei*. Results of observed changes in the composition of microbial communities during AD can be used as a road map to stimulate key bacterial species identified at each phase of AD to increase yield of biogas and rate of substrate decomposition. This research demonstrates a successful exploitation of methane production from microalgae without any biomass pretreatment.

## 1. Introduction

Anaerobic digestion (AD), being a dynamically changing microbiological process, has long been manipulated only at the level of reactor design and physicochemical maintenance. Manipulation on the level of microorganisms in the system is more recent as evidenced by the rising number of studies investigating key bacterial players in AD [1–5]. Since AD consists of tightly linked biochemical stages that include hydrolysis, acetogenesis/acidogenesis, and methanogenesis, each of these stages is a possible aim for targeted manipulation of microbial consortia. A targeted manipulation at a certain stage of AD can remove a process bottleneck associated with rate-limiting hydrolysis, accumulation of volatile fatty acids that are toxic to the methanogenic bacteria, and even low amount of biogas production [6]. To facilitate targeted manipulation and monitor microbial diversity in working bioreactors, recent studies have highlighted the utilization of molecular techniques such as FISH (fluorescent in situ hybridization), DNA-hybridization on microchips, qPCR, and flow cytometry [7, 8]. Such management would be beneficial in order to predict possible failures in the AD due to shifts in the microbial communities and also to maintain proper organic loading rates of substrate and assess overall healthy condition of digesters.

The spectrum of substrates used for the AD has broadened greatly during the last five years, with utilization of a previously thought difficult to digest biomass, such as biomass with high cellulose content like grass and silage [9–13]. One substrate still resistant to AD is microalgal biomass. Microalgae, being widely present in eutrophicated lakes and wastewater lagoons, can serve as a biomass source for the production of biofuels. Microalgal biomass has been historically used for biodiesel production, due to its high lipid content [14–16], and only within the last 5–7 years have microalgae received an increased attention as a substrate for AD. Resistance of microalgal biomass to AD is mainly contributed by the presence of complex polysaccharides in the structure of microalgal cell walls, which makes the hydrolysis of this biomass a rate-limiting step in the biomethane production process. This limitation can be resolved with initial pretreatment of microalgal biomass by thermal, chemical, ultrasound, and ozonation processes and even application of constant magnetic field [17–26]. In addition to the difficulties with initial hydrolysis of microalgae, natural low carbon to nitrogen ratio of this substrate is not sufficient to sustain AD, and to overcome this limitation, a usual strategy is blending microalgal biomass with rich carbon sources prior to digestion, such as paper and maize silage [24, 27, 28]. Codigestion with conventional AD substrates, such as swine manure and waste activated sludge, is also popular, but in some cases yields of methane are decreased, yielding, however, higher total biogas yields [29, 30].

In our study, we investigated AD of intact microalgal biomass, harvested from wastewater lagoons (Logan Wastewater Lagoons, Logan, Utah). The Logan Lagoons municipal wastewater treatment plant utilizes a system of facultative lagoons in parallel and series arrangement with a total wastewater detention time of 60 to 90 days, occupies an area of 640 acres (2.56 km), and treats 10–15 MGD. Microalgal biomass grows at the surface of the water-air interface throughout the lagoon system. Harvested microalgal biomass for the experiment was mixed with sodium acetate to increase carbon to nitrogen ratio. Anaerobic digestion was performed in an upflow anaerobic sludge blanket reactor (UASB) (see Supplemental Figure 1 in Supplementary Material available online at https://doi.org/10.1155/2017/5291283). In the UASB process, influent is distributed throughout the system in upflow mode, bottom to up, flowing through a sludge blanket of anaerobic microorganisms. A constant contact between influent and microorganisms in a sludge bed results in a digestion of organic matter in the influent and production of a biogas. Generated biogas in a form of gas bubbles raises to the upper part of the reactor, where it is captured in a gas collection dome. A mixture of digested influent and sludge is kept from rising into the gas collection dome due to the separating baffles, installed around the circumference of the reactor. Liquid without sludge and heavy particles is allowed to pass into the effluent collection system, located above baffles.

In this study sludge bed microorganisms were analyzed over the course of time to assess microbial dynamics and to identify potential alga-lytic bacteria via analysis of a bacterial metagenome. Understanding how microorganisms coordinate AD of microalgal biomass will help to maintain biosystem stability during future AD and can be incorporated into the growing knowledge database on the microbiology of AD. This information can be further utilized to create an effective system to monitor AD with molecular techniques (FISH, qPCR, etc.) and to design effective microbial consortia that will increase biogas yields.

## 2. Materials and Methods

### 2.1. Reactor Design and Operation

Duplicates of UASB reactors were made of Plexiglass at the Utah Water Research Laboratory (UWRL) and each had a working volume of 32.4 L. Reactors had deflectors to prevent washout of sludge bed solids and three phase separators to direct collection of biogas (Supplemental Figure 1). There were three sample collection ports along the height of the reactor and a substrate distribution system 5 cm above the reactor bottom. Thermostat control of a rubber heating tape around reactor, thermocouple, and insulation enabled maintenance of a temperature regime at 35 ± 2°C. A peristaltic pump with a double channel head was used to feed both reactors. Generated biogas passed through the ice-cooling system to ensure moisture-free monitoring of biogas flow via flow meter with a working range of 0 to 500 sccm/min. The flow meters were calibrated using a mixture of methane and carbon dioxide of 80% and 20%, respectively, and were connected to a Campbell Scientific data logger type CR800 to measure millivolts of the output form the flow meters. The methane composition was measured every 5 to 6 days using a gas chromatograph (GC) with a thermal conductivity detector (TCD), a packed column (Alltec, CTR1) 1.83 m × 6.35 mm, and a Valco injection valve with a 500 *μ*L sample loop.

Each reactor was seeded with 11 L of anaerobic sediment from Logan Lagoons, Utah, which resulted in 9.7 g VSS (dry weight)/L of reactor volume. Sediments from Logan Lagoons were chosen as a reliable source of the anaerobic inoculum utilized in previous AD studies [32]. Reactors were fed with a mixture of microalgal biomass and sodium acetate to achieve a final C/N ratio of 21 : 1. Microalgal biomass was obtained by continuous centrifugation of the water from Logan Lagoons every 10–15 days. Microalgae comprised the genera such as* Scenedesmus*,* Chlorella*,* Chlorococcum*,* Chlamydomonas*,* Synedra*,* Navicula*,* Schroederia*, and* Euglena*,* Coelastrum* and some members of nonheterocystous cyanobacteria. The average COD of microalgal biomass was 72 g/L, with C/N ratio of 5/1. To increase the C/N ratio to the favorable value for anaerobic digestion of 21 : 1, sodium acetate was chosen as a rich, readily available carbon source. The feedstock had a final pH of 6-7 and pH fluctuations were adjusted with a hydrochloric acid solution. To acclimatize inoculum to the microalgae and sodium acetate in a feedstock, low organic loading rates (OLR) were initially applied, 0.9 gCOD/L·d, which were gradually increased during the operation of the reactor based on reactor performance and COD removal efficiency. Final OLR was 5.4 gCOD/L·d. Hydraulic retention time for the substrate was gradually decreased from 7 days to 5 days. Reactors were operated for 81 days.

### 2.2. Sampling, DNA Extraction, and Sequencing

Samples of the sludge bed microbial community were taken throughout the time course of anaerobic digestion (days 19, 57, and 75). Duplicate sludge bed samples were obtained from bottom and upper sampling ports of the UASB reactors and were stored at −80°C immediately after the collection. Extraction of DNA was performed using PowerSoil DNA isolation kit (MoBio, Carlsbad) following the manufacturer's instructions. Resulting DNA was used for the PCR amplification with* mcrA* gene specific primer set and universal bacterial 16S rDNA specific primer set (Supplemental Table 1) [33–35]. Each primer had a preceding adapter sequence (forward or reverse) specific for the Illumina MiSeq platform. PCR reactions were performed using KAPA HiFi HotStart ReadyMix (Kapa Biosystems, Wilmington) under the following conditions: initial denaturation at 95°C for 3 minutes, followed by 25 cycles consisting of 30 seconds at 95°C, 30 seconds at primer annealing temperature, and 30 seconds at 72°C. Final extension lasted 5 minutes at 72°C. Primer annealing temperature was 50°C for primer pair 338F and 785R and 56°C for ML primer pair. PCR products were submitted to the Molecular Research Core Facility at the Idaho State University (Pocatello, ID, USA) for further purification, library preparation (Nextera kit), and sequencing on the Illumina MiSeq platform (following manufacturer's instructions [36]).

### 2.3. Computational Analysis

Analysis of 16S rRNA gene data was performed using a MiSeq SOP pipeline, described by Kozich et al. [37] and implemented on MOTHUR software [38]. Analysis included (1) quality trimming of the reads, (2) chimera check with UCHIME algorithm, (3) extraction of unique reads and alignment to the classification databases, (4) actual classification using Bayesian classifier, and (5) OTU identification. Sequences generated from PCR with both types of primers, universal bacterial 338F and 785R and methanogen-specific MLr-MLf, were processed in a similar pipeline, with the only difference regarding database used for the sequences alignment and classification. For sequences generated with 338F and 785R primer set, SILVA V4 database (http://www.arb-silva.de/) was used for the classification and alignment. For sequences generated with* mcrA* gene specific primer set, a database for classification and alignment was manually created from pooling the* mcrA* sequences from FunGene database (http://fungene.cme.msu.edu/). The algorithm for analysis of* mcrA* sequences in MOTHUR software was previously described [39]. To build a phylogenetic tree of the classified* mcrA* sequences, MEGA 6.06 package was used, incorporating Tamura-Nei model with maximum likelihood analysis and 1000 bootstraps.

The internal MOTHUR command unifrac.weighted was used to calculate the significance of separate clustering of sequences from the samples taken at different time points of anaerobic digestion. A statistical tool in MOTHUR, HOMOVA, was used to calculate the level of variation among samples depending on the duration of anaerobic digestion. In more detail, algorithm assessed variability of OTU composition at different time points during AD, comparing level of variation for one pair of samples at a time (e.g., difference in variation of OTU composition between initial inoculum and samples taken at the end of AD). Beta-diversity for each sample amplified and sequenced with universal bacterial primer pair was estimated in a comparative heat map, while looking at the relative abundance of each OTU across all samples. Bacterial OTUs of interest were pulled from the classification table with custom Python scripts. Finally, depth of the conducted sequencing effort (rarefaction curve) was calculated using summary.single command with estimation of Good's coverage.

### 2.4. Data Accessibility

All metagenome sequences (both universal bacterial and* mcrA* gene specific) are accessible through the NCBI Sequence Read Archive (SRP058350).

## 3. Results

### 3.1. Anaerobic Digestion of Microalgal Biomass and Sodium Acetate

**Figure 1:**
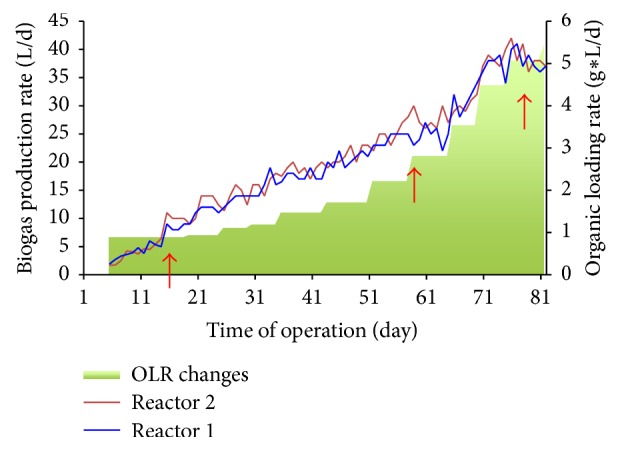
Biogas production rate and changes in the OLR during AD of microalgae and sodium acetate in two reactors. Arrows point to the days, when sludge samples were taken.

Results on utilization of a UASB reactor (Supplemental Figure 1) to digest a mixed feedstock of microalgae and sodium acetate are described in a recently published paper by two of this paper's authors [40] and this research is specifically aimed at results from analysis of microbial community that lead to the process of anaerobic digestion. Briefly, feedstock for the anaerobic digestion was combined with final C/N ratio of 21/1 and biogas production rate was 37 L/day during the last week of reactors operation (days 74–81, Figure 1). At organic loading rates corresponding to the initial COD of influent 6.25 g/L that was increased to 27.2 g/L, the UASB reactors demonstrated an average COD removal rate of 79% [40]. Utilization of microalgal biomass and sodium acetate as a feedstock for AD in UASB yielded, on average, 85% methane in the produced biogas [40]. The fraction of methane gas that was produced explicitly from microalgal biomass was calculated from the mass balance of influent COD conversion including production of cell mass [41]. Method and calculations are described in detail in the paper by Soboh et al. [40] and it demonstrates an estimation of 11–26% of methane being produced explicitly from decomposition of microalgal biomass. With the satisfactory performance of both reactors, samples of sludge bed were taken during the operation of AD (days 19, 57, and 75) and processed as described in Materials and Methods.

### 3.2. Sequencing of the DNA from Sludge Samples

**Figure 2:**
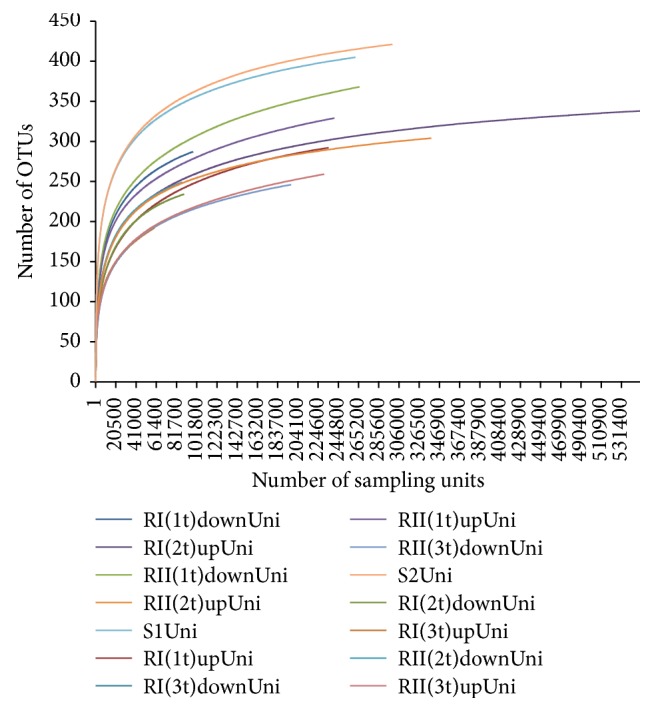
Rarefaction curve of the microbial diversity throughout the time course of anaerobic digestion of microalgae and sodium acetate.

A total of 7,433,629 reads were generated during the sequencing of all samples from the amplification of 16S rRNA and methanogen-specific* mcrA* genes. Sequencing of PCR product from amplification with 16S rRNA universal bacterial primer set resulted in 5,721,724 reads, while sequencing after amplification with primer set specific for the* mcrA* gene yielded 171,190 reads. In the 16S rRNA set, 975,677 reads were identified as unique. Rarefaction curve for the depth of the sequencing effort for 16S rRNA data is demonstrated in Figure 2. For the* mcrA* gene set, after quality trimming and chimera checking, 64.7% of new sequences were identified as unique (other reads were copies of those in a unique set) and used for further classification.

### 3.3. Classification of Identified OTUs in Bacterial 16S rRNA Samples

**Figure 3:**
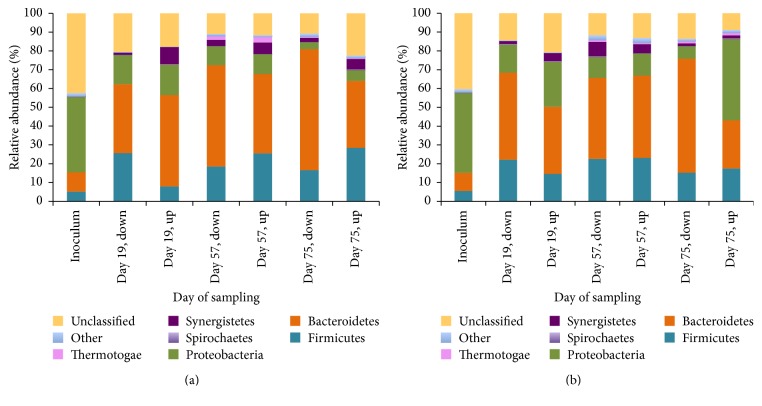
Microbial dynamics on phyla level in the UASB reactors (reactor 1 (a) and reactor 2 (b)) digesting microalgal biomass and sodium acetate. Phyla Armatimonadetes, Tenericutes, Actinobacteria, OD1, and Verrucomicrobia each contributed less than 1% of the total shared microbial population among all samples and were combined under the general “other” designation.

Amplification and sequencing with universal bacterial primers (338F and 785R) resulted in identification of 640 different bacterial OTUs. To understand dynamic changes in the microbial composition of a sludge bed during the AD of microalgal biomass and sodium acetate, it was necessary to identify key shared OTUs among all samples. A command get.sharedseqs in the MOTHUR package was used. Shared among all of the samples were 61 core taxa, and an additional 10 taxa groups were assigned as “unclassified” (Supplementary Table 2). The core 61 taxa were distributed among 11 major phyla, Firmicutes, Bacteroidetes, Proteobacteria, Spirochaetes, Synergistetes, Armatimonadetes, Tenericutes, Actinobacteria, OD1, Verrucomicrobia, and Thermotogae. Dynamics of microbial composition during the course of AD can be observed in Figure 3.

The Proteobacteria phylum had the biggest decrease in the number of assigned sequences in comparison with initial inoculum composition. In reactor 1 (Figure 3(a)), Proteobacteria-assigned sequences decreased from 48% in the initial inoculum to 23% on day 19; and in reactor 2 a decrease was from 51% to mean 26% across the sludge bed. The opposite was true for the sequences assigned to the Bacteroidetes phylum, where there was a defined increase from 11% (10% for the reactor 2) to the 42% (32% for the reactor 2) of the total classified sequences in 19 days of reactors operation on microalgal biomass and sodium acetate.

**Figure 4:**
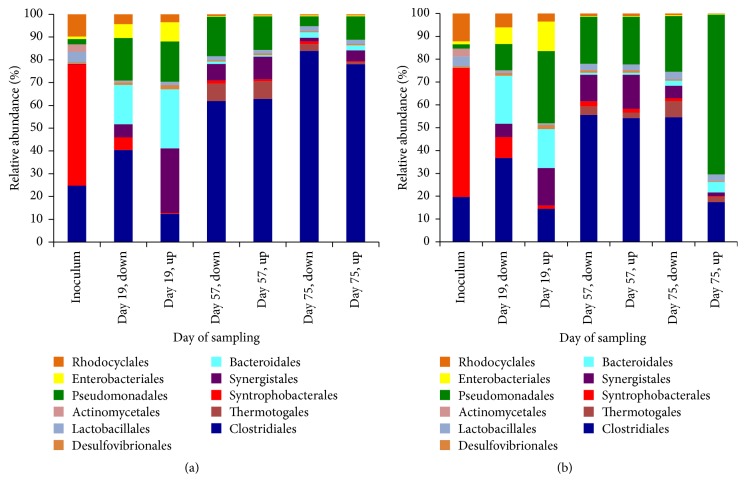
Microbial dynamics on order level for UASB reactor 1 (a) and reactor 2 (b), digesting microalgal biomass and sodium acetate.

To define major bacterial contributors in the microbial composition during digestion of microalgae and sodium acetate, core OTUs were classified on the order level (Figure 4). Both reactors demonstrate similar patterns of microbial dynamics during AD. This patterns include an increase in the number of sequences classified as Bacteroidales, Pseudomonadales, Enterobacteriales, and Synergistales during the start-up of reactors (the 19-day period) and a decrease in the number of sequences related to Syntrophobacterales, Rhodocyclales, Actinomycetales, and Lactobacillales during the same 19-day start-up period. The period after the start-up, sampling days 57 and 75, is characterized by a specific increase in the amount of Clostridiales in both reactors and an increase of Pseudomonadales in reactor 2. Percentage-wise, in reactor 1, Pseudomonadales reached the highest of 17% of the microbial population on day 19 (down and upper fractions combined), whereas in reactor 2, the highest population of Pseudomonadales was on day 75, 60%. For Clostridiales, a complete opposite pattern is observed: the highest population for reactor 1 was on day 75, when Clostridiales comprised 80.7% of the microbial population, while for reactor 2 number of Clostridiales sequences was not higher than 54.4% on day 57.

### 3.4. Comparative Qualitative and Statistical Analysis of Bacterial Population Profiles throughout the Course of AD

To assess the statistical relevance of changes in the bacterial group composition between samples of 16S rRNA taken at different time points of AD, unifrac.weighted command in MOTHUR was used. This command compares pairwise all the sampling groups and upper and down samples were combined. Results of assessment of separation significance are presented in Table 1. Since *W*Sig has a *p* value that should be <0.05 [42], results in Table 1 demonstrate a significant (*W*Sig < 0.001 and *W*Sig < 0.05) separation of OTU groups at different stages of AD.

An additional statistical assessment was conducted to ensure close relation of samples taken at the same time points of AD but from different reactors. This was necessary from the standpoint of replicating the experimental design in two reactors. From the heat map (Supplemental Figure 2), calculated with* jclass* algorithm in MOTHUR, one can see that beta-diversity (internal compositional heterogeneity) of samples taken at the same time point from two reactors is closely related to each other (bright red color, on a diagonal of the pyramid), whereas samples are significantly different in OTU composition when compared to samples taken at different time points (19th day and 57th day, e.g.).

### 3.5. Classification of Identified OTUs in Methanogen* mcrA* Gene Sequencing Data

**Figure 5:**
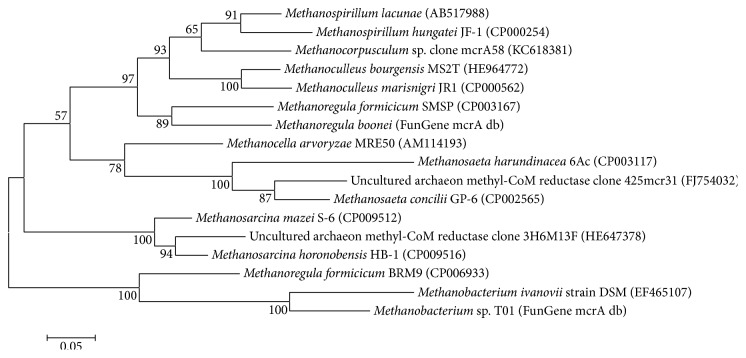
Phylogenetic tree of all identified methanogenic species in the amplified* mcrA* gene samples.

Reads generated from amplification with* mcrA* gene specific primer set were quality trimmed and analyzed in MOTHUR software package. Classification of aligned reads in a FunGene database resulted in the identification of 14 different species of methanogenic bacteria and 2 uncultured/unclassified archaeal species. A phylogenetic tree of all identified species (all time points of AD are combined) is depicted in Figure 5.

**Figure 6:**
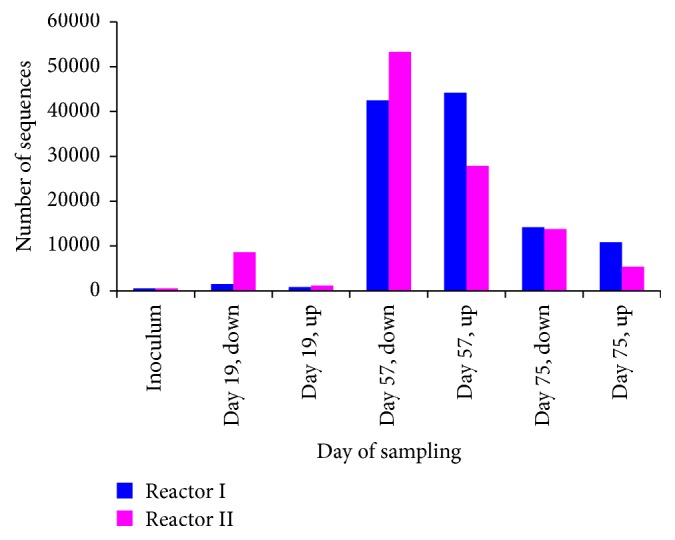
Dynamics of the number of methanogenic reads sequenced during the time course of microalgae and sodium acetate AD. “Up” and “down” labels next to the day of sampling refer to the upper or bottom part of the sampled sludge bed.

**Figure 7:**
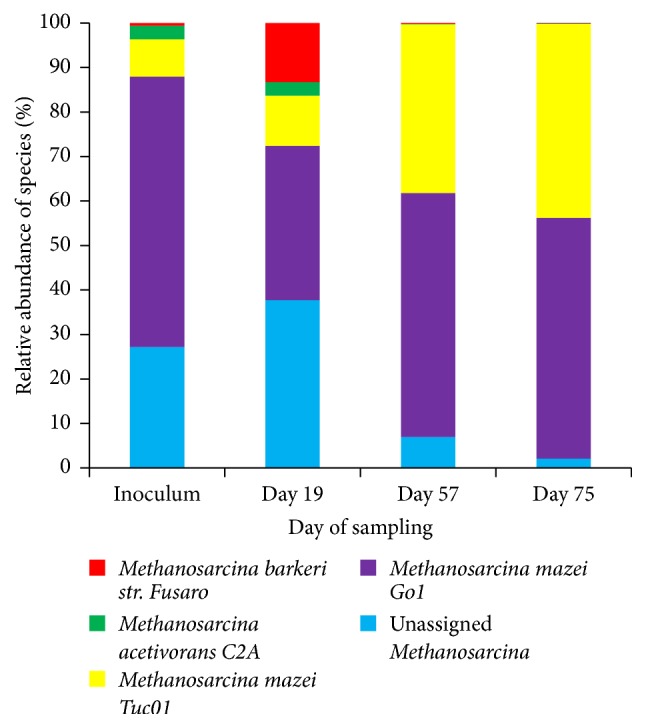
Dynamics of relative abundance of species members of Methanosarcinales order during the time course of microalgae and sodium acetate AD.

Clustering of the total number of reads related to the identified methanogenic species on the order level demonstrated a single order dominated system (Table 1). General dynamics of the number of total methanogenic reads sequenced during the time course of AD is depicted in Figure 6. Results presented in Figure 6 indicate an increase in the number of methanogen-related reads during the time course of the AD. A high number of methanogenic reads identified on the 57th day of reactors operation is in agreement with the exponential increase in the amount of biogas being produced after this time point (Figure 1). Assessment of the species distribution in the identified dominant Methanosarcinales order revealed a single-species dominant methanogenic system (Figure 7), with* Methanosarcina mazei* leading to the digestion of microalgae and sodium acetate on the last stage of anaerobic digestion, methanogenesis.

## 4. Discussion

In this study, the microbial dynamics governing anaerobic digestion of microalgal biomass and sodium acetate were analyzed. Use of metagenome sequencing revealed a dynamic shift in bacterial community structures over the time course of AD. Initial bacterial inoculum for start-up of the AD process in a UASB reactor was taken from anaerobic sediments in the Logan Lagoons (a wastewater treatment facility in Logan, Utah). These sediments are thought to contribute to the exceptional performance of Logan Lagoons wastewater treatment facility for over 40 years [43]. Testing this exceptional productivity of sediments on AD of microalgal biomass (which accumulates in the lagoons and is a significant carbon source for the microorganisms) led to the identification of the key microorganisms contributing to the hydrolysis of microalgal biomass and subsequent methane production in this study. Since microalgal biomass in Logan Lagoons has a low natural C/N ratio (5/1) that is not sufficient for successful anaerobic digestion (batch preliminary experiments [44]), microalgae were mixed with sodium acetate to increase C/N ratio to 21/1.

To better assess the composition of the microbial community during AD of microalgal biomass and sodium acetate, duplicate UASB reactors were constructed, each bearing two sampling ports located at the bottom and upper parts of the sludge bed. Such sampling allowed examining the influence of a direct exposure of microorganisms to the substrate at the bottom of the reactor, contrary to the exposure of microorganisms at the upper part of the sludge bed to the already predigested substrate (by the microorganisms at the bottom part of the sludge bed).

Results demonstrated a fairly close distribution of microorganisms across the sludge bed (Supplemental Figure 2), with the only exception of the number of assigned reads to the order of Clostridiales during the start-up of the reactor (19 days of operation) and the order of Pseudomonadales at day 75 of reactor operation (Figure 4). Even though bottom and upper sampling ports of sludge bed are located 20 cm apart, this distance can indeed differentiate between two different stages of anaerobic digestion: initial hydrolysis and acidogenesis/acetogenesis. A dominant system comprising Clostridiales at day 57 and day 75 with the second dominant order of Pseudomonadales can be observed from Figure 7. Clostridiales are also dominant at day 19 (the bottom part), and Pseudomonadales can be given no exceptional role. Comparison of dynamics changes in the number of assigned reads to those two orders reveals that amount of Clostridiales stayed relatively the same after reactor start-up (day 19), while amount of Pseudomonadales increased by 370% at the bottom part of the sludge bed and by 1727% at the upper part of sludge bed.

Such a dynamic change in the number of assigned reads to the order of Pseudomonadales during the start-up period of a UASB reactor suggests that supplied substrate for AD (microalgal biomass and sodium acetate) was a trigger of bacterial growth of members of the Pseudomonadales order. Previous studies also report increased amount of Pseudomonadales in AD of microalgal biomass [45].

In addition to the change in the number of Pseudomonadales-assigned reads, the start-up period boosted growth of Enterobacteriales and Bacteroidales (Figure 4). Prevalence of Bacteroidales on the 19th day of AD correlates with the suggestion that this is a hydrolysis phase, and Bacteroidales generally comprise genera of bacteria with distinct saccharolytic activities, such as* Bacteroides* that produce acetic acid as an end product [46]. These bacteria are often found at the initial stages of anaerobic digestion [47, 48].

For two other orders, Pseudomonadales possess mostly nonfermenting metabolism, while Enterobacteriales are fermenters and can produce fatty acids and lactic acids. Genera of* Pseudomonas* and* Enterobacter *have been detected at high numbers in eutrophicated lakes with microalgal blooms [49–51]. Members of* Pseudomonas* spp. were recently ascribed to have distinct microalgal cell degrading abilities [52] and ability to degrade microalgal toxins, microcystins [53–56]. A combined alga-lytic activity of two members of Pseudomonadales and Enterobacteriales orders,* Pseudomonas aeruginosa *and* Citrobacter freundii, *has been reported for cyanobacteria that were collected from municipal wastewater lagoon [57]. While alga-lytic activity of* Pseudomonas* spp. predominantly aimed at cyanobacteria, alga-lytic activity of* Enterobacter *spp. expands also to green algae [58–60]. Since both cyanobacteria and green algae were present in the feedstock for the described here AD in a UASB reactor (see Materials and Methods), we can suggest that members of Pseudomonadales and Enterobacteriales orders have an alga-lytic activity towards microalgal biomass from Logan Wastewater Lagoons.

Alga-lytic activity might not only be characteristic for* Pseudomonas* and* Enterobacter* but was also observed for other members of our bacterial community in a UASB reactor. Reads of the Thermotogales order were identified during the presumably acidogenic-methanogenic phase of AD (57th day, Figure 4), where, due to the continuous flow of microalgal biomass and sodium acetate, hydrolysis still takes place. Thermotogales were previously reported to have an alga-lytic activity towards green microalgae [61, 62]. This lytic behavior might be managed by the extracellular enzymes of Thermotogales, amylases, which make it possible for the bacterium to ferment carbohydrate polymers of microalgal biomass to hydrogen [63, 64]. However, to make this process happen, micoralgal biomass should be initially disrupted to release carbohydrates. Therefore, if considering that initial microalgal biomass disruption occurred during the initial hydrolysis phase of AD during start-up of reactors (samples taken on day 19) and bacteria from Proteobacteria phylum have successfully initiated the degradation process, we would expect secondary hydrolyzing agents, such as Thermotogales, to be active after some delay from the initial hydrolytic phase. Also, since Thermotogales convert microalgal carbohydrates into the hydrogen, hydrogen can be supplied to methanogenic bacteria that were detected in the abundance at the 57th day of AD (Figure 6).

Another order of bacteria detected at the initial stage of AD (day 19) is Synergistales. Presence of these bacteria at the hydrolytic stage of AD can be due to the metabolic preferences of these bacteria to consume amino acids and complex proteinaceous compounds [65]. Synergistales were also previously reported to be present in similar environments as a UASB reactor, wastewater treatment lagoons, and anaerobic sludge [3, 66]. Detection of Synergistales in the anaerobic digestion is in agreement with previously published data by Delbès et al. [67], but exact role of these bacteria in AD is not yet known.

The presence of specific alga-lytic bacterial orders in our reactor is attributed to the fact that initial inoculum for AD was taken from the sediments in the Logan Wastewater Lagoons. An observed high degree of decomposition of microalgal biomass (average COD removal rate of 79%, as observed by Soboh et al. [40]) can be explained with a long term adaptation of the facultative aerobic microorganisms to the algal residues present at the bottom of the lagoons ponds (48 years of Logan Wastewater Lagoons operation) and selection of species that are able to efficiently degrade microalgal biomass to maintain stability of the Lagoon system. Previous studies have pointed to the specific recalcitrance of microalgal cells to AD, which is usually conducted with either acid or temperature pretreatment of microalgal biomass [19, 21, 28, 29, 68–72]. These studies also demonstrated a methane composition of up to 60% in a produced biogas from fermentation of microalgal biomass and 73% in codigestion with swine manure. In our case, produced biogas had on average 85% methane composition [40], which might be because of a more intense decomposition of microalgal biomass by alga-lytic bacteria identified at the 19th day of AD in a UASB reactor.

Moving deeper into the process of AD, to the microbial community on day 57, Clostridiales order occupies the most attention. An increase in the amount of Clostridiales at this sampling time (Figure 4) could be due to the high content of polysaccharides in the hydrolyzed microalgal biomass. Generally, Firmicutes are prevalent at the acetogenic/acidogenic stages of anaerobic digestion due to their ability to ferment sugars and amino acids into acetic and lactic acid [3, 73, 74]. Members of Clostridiales order were also reported in abundance in other microalgae digestion experiments [45]. Previous studies on Logan Lagoons microbiome have identified a high diversity of* Clostridium* spp. and a dominance of a Clostridiales order [32]. The role of Clostridiales in the AD of microalgal biomass and sodium acetate can be relevant to both hydrolysis and acetogenic stages, since initial high percentage of Clostridiales in the inoculum (Figure 4) characterizes the sediments of the Logan Lagoons as a nurturing environment for these microorganisms. Ellis et al. tested* Clostridium saccharoperbutylacetonicum* on digestion of microalgal biomass from Logan Lagoons and did not observe any success, even though this bacterium has amylolytic activity towards starch-based polymers that are present in microalgal cell walls [75].* Clostridium saccharoperbutylacetonicum *was able to ferment microalgal biomass only after acidic-basic pretreatment of microalgae with sulfuric acid and sodium hydroxide [76]. This leads to a thought that* Clostridium* spp. identified in our study might indeed be involved in the second step of AD of microalgal biomass and a pretreatment step (by other bacterial consortia) is vital for the final conversion of microalgal biomass into the set of alcohols, such as ethanol, acetone, and butanol.

Acidogenic/acetogenic phase of AD in our study has revealed the presence of another bacterial taxa, in addition to the Clostridiales order. Sulfate-reducing bacteria, members of Desulfovibrionales order, were detected at the 57th day (Figure 4). With regard to the dynamics of methanogenic bacteria population throughout AD, as depicted in Figure 3, and presence of Desulfovibrionales at the same time point, a competitive interaction for substrate might take place between two types of anaerobic microorganisms [77, 78].

Possible way to communicate this observation is that the higher number of sulfate-reducers in the upper sampling point at day 19th correlates with the higher thermodynamic possibility of sodium acetate assimilation via sulfate-reduction, rather than via methanogenesis (Table 3). The decrease in the relative abundance of sulfate-reducers later during the AD (Figure 4) could be due to the exhaustion of sulfate in the bioreactor and sulfate is electron acceptor during substrate assimilation by Desulfovibrionales (initial sulfate might have come with the inoculum from sediments in the lagoons and is not present in the supplied microalgal biomass during AD) [79]. Simultaneously we observed a shift from low number of methanogenic sequences to the high number later during the AD (day 57th, Figure 6). Ozuolmez and colleagues observed a similar shift from high numbers of sulfate-reducers to higher numbers of methanogens during a cocultivation of* Methanosaeta concilii* and* Desulfovibrio vulgaris* on acetate [80].

With respect to the methanogenesis and its outcompeting of sulfate-reduction, our results demonstrate that AD of microalgal biomass with sodium acetate was selective towards a single-species dominant methanogenic system.* Methanosarcina mazei *was prominently proliferating at the 57th day of AD (Table 2, Figure 3). Presence of* Methanosarcina* spp. in anaerobic reactors is common due to their high growth rates, rapid consumption of a broad spectrum of substrates (acetate, methanol, and hydrogen), and a high stress resistance to the fluctuations in the anaerobic digester, such as pH and OLR [78, 81–84]. A particular dominance of* Methanosarcina mazei *in the UASB reactor fed with microalgal biomass and sodium acetate has not yet been reported by others.

Possible explanations on why* M. mazei *was dominant can be due to several factors based on the nature of the supplied substrate (microalgal biomass and sodium acetate): (1) addition of sodium acetate as a feedstock into the reactor creates conditions of elevated amount of acetate that can only be consumed by species of methanogen with high growth rates and high acetate turnover rates, such as* Methanosarcina mazei* [85]; (2) slight fluctuations were observed in the pH during the AD [40] and* Methanosarcina mazei* have been previously reported to be able to withstand even higher pH fluctuations for a short period of time, as opposed to such species of* Methanosarcina *as* Methanosarcina barkeri* [86].

**Figure 8:**
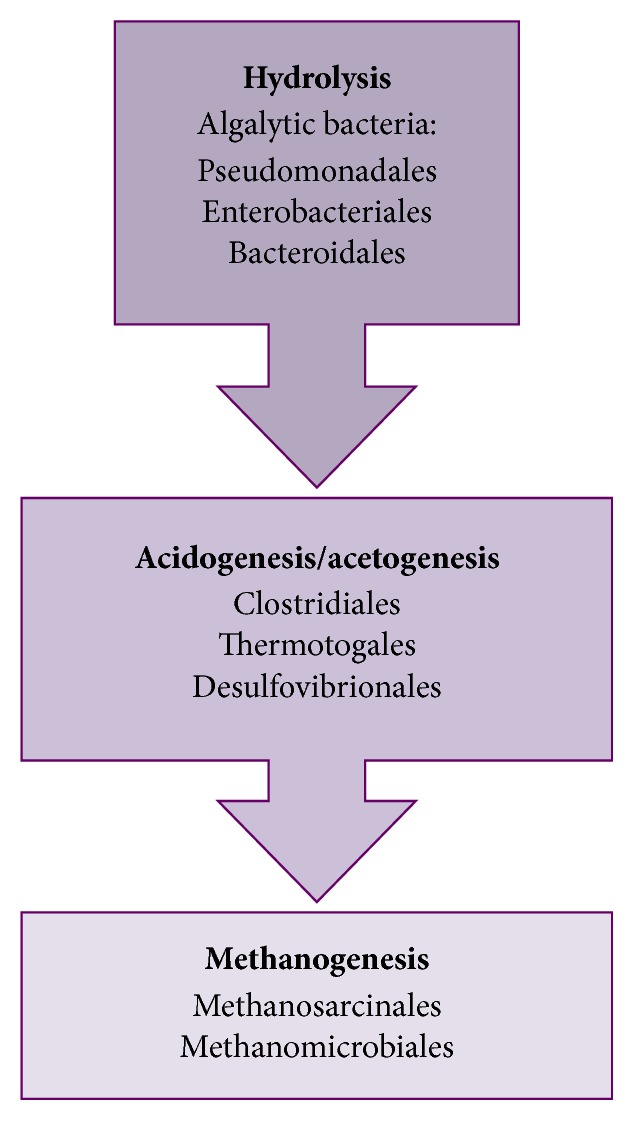
Proposed set of key microorganisms involved in anaerobic digestion of microalgal biomass and sodium acetate.

To summarize the analysis of metagenome during anaerobic digestion of microalgal biomass and sodium acetate, a general flow of microbial dynamics is proposed in Figure 8.

## 5. Conclusions

A demonstrated analysis of a bacterial metagenome during anaerobic digestion of microalgal biomass and sodium acetate has provided a valuable insight into complex microbial interactions and can be used for further studies leading to cultivation of key microorganisms of interest. For microalgal biomass digestion, metagenome analysis was especially valuable to identify potential alga-lytic bacteria (members of the orders Bacteroidales, Pseudomonadales, and Enterobacteriales), and further studies will include isolation of this poorly studied group of microorganisms. Identification of new bacteria influencing anaerobic digestion of previously thought recalcitrant microalgal biomass has practical applications for increasing yields of biogas from such an abundant and sustainable type of substrate.

## Supplementary Material

Supplementary Table 1: Primers used in the reported study.
Supplementary Table 2: Core set of OTUs, shared among all sampling time points during the anaerobic digestion of microalgal biomass and sodium acetate in duplicates of UASB reactors.
Supplemental Figure 1: Schematics of the Upflow Anaerobic Sludge Blanket reactor (UASB) used in the study.
Supplemental Figure 2: Heatmap, calculated with jclass algorithm in MOTHUR, representing beta-diversity (internal compositional heterogeneity) of samples taken at the same time point from two reactors. Labels “Uni” represent 16S rRNA universal primer set used in the study. Red-colored scale from 0.0 to 1.0 should be interpreted as the 1.0 bright color correspond to the closely related samples. Opposite is true for the 0.0 marking and dark red color.
Supplementary Figure 3: A. General workflow anaerobic digestion of microalgal biomass and analysis of eubacterial and methanogenic communities. B. Workflow for the sequence analysis and identification of microorganisms (via MOTHUR MiSeq_SOP).

## Figures and Tables

**Table 1:** Calculation of significance of 16S rRNA samples separation at different time points of anaerobic digestion.

Groups	*W*Score	*W*Sig
Day 19–inoculum	1	<0.0010
Day 19–day 57	1	0.017
Inoculum–day 57	1	<0.0010
Day 19–day 75	0.602815	0.018
Inoculum–day 75	0.895479	<0.0010
Day 57–day 75	0.404311	<0.0010

**Table 2:** Total number of reads related to the identified methanogenic species during the course of AD of microalgae and sodium acetate. “Up” and “down” labels next to the day of sampling refer to the upper or bottom part of the sampled sludge bed. Data is combined for both reactors.

	Inoculum	Day 19, up	Day 19, down	Day 57, up	Day 57, down	Day 75, up	Day 75, down
Methanobacteriales	0	0	1	15	0	7	2
Methanocellales	0	1	0	0	0	0	0
Methanomicrobiales	9	14	27	12	5	0	0
Methanosarcinales	61	1466	808	42459	44169	14166	10829

**Table 3:** Free Gibbs energy required for the assimilation of acetate via sulfate-reduction and methanogenesis [31].

*Acetate assimilation via sulfate reduction:*	
CH_3_COO^−^ + SO_4_^2−^ → 2HCO_3_^−^ + HS^−^	Δ*G*_0_ = −47.6 kJ mol^−1^
*Acetate assimilation via methanogenesis:*	
4CH_3_COO^−^ → 3CH_4_ + HCO_3_^−^	Δ*G*_0_ = −31.0 kJ mol^−1^
