# Supplementary material for: Qualitative Analysis of Microbial Dynamics during Anaerobic Digestion of Microalgal Biomass in a UASB Reactor
Source: Int J Microbiol. 2017 Nov 13;2017:5291283. doi: 10.1155/2017/5291283 (PMC5702946; doi:10.1155/2017/5291283)
Supplement: Supplementary file 1 — Supplementary Table 1: Primers used in the reported study. Supplementary Table 2: Core set of OTUs, shared among all sampling time points during the anaerobic digestion of microalgal biomass and sodium acetate in duplicates of UASB reactors. Supplemental Figure 1: Schematics of the Upflow Anaerobic Sludge Blanket reactor (UASB) used in the study. Supplemental Figure 2: Heatmap, calculated with jclass algorithm in MOTHUR, representing beta-diversity (internal compositional heterogeneity) of samples taken at the same time point from two reactors. Labels “Uni” represent 16S rRNA universal primer set used in the study. Red-colored scale from 0.0 to 1.0 should be interpreted as the 1.0 bright color correspond to the closely related samples. Opposite is true for the 0.0 marking and dark red color. Supplementary Figure 3: A. General workflow anaerobic digestion of microalgal biomass and analysis of eubacterial and methanogenic communities. B. Workflow for the sequence analysis and identification of microorganisms (via MOTHUR MiSeq_SOP). [file 5291283.f1.zip › Supplementary materials/Supplementary Table 2_IJMICRO_2043581.docx]

Supplementary Table 2. Core set of OTUs, shared among all sampling time points during the anaerobic digestion of microalgal biomass and sodium acetate in duplicates of UASB reactors.

| **OTU** | **Taxonomy** | **Sample** | **Number of sequences detected** |
| --- | --- | --- | --- |
| Otu001 | Bacteria(100);unclassified(100);unclassified(100);unclassified(100);unclassified(100);unclassified(100); | RI(1t)downUni | 17318 |
| Otu001 | Bacteria(100);unclassified(100);unclassified(100);unclassified(100);unclassified(100);unclassified(100); | RI(1t)upUni | 38129 |
| Otu001 | Bacteria(100);unclassified(100);unclassified(100);unclassified(100);unclassified(100);unclassified(100); | RI(2t)downUni | 9214 |
| Otu001 | Bacteria(100);unclassified(100);unclassified(100);unclassified(100);unclassified(100);unclassified(100); | RI(2t)upUni | 59969 |
| Otu001 | Bacteria(100);unclassified(100);unclassified(100);unclassified(100);unclassified(100);unclassified(100); | RI(3t)downUni | 9422 |
| Otu001 | Bacteria(100);unclassified(100);unclassified(100);unclassified(100);unclassified(100);unclassified(100); | RI(3t)upUni | 12850 |
| Otu001 | Bacteria(100);unclassified(100);unclassified(100);unclassified(100);unclassified(100);unclassified(100); | RII(1t)downUni | 54308 |
| Otu001 | Bacteria(100);unclassified(100);unclassified(100);unclassified(100);unclassified(100);unclassified(100); | RII(1t)upUni | 26439 |
| Otu001 | Bacteria(100);unclassified(100);unclassified(100);unclassified(100);unclassified(100);unclassified(100); | RII(2t)downUni | 11157 |
| Otu001 | Bacteria(100);unclassified(100);unclassified(100);unclassified(100);unclassified(100);unclassified(100); | RII(2t)upUni | 37073 |
| Otu001 | Bacteria(100);unclassified(100);unclassified(100);unclassified(100);unclassified(100);unclassified(100); | RII(3t)downUni | 16299 |
| Otu001 | Bacteria(100);unclassified(100);unclassified(100);unclassified(100);unclassified(100);unclassified(100); | RII(3t)upUni | 32126 |
| Otu001 | Bacteria(100);unclassified(100);unclassified(100);unclassified(100);unclassified(100);unclassified(100); | S1Uni | 86711 |
| Otu001 | Bacteria(100);unclassified(100);unclassified(100);unclassified(100);unclassified(100);unclassified(100); | S2Uni | 93334 |
| Otu002 | Bacteria(100);"Bacteroidetes"(100);unclassified(100);unclassified(100);unclassified(100);unclassified(100); | RI(1t)downUni | 20503 |
| Otu002 | Bacteria(100);"Bacteroidetes"(100);unclassified(100);unclassified(100);unclassified(100);unclassified(100); | RI(1t)upUni | 57050 |
| Otu002 | Bacteria(100);"Bacteroidetes"(100);unclassified(100);unclassified(100);unclassified(100);unclassified(100); | RI(2t)downUni | 43233 |
| Otu002 | Bacteria(100);"Bacteroidetes"(100);unclassified(100);unclassified(100);unclassified(100);unclassified(100); | RI(2t)upUni | 206045 |
| Otu002 | Bacteria(100);"Bacteroidetes"(100);unclassified(100);unclassified(100);unclassified(100);unclassified(100); | RI(3t)downUni | 53970 |
| Otu002 | Bacteria(100);"Bacteroidetes"(100);unclassified(100);unclassified(100);unclassified(100);unclassified(100); | RI(3t)upUni | 19174 |
| Otu002 | Bacteria(100);"Bacteroidetes"(100);unclassified(100);unclassified(100);unclassified(100);unclassified(100); | RII(1t)downUni | 69808 |
| Otu002 | Bacteria(100);"Bacteroidetes"(100);unclassified(100);unclassified(100);unclassified(100);unclassified(100); | RII(1t)upUni | 50922 |
| Otu002 | Bacteria(100);"Bacteroidetes"(100);unclassified(100);unclassified(100);unclassified(100);unclassified(100); | RII(2t)downUni | 32267 |
| Otu002 | Bacteria(100);"Bacteroidetes"(100);unclassified(100);unclassified(100);unclassified(100);unclassified(100); | RII(2t)upUni | 132603 |
| Otu002 | Bacteria(100);"Bacteroidetes"(100);unclassified(100);unclassified(100);unclassified(100);unclassified(100); | RII(3t)downUni | 121588 |
| Otu002 | Bacteria(100);"Bacteroidetes"(100);unclassified(100);unclassified(100);unclassified(100);unclassified(100); | RII(3t)upUni | 43982 |
| Otu002 | Bacteria(100);"Bacteroidetes"(100);unclassified(100);unclassified(100);unclassified(100);unclassified(100); | S1Uni | 20617 |
| Otu002 | Bacteria(100);"Bacteroidetes"(100);unclassified(100);unclassified(100);unclassified(100);unclassified(100); | S2Uni | 21785 |
| Otu003 | Bacteria(100);Firmicutes(100);Clostridia(100);Clostridiales(100);unclassified(100);unclassified(100); | RI(1t)downUni | 1872 |
| Otu003 | Bacteria(100);Firmicutes(100);Clostridia(100);Clostridiales(100);unclassified(100);unclassified(100); | RI(1t)upUni | 3296 |
| Otu003 | Bacteria(100);Firmicutes(100);Clostridia(100);Clostridiales(100);unclassified(100);unclassified(100); | RI(2t)downUni | 3378 |
| Otu003 | Bacteria(100);Firmicutes(100);Clostridia(100);Clostridiales(100);unclassified(100);unclassified(100); | RI(2t)upUni | 19376 |
| Otu003 | Bacteria(100);Firmicutes(100);Clostridia(100);Clostridiales(100);unclassified(100);unclassified(100); | RI(3t)downUni | 6147 |
| Otu003 | Bacteria(100);Firmicutes(100);Clostridia(100);Clostridiales(100);unclassified(100);unclassified(100); | RI(3t)upUni | 7100 |
| Otu003 | Bacteria(100);Firmicutes(100);Clostridia(100);Clostridiales(100);unclassified(100);unclassified(100); | RII(1t)downUni | 6558 |
| Otu003 | Bacteria(100);Firmicutes(100);Clostridia(100);Clostridiales(100);unclassified(100);unclassified(100); | RII(1t)upUni | 4108 |
| Otu003 | Bacteria(100);Firmicutes(100);Clostridia(100);Clostridiales(100);unclassified(100);unclassified(100); | RII(2t)downUni | 3744 |
| Otu003 | Bacteria(100);Firmicutes(100);Clostridia(100);Clostridiales(100);unclassified(100);unclassified(100); | RII(2t)upUni | 14662 |
| Otu003 | Bacteria(100);Firmicutes(100);Clostridia(100);Clostridiales(100);unclassified(100);unclassified(100); | RII(3t)downUni | 10965 |
| Otu003 | Bacteria(100);Firmicutes(100);Clostridia(100);Clostridiales(100);unclassified(100);unclassified(100); | RII(3t)upUni | 10012 |
| Otu003 | Bacteria(100);Firmicutes(100);Clostridia(100);Clostridiales(100);unclassified(100);unclassified(100); | S1Uni | 1980 |
| Otu003 | Bacteria(100);Firmicutes(100);Clostridia(100);Clostridiales(100);unclassified(100);unclassified(100); | S2Uni | 2512 |
| Otu004 | Bacteria(100);"Proteobacteria"(100);Gammaproteobacteria(100);unclassified(100);unclassified(100);unclassified(100); | RI(1t)downUni | 330 |
| Otu004 | Bacteria(100);"Proteobacteria"(100);Gammaproteobacteria(100);unclassified(100);unclassified(100);unclassified(100); | RI(1t)upUni | 576 |
| Otu004 | Bacteria(100);"Proteobacteria"(100);Gammaproteobacteria(100);unclassified(100);unclassified(100);unclassified(100); | RI(2t)downUni | 69 |
| Otu004 | Bacteria(100);"Proteobacteria"(100);Gammaproteobacteria(100);unclassified(100);unclassified(100);unclassified(100); | RI(2t)upUni | 988 |
| Otu004 | Bacteria(100);"Proteobacteria"(100);Gammaproteobacteria(100);unclassified(100);unclassified(100);unclassified(100); | RI(3t)downUni | 93 |
| Otu004 | Bacteria(100);"Proteobacteria"(100);Gammaproteobacteria(100);unclassified(100);unclassified(100);unclassified(100); | RI(3t)upUni | 131 |
| Otu004 | Bacteria(100);"Proteobacteria"(100);Gammaproteobacteria(100);unclassified(100);unclassified(100);unclassified(100); | RII(1t)downUni | 1143 |
| Otu004 | Bacteria(100);"Proteobacteria"(100);Gammaproteobacteria(100);unclassified(100);unclassified(100);unclassified(100); | RII(1t)upUni | 781 |
| Otu004 | Bacteria(100);"Proteobacteria"(100);Gammaproteobacteria(100);unclassified(100);unclassified(100);unclassified(100); | RII(2t)downUni | 98 |
| Otu004 | Bacteria(100);"Proteobacteria"(100);Gammaproteobacteria(100);unclassified(100);unclassified(100);unclassified(100); | RII(2t)upUni | 380 |
| Otu004 | Bacteria(100);"Proteobacteria"(100);Gammaproteobacteria(100);unclassified(100);unclassified(100);unclassified(100); | RII(3t)downUni | 678 |
| Otu004 | Bacteria(100);"Proteobacteria"(100);Gammaproteobacteria(100);unclassified(100);unclassified(100);unclassified(100); | RII(3t)upUni | 4485 |
| Otu004 | Bacteria(100);"Proteobacteria"(100);Gammaproteobacteria(100);unclassified(100);unclassified(100);unclassified(100); | S1Uni | 5057 |
| Otu004 | Bacteria(100);"Proteobacteria"(100);Gammaproteobacteria(100);unclassified(100);unclassified(100);unclassified(100); | S2Uni | 6131 |
| Otu005 | Bacteria(100);Firmicutes(100);unclassified(100);unclassified(100);unclassified(100);unclassified(100); | RI(1t)downUni | 3322 |
| Otu005 | Bacteria(100);Firmicutes(100);unclassified(100);unclassified(100);unclassified(100);unclassified(100); | RI(1t)upUni | 3094 |
| Otu005 | Bacteria(100);Firmicutes(100);unclassified(100);unclassified(100);unclassified(100);unclassified(100); | RI(2t)downUni | 1218 |
| Otu005 | Bacteria(100);Firmicutes(100);unclassified(100);unclassified(100);unclassified(100);unclassified(100); | RI(2t)upUni | 8148 |
| Otu005 | Bacteria(100);Firmicutes(100);unclassified(100);unclassified(100);unclassified(100);unclassified(100); | RI(3t)downUni | 1169 |
| Otu005 | Bacteria(100);Firmicutes(100);unclassified(100);unclassified(100);unclassified(100);unclassified(100); | RI(3t)upUni | 992 |
| Otu005 | Bacteria(100);Firmicutes(100);unclassified(100);unclassified(100);unclassified(100);unclassified(100); | RII(1t)downUni | 5510 |
| Otu005 | Bacteria(100);Firmicutes(100);unclassified(100);unclassified(100);unclassified(100);unclassified(100); | RII(1t)upUni | 5958 |
| Otu005 | Bacteria(100);Firmicutes(100);unclassified(100);unclassified(100);unclassified(100);unclassified(100); | RII(2t)downUni | 1537 |
| Otu005 | Bacteria(100);Firmicutes(100);unclassified(100);unclassified(100);unclassified(100);unclassified(100); | RII(2t)upUni | 6270 |
| Otu005 | Bacteria(100);Firmicutes(100);unclassified(100);unclassified(100);unclassified(100);unclassified(100); | RII(3t)downUni | 2163 |
| Otu005 | Bacteria(100);Firmicutes(100);unclassified(100);unclassified(100);unclassified(100);unclassified(100); | RII(3t)upUni | 2569 |
| Otu005 | Bacteria(100);Firmicutes(100);unclassified(100);unclassified(100);unclassified(100);unclassified(100); | S1Uni | 1603 |
| Otu005 | Bacteria(100);Firmicutes(100);unclassified(100);unclassified(100);unclassified(100);unclassified(100); | S2Uni | 1647 |
| Otu006 | Bacteria(100);Firmicutes(100);Clostridia(100);Clostridiales(100);Syntrophomonadaceae(100);unclassified(100); | RI(1t)downUni | 160 |
| Otu006 | Bacteria(100);Firmicutes(100);Clostridia(100);Clostridiales(100);Syntrophomonadaceae(100);unclassified(100); | RI(1t)upUni | 94 |
| Otu006 | Bacteria(100);Firmicutes(100);Clostridia(100);Clostridiales(100);Syntrophomonadaceae(100);unclassified(100); | RI(2t)downUni | 326 |
| Otu006 | Bacteria(100);Firmicutes(100);Clostridia(100);Clostridiales(100);Syntrophomonadaceae(100);unclassified(100); | RI(2t)upUni | 5006 |
| Otu006 | Bacteria(100);Firmicutes(100);Clostridia(100);Clostridiales(100);Syntrophomonadaceae(100);unclassified(100); | RI(3t)downUni | 320 |
| Otu006 | Bacteria(100);Firmicutes(100);Clostridia(100);Clostridiales(100);Syntrophomonadaceae(100);unclassified(100); | RI(3t)upUni | 588 |
| Otu006 | Bacteria(100);Firmicutes(100);Clostridia(100);Clostridiales(100);Syntrophomonadaceae(100);unclassified(100); | RII(1t)downUni | 336 |
| Otu006 | Bacteria(100);Firmicutes(100);Clostridia(100);Clostridiales(100);Syntrophomonadaceae(100);unclassified(100); | RII(1t)upUni | 219 |
| Otu006 | Bacteria(100);Firmicutes(100);Clostridia(100);Clostridiales(100);Syntrophomonadaceae(100);unclassified(100); | RII(2t)downUni | 527 |
| Otu006 | Bacteria(100);Firmicutes(100);Clostridia(100);Clostridiales(100);Syntrophomonadaceae(100);unclassified(100); | RII(2t)upUni | 1740 |
| Otu006 | Bacteria(100);Firmicutes(100);Clostridia(100);Clostridiales(100);Syntrophomonadaceae(100);unclassified(100); | RII(3t)downUni | 1269 |
| Otu006 | Bacteria(100);Firmicutes(100);Clostridia(100);Clostridiales(100);Syntrophomonadaceae(100);unclassified(100); | RII(3t)upUni | 1586 |
| Otu006 | Bacteria(100);Firmicutes(100);Clostridia(100);Clostridiales(100);Syntrophomonadaceae(100);unclassified(100); | S1Uni | 42 |
| Otu006 | Bacteria(100);Firmicutes(100);Clostridia(100);Clostridiales(100);Syntrophomonadaceae(100);unclassified(100); | S2Uni | 48 |
| Otu007 | Bacteria(100);"Proteobacteria"(100);unclassified(100);unclassified(100);unclassified(100);unclassified(100); | RI(1t)downUni | 592 |
| Otu007 | Bacteria(100);"Proteobacteria"(100);unclassified(100);unclassified(100);unclassified(100);unclassified(100); | RI(1t)upUni | 215 |
| Otu007 | Bacteria(100);"Proteobacteria"(100);unclassified(100);unclassified(100);unclassified(100);unclassified(100); | RI(2t)downUni | 313 |
| Otu007 | Bacteria(100);"Proteobacteria"(100);unclassified(100);unclassified(100);unclassified(100);unclassified(100); | RI(2t)upUni | 1054 |
| Otu007 | Bacteria(100);"Proteobacteria"(100);unclassified(100);unclassified(100);unclassified(100);unclassified(100); | RI(3t)downUni | 89 |
| Otu007 | Bacteria(100);"Proteobacteria"(100);unclassified(100);unclassified(100);unclassified(100);unclassified(100); | RI(3t)upUni | 49 |
| Otu007 | Bacteria(100);"Proteobacteria"(100);unclassified(100);unclassified(100);unclassified(100);unclassified(100); | RII(1t)downUni | 1689 |
| Otu007 | Bacteria(100);"Proteobacteria"(100);unclassified(100);unclassified(100);unclassified(100);unclassified(100); | RII(1t)upUni | 574 |
| Otu007 | Bacteria(100);"Proteobacteria"(100);unclassified(100);unclassified(100);unclassified(100);unclassified(100); | RII(2t)downUni | 395 |
| Otu007 | Bacteria(100);"Proteobacteria"(100);unclassified(100);unclassified(100);unclassified(100);unclassified(100); | RII(2t)upUni | 1226 |
| Otu007 | Bacteria(100);"Proteobacteria"(100);unclassified(100);unclassified(100);unclassified(100);unclassified(100); | RII(3t)downUni | 405 |
| Otu007 | Bacteria(100);"Proteobacteria"(100);unclassified(100);unclassified(100);unclassified(100);unclassified(100); | RII(3t)upUni | 224 |
| Otu007 | Bacteria(100);"Proteobacteria"(100);unclassified(100);unclassified(100);unclassified(100);unclassified(100); | S1Uni | 10692 |
| Otu007 | Bacteria(100);"Proteobacteria"(100);unclassified(100);unclassified(100);unclassified(100);unclassified(100); | S2Uni | 12040 |
| Otu008 | Bacteria(100);Firmicutes(100);Clostridia(100);Clostridiales(100);Ruminococcaceae(100);unclassified(100); | RI(1t)downUni | 1716 |
| Otu008 | Bacteria(100);Firmicutes(100);Clostridia(100);Clostridiales(100);Ruminococcaceae(100);unclassified(100); | RI(1t)upUni | 2527 |
| Otu008 | Bacteria(100);Firmicutes(100);Clostridia(100);Clostridiales(100);Ruminococcaceae(100);unclassified(100); | RI(2t)downUni | 2327 |
| Otu008 | Bacteria(100);Firmicutes(100);Clostridia(100);Clostridiales(100);Ruminococcaceae(100);unclassified(100); | RI(2t)upUni | 20453 |
| Otu008 | Bacteria(100);Firmicutes(100);Clostridia(100);Clostridiales(100);Ruminococcaceae(100);unclassified(100); | RI(3t)downUni | 1159 |
| Otu008 | Bacteria(100);Firmicutes(100);Clostridia(100);Clostridiales(100);Ruminococcaceae(100);unclassified(100); | RI(3t)upUni | 861 |
| Otu008 | Bacteria(100);Firmicutes(100);Clostridia(100);Clostridiales(100);Ruminococcaceae(100);unclassified(100); | RII(1t)downUni | 5650 |
| Otu008 | Bacteria(100);Firmicutes(100);Clostridia(100);Clostridiales(100);Ruminococcaceae(100);unclassified(100); | RII(1t)upUni | 2955 |
| Otu008 | Bacteria(100);Firmicutes(100);Clostridia(100);Clostridiales(100);Ruminococcaceae(100);unclassified(100); | RII(2t)downUni | 2911 |
| Otu008 | Bacteria(100);Firmicutes(100);Clostridia(100);Clostridiales(100);Ruminococcaceae(100);unclassified(100); | RII(2t)upUni | 12468 |
| Otu008 | Bacteria(100);Firmicutes(100);Clostridia(100);Clostridiales(100);Ruminococcaceae(100);unclassified(100); | RII(3t)downUni | 2773 |
| Otu008 | Bacteria(100);Firmicutes(100);Clostridia(100);Clostridiales(100);Ruminococcaceae(100);unclassified(100); | RII(3t)upUni | 3473 |
| Otu008 | Bacteria(100);Firmicutes(100);Clostridia(100);Clostridiales(100);Ruminococcaceae(100);unclassified(100); | S1Uni | 1308 |
| Otu008 | Bacteria(100);Firmicutes(100);Clostridia(100);Clostridiales(100);Ruminococcaceae(100);unclassified(100); | S2Uni | 1592 |
| Otu009 | Bacteria(100);"Proteobacteria"(100);Deltaproteobacteria(100);unclassified(100);unclassified(100);unclassified(100); | RI(1t)downUni | 744 |
| Otu009 | Bacteria(100);"Proteobacteria"(100);Deltaproteobacteria(100);unclassified(100);unclassified(100);unclassified(100); | RI(1t)upUni | 113 |
| Otu009 | Bacteria(100);"Proteobacteria"(100);Deltaproteobacteria(100);unclassified(100);unclassified(100);unclassified(100); | RI(2t)downUni | 139 |
| Otu009 | Bacteria(100);"Proteobacteria"(100);Deltaproteobacteria(100);unclassified(100);unclassified(100);unclassified(100); | RI(2t)upUni | 535 |
| Otu009 | Bacteria(100);"Proteobacteria"(100);Deltaproteobacteria(100);unclassified(100);unclassified(100);unclassified(100); | RI(3t)downUni | 57 |
| Otu009 | Bacteria(100);"Proteobacteria"(100);Deltaproteobacteria(100);unclassified(100);unclassified(100);unclassified(100); | RI(3t)upUni | 25 |
| Otu009 | Bacteria(100);"Proteobacteria"(100);Deltaproteobacteria(100);unclassified(100);unclassified(100);unclassified(100); | RII(1t)downUni | 2088 |
| Otu009 | Bacteria(100);"Proteobacteria"(100);Deltaproteobacteria(100);unclassified(100);unclassified(100);unclassified(100); | RII(1t)upUni | 598 |
| Otu009 | Bacteria(100);"Proteobacteria"(100);Deltaproteobacteria(100);unclassified(100);unclassified(100);unclassified(100); | RII(2t)downUni | 159 |
| Otu009 | Bacteria(100);"Proteobacteria"(100);Deltaproteobacteria(100);unclassified(100);unclassified(100);unclassified(100); | RII(2t)upUni | 500 |
| Otu009 | Bacteria(100);"Proteobacteria"(100);Deltaproteobacteria(100);unclassified(100);unclassified(100);unclassified(100); | RII(3t)downUni | 97 |
| Otu009 | Bacteria(100);"Proteobacteria"(100);Deltaproteobacteria(100);unclassified(100);unclassified(100);unclassified(100); | RII(3t)upUni | 63 |
| Otu009 | Bacteria(100);"Proteobacteria"(100);Deltaproteobacteria(100);unclassified(100);unclassified(100);unclassified(100); | S1Uni | 8844 |
| Otu009 | Bacteria(100);"Proteobacteria"(100);Deltaproteobacteria(100);unclassified(100);unclassified(100);unclassified(100); | S2Uni | 10264 |
| Otu010 | Bacteria(100);"Proteobacteria"(100);Gammaproteobacteria(100);Pseudomonadales(100);Pseudomonadaceae(100);unclassified(100); | RI(1t)downUni | 1761 |
| Otu010 | Bacteria(100);"Proteobacteria"(100);Gammaproteobacteria(100);Pseudomonadales(100);Pseudomonadaceae(100);unclassified(100); | RI(1t)upUni | 5553 |
| Otu010 | Bacteria(100);"Proteobacteria"(100);Gammaproteobacteria(100);Pseudomonadales(100);Pseudomonadaceae(100);unclassified(100); | RI(2t)downUni | 218 |
| Otu010 | Bacteria(100);"Proteobacteria"(100);Gammaproteobacteria(100);Pseudomonadales(100);Pseudomonadaceae(100);unclassified(100); | RI(2t)upUni | 1009 |
| Otu010 | Bacteria(100);"Proteobacteria"(100);Gammaproteobacteria(100);Pseudomonadales(100);Pseudomonadaceae(100);unclassified(100); | RI(3t)downUni | 60 |
| Otu010 | Bacteria(100);"Proteobacteria"(100);Gammaproteobacteria(100);Pseudomonadales(100);Pseudomonadaceae(100);unclassified(100); | RI(3t)upUni | 252 |
| Otu010 | Bacteria(100);"Proteobacteria"(100);Gammaproteobacteria(100);Pseudomonadales(100);Pseudomonadaceae(100);unclassified(100); | RII(1t)downUni | 1319 |
| Otu010 | Bacteria(100);"Proteobacteria"(100);Gammaproteobacteria(100);Pseudomonadales(100);Pseudomonadaceae(100);unclassified(100); | RII(1t)upUni | 13333 |
| Otu010 | Bacteria(100);"Proteobacteria"(100);Gammaproteobacteria(100);Pseudomonadales(100);Pseudomonadaceae(100);unclassified(100); | RII(2t)downUni | 155 |
| Otu010 | Bacteria(100);"Proteobacteria"(100);Gammaproteobacteria(100);Pseudomonadales(100);Pseudomonadaceae(100);unclassified(100); | RII(2t)upUni | 770 |
| Otu010 | Bacteria(100);"Proteobacteria"(100);Gammaproteobacteria(100);Pseudomonadales(100);Pseudomonadaceae(100);unclassified(100); | RII(3t)downUni | 472 |
| Otu010 | Bacteria(100);"Proteobacteria"(100);Gammaproteobacteria(100);Pseudomonadales(100);Pseudomonadaceae(100);unclassified(100); | RII(3t)upUni | 4179 |
| Otu010 | Bacteria(100);"Proteobacteria"(100);Gammaproteobacteria(100);Pseudomonadales(100);Pseudomonadaceae(100);unclassified(100); | S1Uni | 60 |
| Otu010 | Bacteria(100);"Proteobacteria"(100);Gammaproteobacteria(100);Pseudomonadales(100);Pseudomonadaceae(100);unclassified(100); | S2Uni | 69 |
| Otu011 | Bacteria(100);Firmicutes(100);Clostridia(100);Clostridiales(100);Syntrophomonadaceae(100);Syntrophomonas(100); | RI(1t)downUni | 1765 |
| Otu011 | Bacteria(100);Firmicutes(100);Clostridia(100);Clostridiales(100);Syntrophomonadaceae(100);Syntrophomonas(100); | RI(1t)upUni | 961 |
| Otu011 | Bacteria(100);Firmicutes(100);Clostridia(100);Clostridiales(100);Syntrophomonadaceae(100);Syntrophomonas(100); | RI(2t)downUni | 4577 |
| Otu011 | Bacteria(100);Firmicutes(100);Clostridia(100);Clostridiales(100);Syntrophomonadaceae(100);Syntrophomonas(100); | RI(2t)upUni | 50639 |
| Otu011 | Bacteria(100);Firmicutes(100);Clostridia(100);Clostridiales(100);Syntrophomonadaceae(100);Syntrophomonas(100); | RI(3t)downUni | 2325 |
| Otu011 | Bacteria(100);Firmicutes(100);Clostridia(100);Clostridiales(100);Syntrophomonadaceae(100);Syntrophomonas(100); | RI(3t)upUni | 2991 |
| Otu011 | Bacteria(100);Firmicutes(100);Clostridia(100);Clostridiales(100);Syntrophomonadaceae(100);Syntrophomonas(100); | RII(1t)downUni | 3767 |
| Otu011 | Bacteria(100);Firmicutes(100);Clostridia(100);Clostridiales(100);Syntrophomonadaceae(100);Syntrophomonas(100); | RII(1t)upUni | 2598 |
| Otu011 | Bacteria(100);Firmicutes(100);Clostridia(100);Clostridiales(100);Syntrophomonadaceae(100);Syntrophomonas(100); | RII(2t)downUni | 4982 |
| Otu011 | Bacteria(100);Firmicutes(100);Clostridia(100);Clostridiales(100);Syntrophomonadaceae(100);Syntrophomonas(100); | RII(2t)upUni | 22025 |
| Otu011 | Bacteria(100);Firmicutes(100);Clostridia(100);Clostridiales(100);Syntrophomonadaceae(100);Syntrophomonas(100); | RII(3t)downUni | 6890 |
| Otu011 | Bacteria(100);Firmicutes(100);Clostridia(100);Clostridiales(100);Syntrophomonadaceae(100);Syntrophomonas(100); | RII(3t)upUni | 7127 |
| Otu011 | Bacteria(100);Firmicutes(100);Clostridia(100);Clostridiales(100);Syntrophomonadaceae(100);Syntrophomonas(100); | S1Uni | 291 |
| Otu011 | Bacteria(100);Firmicutes(100);Clostridia(100);Clostridiales(100);Syntrophomonadaceae(100);Syntrophomonas(100); | S2Uni | 436 |
| Otu012 | Bacteria(100);"Thermotogae"(100);Thermotogae(100);Thermotogales(100);Thermotogaceae(100);unclassified(100); | RI(1t)downUni | 16 |
| Otu012 | Bacteria(100);"Thermotogae"(100);Thermotogae(100);Thermotogales(100);Thermotogaceae(100);unclassified(100); | RI(1t)upUni | 7 |
| Otu012 | Bacteria(100);"Thermotogae"(100);Thermotogae(100);Thermotogales(100);Thermotogaceae(100);unclassified(100); | RI(2t)downUni | 1686 |
| Otu012 | Bacteria(100);"Thermotogae"(100);Thermotogae(100);Thermotogales(100);Thermotogaceae(100);unclassified(100); | RI(2t)upUni | 14456 |
| Otu012 | Bacteria(100);"Thermotogae"(100);Thermotogae(100);Thermotogales(100);Thermotogaceae(100);unclassified(100); | RI(3t)downUni | 431 |
| Otu012 | Bacteria(100);"Thermotogae"(100);Thermotogae(100);Thermotogales(100);Thermotogaceae(100);unclassified(100); | RI(3t)upUni | 158 |
| Otu012 | Bacteria(100);"Thermotogae"(100);Thermotogae(100);Thermotogales(100);Thermotogaceae(100);unclassified(100); | RII(1t)downUni | 6 |
| Otu012 | Bacteria(100);"Thermotogae"(100);Thermotogae(100);Thermotogales(100);Thermotogaceae(100);unclassified(100); | RII(1t)upUni | 14 |
| Otu012 | Bacteria(100);"Thermotogae"(100);Thermotogae(100);Thermotogales(100);Thermotogaceae(100);unclassified(100); | RII(2t)downUni | 768 |
| Otu012 | Bacteria(100);"Thermotogae"(100);Thermotogae(100);Thermotogales(100);Thermotogaceae(100);unclassified(100); | RII(2t)upUni | 2140 |
| Otu012 | Bacteria(100);"Thermotogae"(100);Thermotogae(100);Thermotogales(100);Thermotogaceae(100);unclassified(100); | RII(3t)downUni | 2148 |
| Otu012 | Bacteria(100);"Thermotogae"(100);Thermotogae(100);Thermotogales(100);Thermotogaceae(100);unclassified(100); | RII(3t)upUni | 2609 |
| Otu012 | Bacteria(100);"Thermotogae"(100);Thermotogae(100);Thermotogales(100);Thermotogaceae(100);unclassified(100); | S1Uni | 5 |
| Otu012 | Bacteria(100);"Thermotogae"(100);Thermotogae(100);Thermotogales(100);Thermotogaceae(100);unclassified(100); | S2Uni | 4 |
| Otu013 | Bacteria(100);"Proteobacteria"(100);Deltaproteobacteria(100);Syntrophobacterales(100);Syntrophaceae(100);unclassified(100); | RI(1t)downUni | 918 |
| Otu013 | Bacteria(100);"Proteobacteria"(100);Deltaproteobacteria(100);Syntrophobacterales(100);Syntrophaceae(100);unclassified(100); | RI(1t)upUni | 271 |
| Otu013 | Bacteria(100);"Proteobacteria"(100);Deltaproteobacteria(100);Syntrophobacterales(100);Syntrophaceae(100);unclassified(100); | RI(2t)downUni | 320 |
| Otu013 | Bacteria(100);"Proteobacteria"(100);Deltaproteobacteria(100);Syntrophobacterales(100);Syntrophaceae(100);unclassified(100); | RI(2t)upUni | 1544 |
| Otu013 | Bacteria(100);"Proteobacteria"(100);Deltaproteobacteria(100);Syntrophobacterales(100);Syntrophaceae(100);unclassified(100); | RI(3t)downUni | 174 |
| Otu013 | Bacteria(100);"Proteobacteria"(100);Deltaproteobacteria(100);Syntrophobacterales(100);Syntrophaceae(100);unclassified(100); | RI(3t)upUni | 65 |
| Otu013 | Bacteria(100);"Proteobacteria"(100);Deltaproteobacteria(100);Syntrophobacterales(100);Syntrophaceae(100);unclassified(100); | RII(1t)downUni | 2807 |
| Otu013 | Bacteria(100);"Proteobacteria"(100);Deltaproteobacteria(100);Syntrophobacterales(100);Syntrophaceae(100);unclassified(100); | RII(1t)upUni | 745 |
| Otu013 | Bacteria(100);"Proteobacteria"(100);Deltaproteobacteria(100);Syntrophobacterales(100);Syntrophaceae(100);unclassified(100); | RII(2t)downUni | 273 |
| Otu013 | Bacteria(100);"Proteobacteria"(100);Deltaproteobacteria(100);Syntrophobacterales(100);Syntrophaceae(100);unclassified(100); | RII(2t)upUni | 887 |
| Otu013 | Bacteria(100);"Proteobacteria"(100);Deltaproteobacteria(100);Syntrophobacterales(100);Syntrophaceae(100);unclassified(100); | RII(3t)downUni | 171 |
| Otu013 | Bacteria(100);"Proteobacteria"(100);Deltaproteobacteria(100);Syntrophobacterales(100);Syntrophaceae(100);unclassified(100); | RII(3t)upUni | 49 |
| Otu013 | Bacteria(100);"Proteobacteria"(100);Deltaproteobacteria(100);Syntrophobacterales(100);Syntrophaceae(100);unclassified(100); | S1Uni | 15229 |
| Otu013 | Bacteria(100);"Proteobacteria"(100);Deltaproteobacteria(100);Syntrophobacterales(100);Syntrophaceae(100);unclassified(100); | S2Uni | 17049 |
| Otu014 | Bacteria(100);"Proteobacteria"(100);Betaproteobacteria(100);Burkholderiales(100);Comamonadaceae(100);unclassified(100); | RI(1t)downUni | 1669 |
| Otu014 | Bacteria(100);"Proteobacteria"(100);Betaproteobacteria(100);Burkholderiales(100);Comamonadaceae(100);unclassified(100); | RI(1t)upUni | 5023 |
| Otu014 | Bacteria(100);"Proteobacteria"(100);Betaproteobacteria(100);Burkholderiales(100);Comamonadaceae(100);unclassified(100); | RI(2t)downUni | 517 |
| Otu014 | Bacteria(100);"Proteobacteria"(100);Betaproteobacteria(100);Burkholderiales(100);Comamonadaceae(100);unclassified(100); | RI(2t)upUni | 3014 |
| Otu014 | Bacteria(100);"Proteobacteria"(100);Betaproteobacteria(100);Burkholderiales(100);Comamonadaceae(100);unclassified(100); | RI(3t)downUni | 102 |
| Otu014 | Bacteria(100);"Proteobacteria"(100);Betaproteobacteria(100);Burkholderiales(100);Comamonadaceae(100);unclassified(100); | RI(3t)upUni | 56 |
| Otu014 | Bacteria(100);"Proteobacteria"(100);Betaproteobacteria(100);Burkholderiales(100);Comamonadaceae(100);unclassified(100); | RII(1t)downUni | 2792 |
| Otu014 | Bacteria(100);"Proteobacteria"(100);Betaproteobacteria(100);Burkholderiales(100);Comamonadaceae(100);unclassified(100); | RII(1t)upUni | 10743 |
| Otu014 | Bacteria(100);"Proteobacteria"(100);Betaproteobacteria(100);Burkholderiales(100);Comamonadaceae(100);unclassified(100); | RII(2t)downUni | 433 |
| Otu014 | Bacteria(100);"Proteobacteria"(100);Betaproteobacteria(100);Burkholderiales(100);Comamonadaceae(100);unclassified(100); | RII(2t)upUni | 1839 |
| Otu014 | Bacteria(100);"Proteobacteria"(100);Betaproteobacteria(100);Burkholderiales(100);Comamonadaceae(100);unclassified(100); | RII(3t)downUni | 203 |
| Otu014 | Bacteria(100);"Proteobacteria"(100);Betaproteobacteria(100);Burkholderiales(100);Comamonadaceae(100);unclassified(100); | RII(3t)upUni | 708 |
| Otu014 | Bacteria(100);"Proteobacteria"(100);Betaproteobacteria(100);Burkholderiales(100);Comamonadaceae(100);unclassified(100); | S1Uni | 3456 |
| Otu014 | Bacteria(100);"Proteobacteria"(100);Betaproteobacteria(100);Burkholderiales(100);Comamonadaceae(100);unclassified(100); | S2Uni | 4047 |
| Otu015 | Bacteria(100);"Proteobacteria"(100);Gammaproteobacteria(100);Pseudomonadales(100);Moraxellaceae(100);Acinetobacter(100); | RI(1t)downUni | 859 |
| Otu015 | Bacteria(100);"Proteobacteria"(100);Gammaproteobacteria(100);Pseudomonadales(100);Moraxellaceae(100);Acinetobacter(100); | RI(1t)upUni | 4001 |
| Otu015 | Bacteria(100);"Proteobacteria"(100);Gammaproteobacteria(100);Pseudomonadales(100);Moraxellaceae(100);Acinetobacter(100); | RI(2t)downUni | 3317 |
| Otu015 | Bacteria(100);"Proteobacteria"(100);Gammaproteobacteria(100);Pseudomonadales(100);Moraxellaceae(100);Acinetobacter(100); | RI(2t)upUni | 25700 |
| Otu015 | Bacteria(100);"Proteobacteria"(100);Gammaproteobacteria(100);Pseudomonadales(100);Moraxellaceae(100);Acinetobacter(100); | RI(3t)downUni | 69 |
| Otu015 | Bacteria(100);"Proteobacteria"(100);Gammaproteobacteria(100);Pseudomonadales(100);Moraxellaceae(100);Acinetobacter(100); | RI(3t)upUni | 196 |
| Otu015 | Bacteria(100);"Proteobacteria"(100);Gammaproteobacteria(100);Pseudomonadales(100);Moraxellaceae(100);Acinetobacter(100); | RII(1t)downUni | 1691 |
| Otu015 | Bacteria(100);"Proteobacteria"(100);Gammaproteobacteria(100);Pseudomonadales(100);Moraxellaceae(100);Acinetobacter(100); | RII(1t)upUni | 3387 |
| Otu015 | Bacteria(100);"Proteobacteria"(100);Gammaproteobacteria(100);Pseudomonadales(100);Moraxellaceae(100);Acinetobacter(100); | RII(2t)downUni | 3813 |
| Otu015 | Bacteria(100);"Proteobacteria"(100);Gammaproteobacteria(100);Pseudomonadales(100);Moraxellaceae(100);Acinetobacter(100); | RII(2t)upUni | 16575 |
| Otu015 | Bacteria(100);"Proteobacteria"(100);Gammaproteobacteria(100);Pseudomonadales(100);Moraxellaceae(100);Acinetobacter(100); | RII(3t)downUni | 157 |
| Otu015 | Bacteria(100);"Proteobacteria"(100);Gammaproteobacteria(100);Pseudomonadales(100);Moraxellaceae(100);Acinetobacter(100); | RII(3t)upUni | 730 |
| Otu015 | Bacteria(100);"Proteobacteria"(100);Gammaproteobacteria(100);Pseudomonadales(100);Moraxellaceae(100);Acinetobacter(100); | S1Uni | 91 |
| Otu015 | Bacteria(100);"Proteobacteria"(100);Gammaproteobacteria(100);Pseudomonadales(100);Moraxellaceae(100);Acinetobacter(100); | S2Uni | 123 |
| Otu016 | Bacteria(100);"Synergistetes"(100);Synergistia(100);Synergistales(100);Synergistaceae(100);unclassified(100); | RI(1t)downUni | 949 |
| Otu016 | Bacteria(100);"Synergistetes"(100);Synergistia(100);Synergistales(100);Synergistaceae(100);unclassified(100); | RI(1t)upUni | 19158 |
| Otu016 | Bacteria(100);"Synergistetes"(100);Synergistia(100);Synergistales(100);Synergistaceae(100);unclassified(100); | RI(2t)downUni | 1584 |
| Otu016 | Bacteria(100);"Synergistetes"(100);Synergistia(100);Synergistales(100);Synergistaceae(100);unclassified(100); | RI(2t)upUni | 18646 |
| Otu016 | Bacteria(100);"Synergistetes"(100);Synergistia(100);Synergistales(100);Synergistaceae(100);unclassified(100); | RI(3t)downUni | 238 |
| Otu016 | Bacteria(100);"Synergistetes"(100);Synergistia(100);Synergistales(100);Synergistaceae(100);unclassified(100); | RI(3t)upUni | 834 |
| Otu016 | Bacteria(100);"Synergistetes"(100);Synergistia(100);Synergistales(100);Synergistaceae(100);unclassified(100); | RII(1t)downUni | 2263 |
| Otu016 | Bacteria(100);"Synergistetes"(100);Synergistia(100);Synergistales(100);Synergistaceae(100);unclassified(100); | RII(1t)upUni | 9920 |
| Otu016 | Bacteria(100);"Synergistetes"(100);Synergistia(100);Synergistales(100);Synergistaceae(100);unclassified(100); | RII(2t)downUni | 2385 |
| Otu016 | Bacteria(100);"Synergistetes"(100);Synergistia(100);Synergistales(100);Synergistaceae(100);unclassified(100); | RII(2t)upUni | 13235 |
| Otu016 | Bacteria(100);"Synergistetes"(100);Synergistia(100);Synergistales(100);Synergistaceae(100);unclassified(100); | RII(3t)downUni | 1651 |
| Otu016 | Bacteria(100);"Synergistetes"(100);Synergistia(100);Synergistales(100);Synergistaceae(100);unclassified(100); | RII(3t)upUni | 1672 |
| Otu016 | Bacteria(100);"Synergistetes"(100);Synergistia(100);Synergistales(100);Synergistaceae(100);unclassified(100); | S1Uni | 74 |
| Otu016 | Bacteria(100);"Synergistetes"(100);Synergistia(100);Synergistales(100);Synergistaceae(100);unclassified(100); | S2Uni | 33 |
| Otu017 | Bacteria(100);"Bacteroidetes"(100);"Bacteroidia"(100);"Bacteroidales"(100);"Porphyromonadaceae"(100);unclassified(100); | RI(1t)downUni | 639 |
| Otu017 | Bacteria(100);"Bacteroidetes"(100);"Bacteroidia"(100);"Bacteroidales"(100);"Porphyromonadaceae"(100);unclassified(100); | RI(1t)upUni | 1997 |
| Otu017 | Bacteria(100);"Bacteroidetes"(100);"Bacteroidia"(100);"Bacteroidales"(100);"Porphyromonadaceae"(100);unclassified(100); | RI(2t)downUni | 164 |
| Otu017 | Bacteria(100);"Bacteroidetes"(100);"Bacteroidia"(100);"Bacteroidales"(100);"Porphyromonadaceae"(100);unclassified(100); | RI(2t)upUni | 995 |
| Otu017 | Bacteria(100);"Bacteroidetes"(100);"Bacteroidia"(100);"Bacteroidales"(100);"Porphyromonadaceae"(100);unclassified(100); | RI(3t)downUni | 152 |
| Otu017 | Bacteria(100);"Bacteroidetes"(100);"Bacteroidia"(100);"Bacteroidales"(100);"Porphyromonadaceae"(100);unclassified(100); | RI(3t)upUni | 114 |
| Otu017 | Bacteria(100);"Bacteroidetes"(100);"Bacteroidia"(100);"Bacteroidales"(100);"Porphyromonadaceae"(100);unclassified(100); | RII(1t)downUni | 2250 |
| Otu017 | Bacteria(100);"Bacteroidetes"(100);"Bacteroidia"(100);"Bacteroidales"(100);"Porphyromonadaceae"(100);unclassified(100); | RII(1t)upUni | 1583 |
| Otu017 | Bacteria(100);"Bacteroidetes"(100);"Bacteroidia"(100);"Bacteroidales"(100);"Porphyromonadaceae"(100);unclassified(100); | RII(2t)downUni | 288 |
| Otu017 | Bacteria(100);"Bacteroidetes"(100);"Bacteroidia"(100);"Bacteroidales"(100);"Porphyromonadaceae"(100);unclassified(100); | RII(2t)upUni | 896 |
| Otu017 | Bacteria(100);"Bacteroidetes"(100);"Bacteroidia"(100);"Bacteroidales"(100);"Porphyromonadaceae"(100);unclassified(100); | RII(3t)downUni | 172 |
| Otu017 | Bacteria(100);"Bacteroidetes"(100);"Bacteroidia"(100);"Bacteroidales"(100);"Porphyromonadaceae"(100);unclassified(100); | RII(3t)upUni | 323 |
| Otu017 | Bacteria(100);"Bacteroidetes"(100);"Bacteroidia"(100);"Bacteroidales"(100);"Porphyromonadaceae"(100);unclassified(100); | S1Uni | 86 |
| Otu017 | Bacteria(100);"Bacteroidetes"(100);"Bacteroidia"(100);"Bacteroidales"(100);"Porphyromonadaceae"(100);unclassified(100); | S2Uni | 90 |
| Otu018 | Bacteria(100);"Proteobacteria"(100);Betaproteobacteria(100);Hydrogenophilales(100);Hydrogenophilaceae(100);Thiobacillus(100); | RI(1t)downUni | 1380 |
| Otu018 | Bacteria(100);"Proteobacteria"(100);Betaproteobacteria(100);Hydrogenophilales(100);Hydrogenophilaceae(100);Thiobacillus(100); | RI(1t)upUni | 123 |
| Otu018 | Bacteria(100);"Proteobacteria"(100);Betaproteobacteria(100);Hydrogenophilales(100);Hydrogenophilaceae(100);Thiobacillus(100); | RI(2t)downUni | 381 |
| Otu018 | Bacteria(100);"Proteobacteria"(100);Betaproteobacteria(100);Hydrogenophilales(100);Hydrogenophilaceae(100);Thiobacillus(100); | RI(2t)upUni | 1058 |
| Otu018 | Bacteria(100);"Proteobacteria"(100);Betaproteobacteria(100);Hydrogenophilales(100);Hydrogenophilaceae(100);Thiobacillus(100); | RI(3t)downUni | 131 |
| Otu018 | Bacteria(100);"Proteobacteria"(100);Betaproteobacteria(100);Hydrogenophilales(100);Hydrogenophilaceae(100);Thiobacillus(100); | RI(3t)upUni | 36 |
| Otu018 | Bacteria(100);"Proteobacteria"(100);Betaproteobacteria(100);Hydrogenophilales(100);Hydrogenophilaceae(100);Thiobacillus(100); | RII(1t)downUni | 5029 |
| Otu018 | Bacteria(100);"Proteobacteria"(100);Betaproteobacteria(100);Hydrogenophilales(100);Hydrogenophilaceae(100);Thiobacillus(100); | RII(1t)upUni | 796 |
| Otu018 | Bacteria(100);"Proteobacteria"(100);Betaproteobacteria(100);Hydrogenophilales(100);Hydrogenophilaceae(100);Thiobacillus(100); | RII(2t)downUni | 424 |
| Otu018 | Bacteria(100);"Proteobacteria"(100);Betaproteobacteria(100);Hydrogenophilales(100);Hydrogenophilaceae(100);Thiobacillus(100); | RII(2t)upUni | 1284 |
| Otu018 | Bacteria(100);"Proteobacteria"(100);Betaproteobacteria(100);Hydrogenophilales(100);Hydrogenophilaceae(100);Thiobacillus(100); | RII(3t)downUni | 349 |
| Otu018 | Bacteria(100);"Proteobacteria"(100);Betaproteobacteria(100);Hydrogenophilales(100);Hydrogenophilaceae(100);Thiobacillus(100); | RII(3t)upUni | 93 |
| Otu018 | Bacteria(100);"Proteobacteria"(100);Betaproteobacteria(100);Hydrogenophilales(100);Hydrogenophilaceae(100);Thiobacillus(100); | S1Uni | 11513 |
| Otu018 | Bacteria(100);"Proteobacteria"(100);Betaproteobacteria(100);Hydrogenophilales(100);Hydrogenophilaceae(100);Thiobacillus(100); | S2Uni | 15977 |
| Otu019 | Bacteria(100);"Bacteroidetes"(100);"Bacteroidia"(100);"Bacteroidales"(100);Bacteroidaceae(100);Bacteroides(100); | RI(1t)downUni | 2863 |
| Otu019 | Bacteria(100);"Bacteroidetes"(100);"Bacteroidia"(100);"Bacteroidales"(100);Bacteroidaceae(100);Bacteroides(100); | RI(1t)upUni | 17457 |
| Otu019 | Bacteria(100);"Bacteroidetes"(100);"Bacteroidia"(100);"Bacteroidales"(100);Bacteroidaceae(100);Bacteroides(100); | RI(2t)downUni | 235 |
| Otu019 | Bacteria(100);"Bacteroidetes"(100);"Bacteroidia"(100);"Bacteroidales"(100);Bacteroidaceae(100);Bacteroides(100); | RI(2t)upUni | 1265 |
| Otu019 | Bacteria(100);"Bacteroidetes"(100);"Bacteroidia"(100);"Bacteroidales"(100);Bacteroidaceae(100);Bacteroides(100); | RI(3t)downUni | 359 |
| Otu019 | Bacteria(100);"Bacteroidetes"(100);"Bacteroidia"(100);"Bacteroidales"(100);Bacteroidaceae(100);Bacteroides(100); | RI(3t)upUni | 397 |
| Otu019 | Bacteria(100);"Bacteroidetes"(100);"Bacteroidia"(100);"Bacteroidales"(100);Bacteroidaceae(100);Bacteroides(100); | RII(1t)downUni | 8097 |
| Otu019 | Bacteria(100);"Bacteroidetes"(100);"Bacteroidia"(100);"Bacteroidales"(100);Bacteroidaceae(100);Bacteroides(100); | RII(1t)upUni | 10300 |
| Otu019 | Bacteria(100);"Bacteroidetes"(100);"Bacteroidia"(100);"Bacteroidales"(100);Bacteroidaceae(100);Bacteroides(100); | RII(2t)downUni | 173 |
| Otu019 | Bacteria(100);"Bacteroidetes"(100);"Bacteroidia"(100);"Bacteroidales"(100);Bacteroidaceae(100);Bacteroides(100); | RII(2t)upUni | 749 |
| Otu019 | Bacteria(100);"Bacteroidetes"(100);"Bacteroidia"(100);"Bacteroidales"(100);Bacteroidaceae(100);Bacteroides(100); | RII(3t)downUni | 679 |
| Otu019 | Bacteria(100);"Bacteroidetes"(100);"Bacteroidia"(100);"Bacteroidales"(100);Bacteroidaceae(100);Bacteroides(100); | RII(3t)upUni | 5022 |
| Otu019 | Bacteria(100);"Bacteroidetes"(100);"Bacteroidia"(100);"Bacteroidales"(100);Bacteroidaceae(100);Bacteroides(100); | S1Uni | 38 |
| Otu019 | Bacteria(100);"Bacteroidetes"(100);"Bacteroidia"(100);"Bacteroidales"(100);Bacteroidaceae(100);Bacteroides(100); | S2Uni | 18 |
| Otu020 | Bacteria(100);"Proteobacteria"(100);Betaproteobacteria(100);unclassified(100);unclassified(100);unclassified(100); | RI(1t)downUni | 330 |
| Otu020 | Bacteria(100);"Proteobacteria"(100);Betaproteobacteria(100);unclassified(100);unclassified(100);unclassified(100); | RI(1t)upUni | 509 |
| Otu020 | Bacteria(100);"Proteobacteria"(100);Betaproteobacteria(100);unclassified(100);unclassified(100);unclassified(100); | RI(2t)downUni | 72 |
| Otu020 | Bacteria(100);"Proteobacteria"(100);Betaproteobacteria(100);unclassified(100);unclassified(100);unclassified(100); | RI(2t)upUni | 346 |
| Otu020 | Bacteria(100);"Proteobacteria"(100);Betaproteobacteria(100);unclassified(100);unclassified(100);unclassified(100); | RI(3t)downUni | 23 |
| Otu020 | Bacteria(100);"Proteobacteria"(100);Betaproteobacteria(100);unclassified(100);unclassified(100);unclassified(100); | RI(3t)upUni | 13 |
| Otu020 | Bacteria(100);"Proteobacteria"(100);Betaproteobacteria(100);unclassified(100);unclassified(100);unclassified(100); | RII(1t)downUni | 1265 |
| Otu020 | Bacteria(100);"Proteobacteria"(100);Betaproteobacteria(100);unclassified(100);unclassified(100);unclassified(100); | RII(1t)upUni | 353 |
| Otu020 | Bacteria(100);"Proteobacteria"(100);Betaproteobacteria(100);unclassified(100);unclassified(100);unclassified(100); | RII(2t)downUni | 100 |
| Otu020 | Bacteria(100);"Proteobacteria"(100);Betaproteobacteria(100);unclassified(100);unclassified(100);unclassified(100); | RII(2t)upUni | 269 |
| Otu020 | Bacteria(100);"Proteobacteria"(100);Betaproteobacteria(100);unclassified(100);unclassified(100);unclassified(100); | RII(3t)downUni | 79 |
| Otu020 | Bacteria(100);"Proteobacteria"(100);Betaproteobacteria(100);unclassified(100);unclassified(100);unclassified(100); | RII(3t)upUni | 63 |
| Otu020 | Bacteria(100);"Proteobacteria"(100);Betaproteobacteria(100);unclassified(100);unclassified(100);unclassified(100); | S1Uni | 5103 |
| Otu020 | Bacteria(100);"Proteobacteria"(100);Betaproteobacteria(100);unclassified(100);unclassified(100);unclassified(100); | S2Uni | 6502 |
| Otu022 | Bacteria(100);"Proteobacteria"(100);Epsilonproteobacteria(100);Campylobacterales(100);Campylobacteraceae(100);Arcobacter(100); | RI(1t)downUni | 773 |
| Otu022 | Bacteria(100);"Proteobacteria"(100);Epsilonproteobacteria(100);Campylobacterales(100);Campylobacteraceae(100);Arcobacter(100); | RI(1t)upUni | 5347 |
| Otu022 | Bacteria(100);"Proteobacteria"(100);Epsilonproteobacteria(100);Campylobacterales(100);Campylobacteraceae(100);Arcobacter(100); | RI(2t)downUni | 1257 |
| Otu022 | Bacteria(100);"Proteobacteria"(100);Epsilonproteobacteria(100);Campylobacterales(100);Campylobacteraceae(100);Arcobacter(100); | RI(2t)upUni | 7456 |
| Otu022 | Bacteria(100);"Proteobacteria"(100);Epsilonproteobacteria(100);Campylobacterales(100);Campylobacteraceae(100);Arcobacter(100); | RI(3t)downUni | 145 |
| Otu022 | Bacteria(100);"Proteobacteria"(100);Epsilonproteobacteria(100);Campylobacterales(100);Campylobacteraceae(100);Arcobacter(100); | RI(3t)upUni | 150 |
| Otu022 | Bacteria(100);"Proteobacteria"(100);Epsilonproteobacteria(100);Campylobacterales(100);Campylobacteraceae(100);Arcobacter(100); | RII(1t)downUni | 1251 |
| Otu022 | Bacteria(100);"Proteobacteria"(100);Epsilonproteobacteria(100);Campylobacterales(100);Campylobacteraceae(100);Arcobacter(100); | RII(1t)upUni | 6742 |
| Otu022 | Bacteria(100);"Proteobacteria"(100);Epsilonproteobacteria(100);Campylobacterales(100);Campylobacteraceae(100);Arcobacter(100); | RII(2t)downUni | 1661 |
| Otu022 | Bacteria(100);"Proteobacteria"(100);Epsilonproteobacteria(100);Campylobacterales(100);Campylobacteraceae(100);Arcobacter(100); | RII(2t)upUni | 8624 |
| Otu022 | Bacteria(100);"Proteobacteria"(100);Epsilonproteobacteria(100);Campylobacterales(100);Campylobacteraceae(100);Arcobacter(100); | RII(3t)downUni | 578 |
| Otu022 | Bacteria(100);"Proteobacteria"(100);Epsilonproteobacteria(100);Campylobacterales(100);Campylobacteraceae(100);Arcobacter(100); | RII(3t)upUni | 5942 |
| Otu022 | Bacteria(100);"Proteobacteria"(100);Epsilonproteobacteria(100);Campylobacterales(100);Campylobacteraceae(100);Arcobacter(100); | S1Uni | 470 |
| Otu022 | Bacteria(100);"Proteobacteria"(100);Epsilonproteobacteria(100);Campylobacterales(100);Campylobacteraceae(100);Arcobacter(100); | S2Uni | 574 |
| Otu023 | Bacteria(100);Firmicutes(100);Clostridia(100);Clostridiales(100);Ruminococcaceae(100);Saccharofermentans(100); | RI(1t)downUni | 242 |
| Otu023 | Bacteria(100);Firmicutes(100);Clostridia(100);Clostridiales(100);Ruminococcaceae(100);Saccharofermentans(100); | RI(1t)upUni | 72 |
| Otu023 | Bacteria(100);Firmicutes(100);Clostridia(100);Clostridiales(100);Ruminococcaceae(100);Saccharofermentans(100); | RI(2t)downUni | 1238 |
| Otu023 | Bacteria(100);Firmicutes(100);Clostridia(100);Clostridiales(100);Ruminococcaceae(100);Saccharofermentans(100); | RI(2t)upUni | 7072 |
| Otu023 | Bacteria(100);Firmicutes(100);Clostridia(100);Clostridiales(100);Ruminococcaceae(100);Saccharofermentans(100); | RI(3t)downUni | 1176 |
| Otu023 | Bacteria(100);Firmicutes(100);Clostridia(100);Clostridiales(100);Ruminococcaceae(100);Saccharofermentans(100); | RI(3t)upUni | 493 |
| Otu023 | Bacteria(100);Firmicutes(100);Clostridia(100);Clostridiales(100);Ruminococcaceae(100);Saccharofermentans(100); | RII(1t)downUni | 607 |
| Otu023 | Bacteria(100);Firmicutes(100);Clostridia(100);Clostridiales(100);Ruminococcaceae(100);Saccharofermentans(100); | RII(1t)upUni | 384 |
| Otu023 | Bacteria(100);Firmicutes(100);Clostridia(100);Clostridiales(100);Ruminococcaceae(100);Saccharofermentans(100); | RII(2t)downUni | 1341 |
| Otu023 | Bacteria(100);Firmicutes(100);Clostridia(100);Clostridiales(100);Ruminococcaceae(100);Saccharofermentans(100); | RII(2t)upUni | 5273 |
| Otu023 | Bacteria(100);Firmicutes(100);Clostridia(100);Clostridiales(100);Ruminococcaceae(100);Saccharofermentans(100); | RII(3t)downUni | 2952 |
| Otu023 | Bacteria(100);Firmicutes(100);Clostridia(100);Clostridiales(100);Ruminococcaceae(100);Saccharofermentans(100); | RII(3t)upUni | 1568 |
| Otu023 | Bacteria(100);Firmicutes(100);Clostridia(100);Clostridiales(100);Ruminococcaceae(100);Saccharofermentans(100); | S1Uni | 1109 |
| Otu023 | Bacteria(100);Firmicutes(100);Clostridia(100);Clostridiales(100);Ruminococcaceae(100);Saccharofermentans(100); | S2Uni | 1419 |
| Otu024 | Bacteria(100);"Proteobacteria"(100);Deltaproteobacteria(100);Deltaproteobacteria_order_incertae_sedis(100);Syntrophorhabdaceae(100);Syntrophorhabdus(100); | RI(1t)downUni | 249 |
| Otu024 | Bacteria(100);"Proteobacteria"(100);Deltaproteobacteria(100);Deltaproteobacteria_order_incertae_sedis(100);Syntrophorhabdaceae(100);Syntrophorhabdus(100); | RI(1t)upUni | 110 |
| Otu024 | Bacteria(100);"Proteobacteria"(100);Deltaproteobacteria(100);Deltaproteobacteria_order_incertae_sedis(100);Syntrophorhabdaceae(100);Syntrophorhabdus(100); | RI(2t)downUni | 61 |
| Otu024 | Bacteria(100);"Proteobacteria"(100);Deltaproteobacteria(100);Deltaproteobacteria_order_incertae_sedis(100);Syntrophorhabdaceae(100);Syntrophorhabdus(100); | RI(2t)upUni | 327 |
| Otu024 | Bacteria(100);"Proteobacteria"(100);Deltaproteobacteria(100);Deltaproteobacteria_order_incertae_sedis(100);Syntrophorhabdaceae(100);Syntrophorhabdus(100); | RI(3t)downUni | 31 |
| Otu024 | Bacteria(100);"Proteobacteria"(100);Deltaproteobacteria(100);Deltaproteobacteria_order_incertae_sedis(100);Syntrophorhabdaceae(100);Syntrophorhabdus(100); | RI(3t)upUni | 15 |
| Otu024 | Bacteria(100);"Proteobacteria"(100);Deltaproteobacteria(100);Deltaproteobacteria_order_incertae_sedis(100);Syntrophorhabdaceae(100);Syntrophorhabdus(100); | RII(1t)downUni | 654 |
| Otu024 | Bacteria(100);"Proteobacteria"(100);Deltaproteobacteria(100);Deltaproteobacteria_order_incertae_sedis(100);Syntrophorhabdaceae(100);Syntrophorhabdus(100); | RII(1t)upUni | 230 |
| Otu024 | Bacteria(100);"Proteobacteria"(100);Deltaproteobacteria(100);Deltaproteobacteria_order_incertae_sedis(100);Syntrophorhabdaceae(100);Syntrophorhabdus(100); | RII(2t)downUni | 81 |
| Otu024 | Bacteria(100);"Proteobacteria"(100);Deltaproteobacteria(100);Deltaproteobacteria_order_incertae_sedis(100);Syntrophorhabdaceae(100);Syntrophorhabdus(100); | RII(2t)upUni | 321 |
| Otu024 | Bacteria(100);"Proteobacteria"(100);Deltaproteobacteria(100);Deltaproteobacteria_order_incertae_sedis(100);Syntrophorhabdaceae(100);Syntrophorhabdus(100); | RII(3t)downUni | 57 |
| Otu024 | Bacteria(100);"Proteobacteria"(100);Deltaproteobacteria(100);Deltaproteobacteria_order_incertae_sedis(100);Syntrophorhabdaceae(100);Syntrophorhabdus(100); | RII(3t)upUni | 22 |
| Otu024 | Bacteria(100);"Proteobacteria"(100);Deltaproteobacteria(100);Deltaproteobacteria_order_incertae_sedis(100);Syntrophorhabdaceae(100);Syntrophorhabdus(100); | S1Uni | 3901 |
| Otu024 | Bacteria(100);"Proteobacteria"(100);Deltaproteobacteria(100);Deltaproteobacteria_order_incertae_sedis(100);Syntrophorhabdaceae(100);Syntrophorhabdus(100); | S2Uni | 4666 |
| Otu025 | Bacteria(100);"Bacteroidetes"(100);"Bacteroidia"(100);"Bacteroidales"(100);unclassified(100);unclassified(100); | RI(1t)downUni | 732 |
| Otu025 | Bacteria(100);"Bacteroidetes"(100);"Bacteroidia"(100);"Bacteroidales"(100);unclassified(100);unclassified(100); | RI(1t)upUni | 916 |
| Otu025 | Bacteria(100);"Bacteroidetes"(100);"Bacteroidia"(100);"Bacteroidales"(100);unclassified(100);unclassified(100); | RI(2t)downUni | 150 |
| Otu025 | Bacteria(100);"Bacteroidetes"(100);"Bacteroidia"(100);"Bacteroidales"(100);unclassified(100);unclassified(100); | RI(2t)upUni | 791 |
| Otu025 | Bacteria(100);"Bacteroidetes"(100);"Bacteroidia"(100);"Bacteroidales"(100);unclassified(100);unclassified(100); | RI(3t)downUni | 317 |
| Otu025 | Bacteria(100);"Bacteroidetes"(100);"Bacteroidia"(100);"Bacteroidales"(100);unclassified(100);unclassified(100); | RI(3t)upUni | 161 |
| Otu025 | Bacteria(100);"Bacteroidetes"(100);"Bacteroidia"(100);"Bacteroidales"(100);unclassified(100);unclassified(100); | RII(1t)downUni | 1857 |
| Otu025 | Bacteria(100);"Bacteroidetes"(100);"Bacteroidia"(100);"Bacteroidales"(100);unclassified(100);unclassified(100); | RII(1t)upUni | 1025 |
| Otu025 | Bacteria(100);"Bacteroidetes"(100);"Bacteroidia"(100);"Bacteroidales"(100);unclassified(100);unclassified(100); | RII(2t)downUni | 117 |
| Otu025 | Bacteria(100);"Bacteroidetes"(100);"Bacteroidia"(100);"Bacteroidales"(100);unclassified(100);unclassified(100); | RII(2t)upUni | 309 |
| Otu025 | Bacteria(100);"Bacteroidetes"(100);"Bacteroidia"(100);"Bacteroidales"(100);unclassified(100);unclassified(100); | RII(3t)downUni | 771 |
| Otu025 | Bacteria(100);"Bacteroidetes"(100);"Bacteroidia"(100);"Bacteroidales"(100);unclassified(100);unclassified(100); | RII(3t)upUni | 2035 |
| Otu025 | Bacteria(100);"Bacteroidetes"(100);"Bacteroidia"(100);"Bacteroidales"(100);unclassified(100);unclassified(100); | S1Uni | 2 |
| Otu025 | Bacteria(100);"Bacteroidetes"(100);"Bacteroidia"(100);"Bacteroidales"(100);unclassified(100);unclassified(100); | S2Uni | 4 |
| Otu026 | Bacteria(100);"Proteobacteria"(100);Betaproteobacteria(100);Rhodocyclales(100);Rhodocyclaceae(100);unclassified(100); | RI(1t)downUni | 719 |
| Otu026 | Bacteria(100);"Proteobacteria"(100);Betaproteobacteria(100);Rhodocyclales(100);Rhodocyclaceae(100);unclassified(100); | RI(1t)upUni | 2369 |
| Otu026 | Bacteria(100);"Proteobacteria"(100);Betaproteobacteria(100);Rhodocyclales(100);Rhodocyclaceae(100);unclassified(100); | RI(2t)downUni | 62 |
| Otu026 | Bacteria(100);"Proteobacteria"(100);Betaproteobacteria(100);Rhodocyclales(100);Rhodocyclaceae(100);unclassified(100); | RI(2t)upUni | 373 |
| Otu026 | Bacteria(100);"Proteobacteria"(100);Betaproteobacteria(100);Rhodocyclales(100);Rhodocyclaceae(100);unclassified(100); | RI(3t)downUni | 14 |
| Otu026 | Bacteria(100);"Proteobacteria"(100);Betaproteobacteria(100);Rhodocyclales(100);Rhodocyclaceae(100);unclassified(100); | RI(3t)upUni | 44 |
| Otu026 | Bacteria(100);"Proteobacteria"(100);Betaproteobacteria(100);Rhodocyclales(100);Rhodocyclaceae(100);unclassified(100); | RII(1t)downUni | 2316 |
| Otu026 | Bacteria(100);"Proteobacteria"(100);Betaproteobacteria(100);Rhodocyclales(100);Rhodocyclaceae(100);unclassified(100); | RII(1t)upUni | 2120 |
| Otu026 | Bacteria(100);"Proteobacteria"(100);Betaproteobacteria(100);Rhodocyclales(100);Rhodocyclaceae(100);unclassified(100); | RII(2t)downUni | 71 |
| Otu026 | Bacteria(100);"Proteobacteria"(100);Betaproteobacteria(100);Rhodocyclales(100);Rhodocyclaceae(100);unclassified(100); | RII(2t)upUni | 308 |
| Otu026 | Bacteria(100);"Proteobacteria"(100);Betaproteobacteria(100);Rhodocyclales(100);Rhodocyclaceae(100);unclassified(100); | RII(3t)downUni | 36 |
| Otu026 | Bacteria(100);"Proteobacteria"(100);Betaproteobacteria(100);Rhodocyclales(100);Rhodocyclaceae(100);unclassified(100); | RII(3t)upUni | 38 |
| Otu026 | Bacteria(100);"Proteobacteria"(100);Betaproteobacteria(100);Rhodocyclales(100);Rhodocyclaceae(100);unclassified(100); | S1Uni | 2718 |
| Otu026 | Bacteria(100);"Proteobacteria"(100);Betaproteobacteria(100);Rhodocyclales(100);Rhodocyclaceae(100);unclassified(100); | S2Uni | 4126 |
| Otu027 | Bacteria(100);"Proteobacteria"(100);Gammaproteobacteria(100);"Enterobacteriales"(100);Enterobacteriaceae(100);unclassified(100); | RI(1t)downUni | 1013 |
| Otu027 | Bacteria(100);"Proteobacteria"(100);Gammaproteobacteria(100);"Enterobacteriales"(100);Enterobacteriaceae(100);unclassified(100); | RI(1t)upUni | 5657 |
| Otu027 | Bacteria(100);"Proteobacteria"(100);Gammaproteobacteria(100);"Enterobacteriales"(100);Enterobacteriaceae(100);unclassified(100); | RI(2t)downUni | 65 |
| Otu027 | Bacteria(100);"Proteobacteria"(100);Gammaproteobacteria(100);"Enterobacteriales"(100);Enterobacteriaceae(100);unclassified(100); | RI(2t)upUni | 797 |
| Otu027 | Bacteria(100);"Proteobacteria"(100);Gammaproteobacteria(100);"Enterobacteriales"(100);Enterobacteriaceae(100);unclassified(100); | RI(3t)downUni | 66 |
| Otu027 | Bacteria(100);"Proteobacteria"(100);Gammaproteobacteria(100);"Enterobacteriales"(100);Enterobacteriaceae(100);unclassified(100); | RI(3t)upUni | 76 |
| Otu027 | Bacteria(100);"Proteobacteria"(100);Gammaproteobacteria(100);"Enterobacteriales"(100);Enterobacteriaceae(100);unclassified(100); | RII(1t)downUni | 2816 |
| Otu027 | Bacteria(100);"Proteobacteria"(100);Gammaproteobacteria(100);"Enterobacteriales"(100);Enterobacteriaceae(100);unclassified(100); | RII(1t)upUni | 7818 |
| Otu027 | Bacteria(100);"Proteobacteria"(100);Gammaproteobacteria(100);"Enterobacteriales"(100);Enterobacteriaceae(100);unclassified(100); | RII(2t)downUni | 63 |
| Otu027 | Bacteria(100);"Proteobacteria"(100);Gammaproteobacteria(100);"Enterobacteriales"(100);Enterobacteriaceae(100);unclassified(100); | RII(2t)upUni | 306 |
| Otu027 | Bacteria(100);"Proteobacteria"(100);Gammaproteobacteria(100);"Enterobacteriales"(100);Enterobacteriaceae(100);unclassified(100); | RII(3t)downUni | 106 |
| Otu027 | Bacteria(100);"Proteobacteria"(100);Gammaproteobacteria(100);"Enterobacteriales"(100);Enterobacteriaceae(100);unclassified(100); | RII(3t)upUni | 329 |
| Otu027 | Bacteria(100);"Proteobacteria"(100);Gammaproteobacteria(100);"Enterobacteriales"(100);Enterobacteriaceae(100);unclassified(100); | S1Uni | 325 |
| Otu027 | Bacteria(100);"Proteobacteria"(100);Gammaproteobacteria(100);"Enterobacteriales"(100);Enterobacteriaceae(100);unclassified(100); | S2Uni | 434 |
| Otu028 | Bacteria(100);Firmicutes(100);Clostridia(100);unclassified(100);unclassified(100);unclassified(100); | RI(1t)downUni | 11515 |
| Otu028 | Bacteria(100);Firmicutes(100);Clostridia(100);unclassified(100);unclassified(100);unclassified(100); | RI(1t)upUni | 4447 |
| Otu028 | Bacteria(100);Firmicutes(100);Clostridia(100);unclassified(100);unclassified(100);unclassified(100); | RI(2t)downUni | 181 |
| Otu028 | Bacteria(100);Firmicutes(100);Clostridia(100);unclassified(100);unclassified(100);unclassified(100); | RI(2t)upUni | 995 |
| Otu028 | Bacteria(100);Firmicutes(100);Clostridia(100);unclassified(100);unclassified(100);unclassified(100); | RI(3t)downUni | 259 |
| Otu028 | Bacteria(100);Firmicutes(100);Clostridia(100);unclassified(100);unclassified(100);unclassified(100); | RI(3t)upUni | 808 |
| Otu028 | Bacteria(100);Firmicutes(100);Clostridia(100);unclassified(100);unclassified(100);unclassified(100); | RII(1t)downUni | 19878 |
| Otu028 | Bacteria(100);Firmicutes(100);Clostridia(100);unclassified(100);unclassified(100);unclassified(100); | RII(1t)upUni | 13939 |
| Otu028 | Bacteria(100);Firmicutes(100);Clostridia(100);unclassified(100);unclassified(100);unclassified(100); | RII(2t)downUni | 936 |
| Otu028 | Bacteria(100);Firmicutes(100);Clostridia(100);unclassified(100);unclassified(100);unclassified(100); | RII(2t)upUni | 3532 |
| Otu028 | Bacteria(100);Firmicutes(100);Clostridia(100);unclassified(100);unclassified(100);unclassified(100); | RII(3t)downUni | 272 |
| Otu028 | Bacteria(100);Firmicutes(100);Clostridia(100);unclassified(100);unclassified(100);unclassified(100); | RII(3t)upUni | 569 |
| Otu028 | Bacteria(100);Firmicutes(100);Clostridia(100);unclassified(100);unclassified(100);unclassified(100); | S1Uni | 330 |
| Otu028 | Bacteria(100);Firmicutes(100);Clostridia(100);unclassified(100);unclassified(100);unclassified(100); | S2Uni | 311 |
| Otu029 | Bacteria(100);"Synergistetes"(100);Synergistia(100);Synergistales(100);Synergistaceae(100);Thermovirga(100); | RI(1t)downUni | 4 |
| Otu029 | Bacteria(100);"Synergistetes"(100);Synergistia(100);Synergistales(100);Synergistaceae(100);Thermovirga(100); | RI(1t)upUni | 3 |
| Otu029 | Bacteria(100);"Synergistetes"(100);Synergistia(100);Synergistales(100);Synergistaceae(100);Thermovirga(100); | RI(2t)downUni | 370 |
| Otu029 | Bacteria(100);"Synergistetes"(100);Synergistia(100);Synergistales(100);Synergistaceae(100);Thermovirga(100); | RI(2t)upUni | 7211 |
| Otu029 | Bacteria(100);"Synergistetes"(100);Synergistia(100);Synergistales(100);Synergistaceae(100);Thermovirga(100); | RI(3t)downUni | 809 |
| Otu029 | Bacteria(100);"Synergistetes"(100);Synergistia(100);Synergistales(100);Synergistaceae(100);Thermovirga(100); | RI(3t)upUni | 1599 |
| Otu029 | Bacteria(100);"Synergistetes"(100);Synergistia(100);Synergistales(100);Synergistaceae(100);Thermovirga(100); | RII(1t)downUni | 7 |
| Otu029 | Bacteria(100);"Synergistetes"(100);Synergistia(100);Synergistales(100);Synergistaceae(100);Thermovirga(100); | RII(1t)upUni | 10 |
| Otu029 | Bacteria(100);"Synergistetes"(100);Synergistia(100);Synergistales(100);Synergistaceae(100);Thermovirga(100); | RII(2t)downUni | 319 |
| Otu029 | Bacteria(100);"Synergistetes"(100);Synergistia(100);Synergistales(100);Synergistaceae(100);Thermovirga(100); | RII(2t)upUni | 768 |
| Otu029 | Bacteria(100);"Synergistetes"(100);Synergistia(100);Synergistales(100);Synergistaceae(100);Thermovirga(100); | RII(3t)downUni | 958 |
| Otu029 | Bacteria(100);"Synergistetes"(100);Synergistia(100);Synergistales(100);Synergistaceae(100);Thermovirga(100); | RII(3t)upUni | 1058 |
| Otu029 | Bacteria(100);"Synergistetes"(100);Synergistia(100);Synergistales(100);Synergistaceae(100);Thermovirga(100); | S1Uni | 2 |
| Otu029 | Bacteria(100);"Synergistetes"(100);Synergistia(100);Synergistales(100);Synergistaceae(100);Thermovirga(100); | S2Uni | 9 |
| Otu030 | Bacteria(100);Firmicutes(100);Clostridia(100);Clostridiales(100);Clostridiales_Incertae_Sedis_XIII(100);Anaerovorax(100); | RI(1t)downUni | 241 |
| Otu030 | Bacteria(100);Firmicutes(100);Clostridia(100);Clostridiales(100);Clostridiales_Incertae_Sedis_XIII(100);Anaerovorax(100); | RI(1t)upUni | 329 |
| Otu030 | Bacteria(100);Firmicutes(100);Clostridia(100);Clostridiales(100);Clostridiales_Incertae_Sedis_XIII(100);Anaerovorax(100); | RI(2t)downUni | 1042 |
| Otu030 | Bacteria(100);Firmicutes(100);Clostridia(100);Clostridiales(100);Clostridiales_Incertae_Sedis_XIII(100);Anaerovorax(100); | RI(2t)upUni | 9836 |
| Otu030 | Bacteria(100);Firmicutes(100);Clostridia(100);Clostridiales(100);Clostridiales_Incertae_Sedis_XIII(100);Anaerovorax(100); | RI(3t)downUni | 424 |
| Otu030 | Bacteria(100);Firmicutes(100);Clostridia(100);Clostridiales(100);Clostridiales_Incertae_Sedis_XIII(100);Anaerovorax(100); | RI(3t)upUni | 228 |
| Otu030 | Bacteria(100);Firmicutes(100);Clostridia(100);Clostridiales(100);Clostridiales_Incertae_Sedis_XIII(100);Anaerovorax(100); | RII(1t)downUni | 1010 |
| Otu030 | Bacteria(100);Firmicutes(100);Clostridia(100);Clostridiales(100);Clostridiales_Incertae_Sedis_XIII(100);Anaerovorax(100); | RII(1t)upUni | 523 |
| Otu030 | Bacteria(100);Firmicutes(100);Clostridia(100);Clostridiales(100);Clostridiales_Incertae_Sedis_XIII(100);Anaerovorax(100); | RII(2t)downUni | 673 |
| Otu030 | Bacteria(100);Firmicutes(100);Clostridia(100);Clostridiales(100);Clostridiales_Incertae_Sedis_XIII(100);Anaerovorax(100); | RII(2t)upUni | 3020 |
| Otu030 | Bacteria(100);Firmicutes(100);Clostridia(100);Clostridiales(100);Clostridiales_Incertae_Sedis_XIII(100);Anaerovorax(100); | RII(3t)downUni | 851 |
| Otu030 | Bacteria(100);Firmicutes(100);Clostridia(100);Clostridiales(100);Clostridiales_Incertae_Sedis_XIII(100);Anaerovorax(100); | RII(3t)upUni | 604 |
| Otu030 | Bacteria(100);Firmicutes(100);Clostridia(100);Clostridiales(100);Clostridiales_Incertae_Sedis_XIII(100);Anaerovorax(100); | S1Uni | 89 |
| Otu030 | Bacteria(100);Firmicutes(100);Clostridia(100);Clostridiales(100);Clostridiales_Incertae_Sedis_XIII(100);Anaerovorax(100); | S2Uni | 164 |
| Otu031 | Bacteria(100);"Armatimonadetes"(100);Armatimonadetes_gp2_class_incertae_sedis(100);Armatimonadetes_gp2_order_incetae_sedis(100);Armatimonadetes_gp2_family_incetae_sedis(100);Armatimonadetes_gp2(100); | RI(1t)downUni | 174 |
| Otu031 | Bacteria(100);"Armatimonadetes"(100);Armatimonadetes_gp2_class_incertae_sedis(100);Armatimonadetes_gp2_order_incetae_sedis(100);Armatimonadetes_gp2_family_incetae_sedis(100);Armatimonadetes_gp2(100); | RI(1t)upUni | 110 |
| Otu031 | Bacteria(100);"Armatimonadetes"(100);Armatimonadetes_gp2_class_incertae_sedis(100);Armatimonadetes_gp2_order_incetae_sedis(100);Armatimonadetes_gp2_family_incetae_sedis(100);Armatimonadetes_gp2(100); | RI(2t)downUni | 171 |
| Otu031 | Bacteria(100);"Armatimonadetes"(100);Armatimonadetes_gp2_class_incertae_sedis(100);Armatimonadetes_gp2_order_incetae_sedis(100);Armatimonadetes_gp2_family_incetae_sedis(100);Armatimonadetes_gp2(100); | RI(2t)upUni | 1648 |
| Otu031 | Bacteria(100);"Armatimonadetes"(100);Armatimonadetes_gp2_class_incertae_sedis(100);Armatimonadetes_gp2_order_incetae_sedis(100);Armatimonadetes_gp2_family_incetae_sedis(100);Armatimonadetes_gp2(100); | RI(3t)downUni | 567 |
| Otu031 | Bacteria(100);"Armatimonadetes"(100);Armatimonadetes_gp2_class_incertae_sedis(100);Armatimonadetes_gp2_order_incetae_sedis(100);Armatimonadetes_gp2_family_incetae_sedis(100);Armatimonadetes_gp2(100); | RI(3t)upUni | 198 |
| Otu031 | Bacteria(100);"Armatimonadetes"(100);Armatimonadetes_gp2_class_incertae_sedis(100);Armatimonadetes_gp2_order_incetae_sedis(100);Armatimonadetes_gp2_family_incetae_sedis(100);Armatimonadetes_gp2(100); | RII(1t)downUni | 321 |
| Otu031 | Bacteria(100);"Armatimonadetes"(100);Armatimonadetes_gp2_class_incertae_sedis(100);Armatimonadetes_gp2_order_incetae_sedis(100);Armatimonadetes_gp2_family_incetae_sedis(100);Armatimonadetes_gp2(100); | RII(1t)upUni | 166 |
| Otu031 | Bacteria(100);"Armatimonadetes"(100);Armatimonadetes_gp2_class_incertae_sedis(100);Armatimonadetes_gp2_order_incetae_sedis(100);Armatimonadetes_gp2_family_incetae_sedis(100);Armatimonadetes_gp2(100); | RII(2t)downUni | 196 |
| Otu031 | Bacteria(100);"Armatimonadetes"(100);Armatimonadetes_gp2_class_incertae_sedis(100);Armatimonadetes_gp2_order_incetae_sedis(100);Armatimonadetes_gp2_family_incetae_sedis(100);Armatimonadetes_gp2(100); | RII(2t)upUni | 540 |
| Otu031 | Bacteria(100);"Armatimonadetes"(100);Armatimonadetes_gp2_class_incertae_sedis(100);Armatimonadetes_gp2_order_incetae_sedis(100);Armatimonadetes_gp2_family_incetae_sedis(100);Armatimonadetes_gp2(100); | RII(3t)downUni | 1145 |
| Otu031 | Bacteria(100);"Armatimonadetes"(100);Armatimonadetes_gp2_class_incertae_sedis(100);Armatimonadetes_gp2_order_incetae_sedis(100);Armatimonadetes_gp2_family_incetae_sedis(100);Armatimonadetes_gp2(100); | RII(3t)upUni | 541 |
| Otu031 | Bacteria(100);"Armatimonadetes"(100);Armatimonadetes_gp2_class_incertae_sedis(100);Armatimonadetes_gp2_order_incetae_sedis(100);Armatimonadetes_gp2_family_incetae_sedis(100);Armatimonadetes_gp2(100); | S1Uni | 730 |
| Otu031 | Bacteria(100);"Armatimonadetes"(100);Armatimonadetes_gp2_class_incertae_sedis(100);Armatimonadetes_gp2_order_incetae_sedis(100);Armatimonadetes_gp2_family_incetae_sedis(100);Armatimonadetes_gp2(100); | S2Uni | 747 |
| Otu032 | Bacteria(100);"Proteobacteria"(100);Deltaproteobacteria(100);Syntrophobacterales(100);Syntrophobacteraceae(100);Syntrophobacter(100); | RI(1t)downUni | 205 |
| Otu032 | Bacteria(100);"Proteobacteria"(100);Deltaproteobacteria(100);Syntrophobacterales(100);Syntrophobacteraceae(100);Syntrophobacter(100); | RI(1t)upUni | 43 |
| Otu032 | Bacteria(100);"Proteobacteria"(100);Deltaproteobacteria(100);Syntrophobacterales(100);Syntrophobacteraceae(100);Syntrophobacter(100); | RI(2t)downUni | 304 |
| Otu032 | Bacteria(100);"Proteobacteria"(100);Deltaproteobacteria(100);Syntrophobacterales(100);Syntrophobacteraceae(100);Syntrophobacter(100); | RI(2t)upUni | 1821 |
| Otu032 | Bacteria(100);"Proteobacteria"(100);Deltaproteobacteria(100);Syntrophobacterales(100);Syntrophobacteraceae(100);Syntrophobacter(100); | RI(3t)downUni | 150 |
| Otu032 | Bacteria(100);"Proteobacteria"(100);Deltaproteobacteria(100);Syntrophobacterales(100);Syntrophobacteraceae(100);Syntrophobacter(100); | RI(3t)upUni | 57 |
| Otu032 | Bacteria(100);"Proteobacteria"(100);Deltaproteobacteria(100);Syntrophobacterales(100);Syntrophobacteraceae(100);Syntrophobacter(100); | RII(1t)downUni | 701 |
| Otu032 | Bacteria(100);"Proteobacteria"(100);Deltaproteobacteria(100);Syntrophobacterales(100);Syntrophobacteraceae(100);Syntrophobacter(100); | RII(1t)upUni | 97 |
| Otu032 | Bacteria(100);"Proteobacteria"(100);Deltaproteobacteria(100);Syntrophobacterales(100);Syntrophobacteraceae(100);Syntrophobacter(100); | RII(2t)downUni | 184 |
| Otu032 | Bacteria(100);"Proteobacteria"(100);Deltaproteobacteria(100);Syntrophobacterales(100);Syntrophobacteraceae(100);Syntrophobacter(100); | RII(2t)upUni | 531 |
| Otu032 | Bacteria(100);"Proteobacteria"(100);Deltaproteobacteria(100);Syntrophobacterales(100);Syntrophobacteraceae(100);Syntrophobacter(100); | RII(3t)downUni | 209 |
| Otu032 | Bacteria(100);"Proteobacteria"(100);Deltaproteobacteria(100);Syntrophobacterales(100);Syntrophobacteraceae(100);Syntrophobacter(100); | RII(3t)upUni | 198 |
| Otu032 | Bacteria(100);"Proteobacteria"(100);Deltaproteobacteria(100);Syntrophobacterales(100);Syntrophobacteraceae(100);Syntrophobacter(100); | S1Uni | 2167 |
| Otu032 | Bacteria(100);"Proteobacteria"(100);Deltaproteobacteria(100);Syntrophobacterales(100);Syntrophobacteraceae(100);Syntrophobacter(100); | S2Uni | 2396 |
| Otu034 | Bacteria(100);"Proteobacteria"(100);Gammaproteobacteria(100);Pseudomonadales(100);Pseudomonadaceae(100);Pseudomonas(100); | RI(1t)downUni | 475 |
| Otu034 | Bacteria(100);"Proteobacteria"(100);Gammaproteobacteria(100);Pseudomonadales(100);Pseudomonadaceae(100);Pseudomonas(100); | RI(1t)upUni | 2413 |
| Otu034 | Bacteria(100);"Proteobacteria"(100);Gammaproteobacteria(100);Pseudomonadales(100);Pseudomonadaceae(100);Pseudomonas(100); | RI(2t)downUni | 266 |
| Otu034 | Bacteria(100);"Proteobacteria"(100);Gammaproteobacteria(100);Pseudomonadales(100);Pseudomonadaceae(100);Pseudomonas(100); | RI(2t)upUni | 832 |
| Otu034 | Bacteria(100);"Proteobacteria"(100);Gammaproteobacteria(100);Pseudomonadales(100);Pseudomonadaceae(100);Pseudomonas(100); | RI(3t)downUni | 505 |
| Otu034 | Bacteria(100);"Proteobacteria"(100);Gammaproteobacteria(100);Pseudomonadales(100);Pseudomonadaceae(100);Pseudomonas(100); | RI(3t)upUni | 1377 |
| Otu034 | Bacteria(100);"Proteobacteria"(100);Gammaproteobacteria(100);Pseudomonadales(100);Pseudomonadaceae(100);Pseudomonas(100); | RII(1t)downUni | 1420 |
| Otu034 | Bacteria(100);"Proteobacteria"(100);Gammaproteobacteria(100);Pseudomonadales(100);Pseudomonadaceae(100);Pseudomonas(100); | RII(1t)upUni | 2361 |
| Otu034 | Bacteria(100);"Proteobacteria"(100);Gammaproteobacteria(100);Pseudomonadales(100);Pseudomonadaceae(100);Pseudomonas(100); | RII(2t)downUni | 251 |
| Otu034 | Bacteria(100);"Proteobacteria"(100);Gammaproteobacteria(100);Pseudomonadales(100);Pseudomonadaceae(100);Pseudomonas(100); | RII(2t)upUni | 1036 |
| Otu034 | Bacteria(100);"Proteobacteria"(100);Gammaproteobacteria(100);Pseudomonadales(100);Pseudomonadaceae(100);Pseudomonas(100); | RII(3t)downUni | 6777 |
| Otu034 | Bacteria(100);"Proteobacteria"(100);Gammaproteobacteria(100);Pseudomonadales(100);Pseudomonadaceae(100);Pseudomonas(100); | RII(3t)upUni | 70636 |
| Otu034 | Bacteria(100);"Proteobacteria"(100);Gammaproteobacteria(100);Pseudomonadales(100);Pseudomonadaceae(100);Pseudomonas(100); | S1Uni | 504 |
| Otu034 | Bacteria(100);"Proteobacteria"(100);Gammaproteobacteria(100);Pseudomonadales(100);Pseudomonadaceae(100);Pseudomonas(100); | S2Uni | 471 |
| Otu035 | Bacteria(100);"Proteobacteria"(100);Deltaproteobacteria(100);Syntrophobacterales(100);Syntrophaceae(100);Smithella(100); | RI(1t)downUni | 163 |
| Otu035 | Bacteria(100);"Proteobacteria"(100);Deltaproteobacteria(100);Syntrophobacterales(100);Syntrophaceae(100);Smithella(100); | RI(1t)upUni | 47 |
| Otu035 | Bacteria(100);"Proteobacteria"(100);Deltaproteobacteria(100);Syntrophobacterales(100);Syntrophaceae(100);Smithella(100); | RI(2t)downUni | 21 |
| Otu035 | Bacteria(100);"Proteobacteria"(100);Deltaproteobacteria(100);Syntrophobacterales(100);Syntrophaceae(100);Smithella(100); | RI(2t)upUni | 50 |
| Otu035 | Bacteria(100);"Proteobacteria"(100);Deltaproteobacteria(100);Syntrophobacterales(100);Syntrophaceae(100);Smithella(100); | RI(3t)downUni | 12 |
| Otu035 | Bacteria(100);"Proteobacteria"(100);Deltaproteobacteria(100);Syntrophobacterales(100);Syntrophaceae(100);Smithella(100); | RI(3t)upUni | 10 |
| Otu035 | Bacteria(100);"Proteobacteria"(100);Deltaproteobacteria(100);Syntrophobacterales(100);Syntrophaceae(100);Smithella(100); | RII(1t)downUni | 448 |
| Otu035 | Bacteria(100);"Proteobacteria"(100);Deltaproteobacteria(100);Syntrophobacterales(100);Syntrophaceae(100);Smithella(100); | RII(1t)upUni | 87 |
| Otu035 | Bacteria(100);"Proteobacteria"(100);Deltaproteobacteria(100);Syntrophobacterales(100);Syntrophaceae(100);Smithella(100); | RII(2t)downUni | 19 |
| Otu035 | Bacteria(100);"Proteobacteria"(100);Deltaproteobacteria(100);Syntrophobacterales(100);Syntrophaceae(100);Smithella(100); | RII(2t)upUni | 86 |
| Otu035 | Bacteria(100);"Proteobacteria"(100);Deltaproteobacteria(100);Syntrophobacterales(100);Syntrophaceae(100);Smithella(100); | RII(3t)downUni | 10 |
| Otu035 | Bacteria(100);"Proteobacteria"(100);Deltaproteobacteria(100);Syntrophobacterales(100);Syntrophaceae(100);Smithella(100); | RII(3t)upUni | 5 |
| Otu035 | Bacteria(100);"Proteobacteria"(100);Deltaproteobacteria(100);Syntrophobacterales(100);Syntrophaceae(100);Smithella(100); | S1Uni | 2792 |
| Otu035 | Bacteria(100);"Proteobacteria"(100);Deltaproteobacteria(100);Syntrophobacterales(100);Syntrophaceae(100);Smithella(100); | S2Uni | 2972 |
| Otu039 | Bacteria(100);Firmicutes(100);Clostridia(100);Clostridiales(100);Clostridiaceae_1(100);Clostridium_sensu_stricto(100); | RI(1t)downUni | 149 |
| Otu039 | Bacteria(100);Firmicutes(100);Clostridia(100);Clostridiales(100);Clostridiaceae_1(100);Clostridium_sensu_stricto(100); | RI(1t)upUni | 278 |
| Otu039 | Bacteria(100);Firmicutes(100);Clostridia(100);Clostridiales(100);Clostridiaceae_1(100);Clostridium_sensu_stricto(100); | RI(2t)downUni | 109 |
| Otu039 | Bacteria(100);Firmicutes(100);Clostridia(100);Clostridiales(100);Clostridiaceae_1(100);Clostridium_sensu_stricto(100); | RI(2t)upUni | 741 |
| Otu039 | Bacteria(100);Firmicutes(100);Clostridia(100);Clostridiales(100);Clostridiaceae_1(100);Clostridium_sensu_stricto(100); | RI(3t)downUni | 116 |
| Otu039 | Bacteria(100);Firmicutes(100);Clostridia(100);Clostridiales(100);Clostridiaceae_1(100);Clostridium_sensu_stricto(100); | RI(3t)upUni | 650 |
| Otu039 | Bacteria(100);Firmicutes(100);Clostridia(100);Clostridiales(100);Clostridiaceae_1(100);Clostridium_sensu_stricto(100); | RII(1t)downUni | 1031 |
| Otu039 | Bacteria(100);Firmicutes(100);Clostridia(100);Clostridiales(100);Clostridiaceae_1(100);Clostridium_sensu_stricto(100); | RII(1t)upUni | 607 |
| Otu039 | Bacteria(100);Firmicutes(100);Clostridia(100);Clostridiales(100);Clostridiaceae_1(100);Clostridium_sensu_stricto(100); | RII(2t)downUni | 142 |
| Otu039 | Bacteria(100);Firmicutes(100);Clostridia(100);Clostridiales(100);Clostridiaceae_1(100);Clostridium_sensu_stricto(100); | RII(2t)upUni | 566 |
| Otu039 | Bacteria(100);Firmicutes(100);Clostridia(100);Clostridiales(100);Clostridiaceae_1(100);Clostridium_sensu_stricto(100); | RII(3t)downUni | 214 |
| Otu039 | Bacteria(100);Firmicutes(100);Clostridia(100);Clostridiales(100);Clostridiaceae_1(100);Clostridium_sensu_stricto(100); | RII(3t)upUni | 1063 |
| Otu039 | Bacteria(100);Firmicutes(100);Clostridia(100);Clostridiales(100);Clostridiaceae_1(100);Clostridium_sensu_stricto(100); | S1Uni | 1524 |
| Otu039 | Bacteria(100);Firmicutes(100);Clostridia(100);Clostridiales(100);Clostridiaceae_1(100);Clostridium_sensu_stricto(100); | S2Uni | 1934 |
| Otu040 | Bacteria(100);"Bacteroidetes"(100);"Bacteroidia"(100);"Bacteroidales"(100);"Porphyromonadaceae"(100);Paludibacter(100); | RI(1t)downUni | 2094 |
| Otu040 | Bacteria(100);"Bacteroidetes"(100);"Bacteroidia"(100);"Bacteroidales"(100);"Porphyromonadaceae"(100);Paludibacter(100); | RI(1t)upUni | 11831 |
| Otu040 | Bacteria(100);"Bacteroidetes"(100);"Bacteroidia"(100);"Bacteroidales"(100);"Porphyromonadaceae"(100);Paludibacter(100); | RI(2t)downUni | 247 |
| Otu040 | Bacteria(100);"Bacteroidetes"(100);"Bacteroidia"(100);"Bacteroidales"(100);"Porphyromonadaceae"(100);Paludibacter(100); | RI(2t)upUni | 483 |
| Otu040 | Bacteria(100);"Bacteroidetes"(100);"Bacteroidia"(100);"Bacteroidales"(100);"Porphyromonadaceae"(100);Paludibacter(100); | RI(3t)downUni | 86 |
| Otu040 | Bacteria(100);"Bacteroidetes"(100);"Bacteroidia"(100);"Bacteroidales"(100);"Porphyromonadaceae"(100);Paludibacter(100); | RI(3t)upUni | 88 |
| Otu040 | Bacteria(100);"Bacteroidetes"(100);"Bacteroidia"(100);"Bacteroidales"(100);"Porphyromonadaceae"(100);Paludibacter(100); | RII(1t)downUni | 2945 |
| Otu040 | Bacteria(100);"Bacteroidetes"(100);"Bacteroidia"(100);"Bacteroidales"(100);"Porphyromonadaceae"(100);Paludibacter(100); | RII(1t)upUni | 6522 |
| Otu040 | Bacteria(100);"Bacteroidetes"(100);"Bacteroidia"(100);"Bacteroidales"(100);"Porphyromonadaceae"(100);Paludibacter(100); | RII(2t)downUni | 683 |
| Otu040 | Bacteria(100);"Bacteroidetes"(100);"Bacteroidia"(100);"Bacteroidales"(100);"Porphyromonadaceae"(100);Paludibacter(100); | RII(2t)upUni | 2702 |
| Otu040 | Bacteria(100);"Bacteroidetes"(100);"Bacteroidia"(100);"Bacteroidales"(100);"Porphyromonadaceae"(100);Paludibacter(100); | RII(3t)downUni | 169 |
| Otu040 | Bacteria(100);"Bacteroidetes"(100);"Bacteroidia"(100);"Bacteroidales"(100);"Porphyromonadaceae"(100);Paludibacter(100); | RII(3t)upUni | 546 |
| Otu040 | Bacteria(100);"Bacteroidetes"(100);"Bacteroidia"(100);"Bacteroidales"(100);"Porphyromonadaceae"(100);Paludibacter(100); | S1Uni | 228 |
| Otu040 | Bacteria(100);"Bacteroidetes"(100);"Bacteroidia"(100);"Bacteroidales"(100);"Porphyromonadaceae"(100);Paludibacter(100); | S2Uni | 311 |
| Otu041 | Bacteria(100);"Bacteroidetes"(100);"Bacteroidia"(100);"Bacteroidales"(100);"Porphyromonadaceae"(100);Parabacteroides(100); | RI(1t)downUni | 4111 |
| Otu041 | Bacteria(100);"Bacteroidetes"(100);"Bacteroidia"(100);"Bacteroidales"(100);"Porphyromonadaceae"(100);Parabacteroides(100); | RI(1t)upUni | 14715 |
| Otu041 | Bacteria(100);"Bacteroidetes"(100);"Bacteroidia"(100);"Bacteroidales"(100);"Porphyromonadaceae"(100);Parabacteroides(100); | RI(2t)downUni | 907 |
| Otu041 | Bacteria(100);"Bacteroidetes"(100);"Bacteroidia"(100);"Bacteroidales"(100);"Porphyromonadaceae"(100);Parabacteroides(100); | RI(2t)upUni | 5434 |
| Otu041 | Bacteria(100);"Bacteroidetes"(100);"Bacteroidia"(100);"Bacteroidales"(100);"Porphyromonadaceae"(100);Parabacteroides(100); | RI(3t)downUni | 507 |
| Otu041 | Bacteria(100);"Bacteroidetes"(100);"Bacteroidia"(100);"Bacteroidales"(100);"Porphyromonadaceae"(100);Parabacteroides(100); | RI(3t)upUni | 298 |
| Otu041 | Bacteria(100);"Bacteroidetes"(100);"Bacteroidia"(100);"Bacteroidales"(100);"Porphyromonadaceae"(100);Parabacteroides(100); | RII(1t)downUni | 13234 |
| Otu041 | Bacteria(100);"Bacteroidetes"(100);"Bacteroidia"(100);"Bacteroidales"(100);"Porphyromonadaceae"(100);Parabacteroides(100); | RII(1t)upUni | 9530 |
| Otu041 | Bacteria(100);"Bacteroidetes"(100);"Bacteroidia"(100);"Bacteroidales"(100);"Porphyromonadaceae"(100);Parabacteroides(100); | RII(2t)downUni | 1205 |
| Otu041 | Bacteria(100);"Bacteroidetes"(100);"Bacteroidia"(100);"Bacteroidales"(100);"Porphyromonadaceae"(100);Parabacteroides(100); | RII(2t)upUni | 5158 |
| Otu041 | Bacteria(100);"Bacteroidetes"(100);"Bacteroidia"(100);"Bacteroidales"(100);"Porphyromonadaceae"(100);Parabacteroides(100); | RII(3t)downUni | 898 |
| Otu041 | Bacteria(100);"Bacteroidetes"(100);"Bacteroidia"(100);"Bacteroidales"(100);"Porphyromonadaceae"(100);Parabacteroides(100); | RII(3t)upUni | 1187 |
| Otu041 | Bacteria(100);"Bacteroidetes"(100);"Bacteroidia"(100);"Bacteroidales"(100);"Porphyromonadaceae"(100);Parabacteroides(100); | S1Uni | 12 |
| Otu041 | Bacteria(100);"Bacteroidetes"(100);"Bacteroidia"(100);"Bacteroidales"(100);"Porphyromonadaceae"(100);Parabacteroides(100); | S2Uni | 16 |
| Otu042 | Bacteria(100);"Proteobacteria"(100);Deltaproteobacteria(100);Desulfobacterales(100);Desulfobacteraceae(100);unclassified(100); | RI(1t)downUni | 40 |
| Otu042 | Bacteria(100);"Proteobacteria"(100);Deltaproteobacteria(100);Desulfobacterales(100);Desulfobacteraceae(100);unclassified(100); | RI(1t)upUni | 23 |
| Otu042 | Bacteria(100);"Proteobacteria"(100);Deltaproteobacteria(100);Desulfobacterales(100);Desulfobacteraceae(100);unclassified(100); | RI(2t)downUni | 14 |
| Otu042 | Bacteria(100);"Proteobacteria"(100);Deltaproteobacteria(100);Desulfobacterales(100);Desulfobacteraceae(100);unclassified(100); | RI(2t)upUni | 58 |
| Otu042 | Bacteria(100);"Proteobacteria"(100);Deltaproteobacteria(100);Desulfobacterales(100);Desulfobacteraceae(100);unclassified(100); | RI(3t)downUni | 5 |
| Otu042 | Bacteria(100);"Proteobacteria"(100);Deltaproteobacteria(100);Desulfobacterales(100);Desulfobacteraceae(100);unclassified(100); | RI(3t)upUni | 5 |
| Otu042 | Bacteria(100);"Proteobacteria"(100);Deltaproteobacteria(100);Desulfobacterales(100);Desulfobacteraceae(100);unclassified(100); | RII(1t)downUni | 265 |
| Otu042 | Bacteria(100);"Proteobacteria"(100);Deltaproteobacteria(100);Desulfobacterales(100);Desulfobacteraceae(100);unclassified(100); | RII(1t)upUni | 50 |
| Otu042 | Bacteria(100);"Proteobacteria"(100);Deltaproteobacteria(100);Desulfobacterales(100);Desulfobacteraceae(100);unclassified(100); | RII(2t)downUni | 8 |
| Otu042 | Bacteria(100);"Proteobacteria"(100);Deltaproteobacteria(100);Desulfobacterales(100);Desulfobacteraceae(100);unclassified(100); | RII(2t)upUni | 58 |
| Otu042 | Bacteria(100);"Proteobacteria"(100);Deltaproteobacteria(100);Desulfobacterales(100);Desulfobacteraceae(100);unclassified(100); | RII(3t)downUni | 8 |
| Otu042 | Bacteria(100);"Proteobacteria"(100);Deltaproteobacteria(100);Desulfobacterales(100);Desulfobacteraceae(100);unclassified(100); | RII(3t)upUni | 16 |
| Otu042 | Bacteria(100);"Proteobacteria"(100);Deltaproteobacteria(100);Desulfobacterales(100);Desulfobacteraceae(100);unclassified(100); | S1Uni | 2425 |
| Otu042 | Bacteria(100);"Proteobacteria"(100);Deltaproteobacteria(100);Desulfobacterales(100);Desulfobacteraceae(100);unclassified(100); | S2Uni | 3362 |
| Otu043 | Bacteria(100);"Synergistetes"(100);Synergistia(100);Synergistales(100);Synergistaceae(100);Aminiphilus(100); | RI(1t)downUni | 54 |
| Otu043 | Bacteria(100);"Synergistetes"(100);Synergistia(100);Synergistales(100);Synergistaceae(100);Aminiphilus(100); | RI(1t)upUni | 11 |
| Otu043 | Bacteria(100);"Synergistetes"(100);Synergistia(100);Synergistales(100);Synergistaceae(100);Aminiphilus(100); | RI(2t)downUni | 813 |
| Otu043 | Bacteria(100);"Synergistetes"(100);Synergistia(100);Synergistales(100);Synergistaceae(100);Aminiphilus(100); | RI(2t)upUni | 4295 |
| Otu043 | Bacteria(100);"Synergistetes"(100);Synergistia(100);Synergistales(100);Synergistaceae(100);Aminiphilus(100); | RI(3t)downUni | 690 |
| Otu043 | Bacteria(100);"Synergistetes"(100);Synergistia(100);Synergistales(100);Synergistaceae(100);Aminiphilus(100); | RI(3t)upUni | 842 |
| Otu043 | Bacteria(100);"Synergistetes"(100);Synergistia(100);Synergistales(100);Synergistaceae(100);Aminiphilus(100); | RII(1t)downUni | 78 |
| Otu043 | Bacteria(100);"Synergistetes"(100);Synergistia(100);Synergistales(100);Synergistaceae(100);Aminiphilus(100); | RII(1t)upUni | 40 |
| Otu043 | Bacteria(100);"Synergistetes"(100);Synergistia(100);Synergistales(100);Synergistaceae(100);Aminiphilus(100); | RII(2t)downUni | 692 |
| Otu043 | Bacteria(100);"Synergistetes"(100);Synergistia(100);Synergistales(100);Synergistaceae(100);Aminiphilus(100); | RII(2t)upUni | 1487 |
| Otu043 | Bacteria(100);"Synergistetes"(100);Synergistia(100);Synergistales(100);Synergistaceae(100);Aminiphilus(100); | RII(3t)downUni | 659 |
| Otu043 | Bacteria(100);"Synergistetes"(100);Synergistia(100);Synergistales(100);Synergistaceae(100);Aminiphilus(100); | RII(3t)upUni | 1045 |
| Otu043 | Bacteria(100);"Synergistetes"(100);Synergistia(100);Synergistales(100);Synergistaceae(100);Aminiphilus(100); | S1Uni | 1 |
| Otu043 | Bacteria(100);"Synergistetes"(100);Synergistia(100);Synergistales(100);Synergistaceae(100);Aminiphilus(100); | S2Uni | 1 |
| Otu046 | Bacteria(100);Firmicutes(100);Clostridia(100);Clostridiales(100);Gracilibacteraceae(100);Gracilibacter(100); | RI(1t)downUni | 129 |
| Otu046 | Bacteria(100);Firmicutes(100);Clostridia(100);Clostridiales(100);Gracilibacteraceae(100);Gracilibacter(100); | RI(1t)upUni | 53 |
| Otu046 | Bacteria(100);Firmicutes(100);Clostridia(100);Clostridiales(100);Gracilibacteraceae(100);Gracilibacter(100); | RI(2t)downUni | 7 |
| Otu046 | Bacteria(100);Firmicutes(100);Clostridia(100);Clostridiales(100);Gracilibacteraceae(100);Gracilibacter(100); | RI(2t)upUni | 158 |
| Otu046 | Bacteria(100);Firmicutes(100);Clostridia(100);Clostridiales(100);Gracilibacteraceae(100);Gracilibacter(100); | RI(3t)downUni | 19 |
| Otu046 | Bacteria(100);Firmicutes(100);Clostridia(100);Clostridiales(100);Gracilibacteraceae(100);Gracilibacter(100); | RI(3t)upUni | 9 |
| Otu046 | Bacteria(100);Firmicutes(100);Clostridia(100);Clostridiales(100);Gracilibacteraceae(100);Gracilibacter(100); | RII(1t)downUni | 640 |
| Otu046 | Bacteria(100);Firmicutes(100);Clostridia(100);Clostridiales(100);Gracilibacteraceae(100);Gracilibacter(100); | RII(1t)upUni | 41 |
| Otu046 | Bacteria(100);Firmicutes(100);Clostridia(100);Clostridiales(100);Gracilibacteraceae(100);Gracilibacter(100); | RII(2t)downUni | 9 |
| Otu046 | Bacteria(100);Firmicutes(100);Clostridia(100);Clostridiales(100);Gracilibacteraceae(100);Gracilibacter(100); | RII(2t)upUni | 23 |
| Otu046 | Bacteria(100);Firmicutes(100);Clostridia(100);Clostridiales(100);Gracilibacteraceae(100);Gracilibacter(100); | RII(3t)downUni | 21 |
| Otu046 | Bacteria(100);Firmicutes(100);Clostridia(100);Clostridiales(100);Gracilibacteraceae(100);Gracilibacter(100); | RII(3t)upUni | 22 |
| Otu046 | Bacteria(100);Firmicutes(100);Clostridia(100);Clostridiales(100);Gracilibacteraceae(100);Gracilibacter(100); | S1Uni | 14 |
| Otu046 | Bacteria(100);Firmicutes(100);Clostridia(100);Clostridiales(100);Gracilibacteraceae(100);Gracilibacter(100); | S2Uni | 9 |
| Otu051 | Bacteria(100);"Synergistetes"(100);Synergistia(100);Synergistales(100);Synergistaceae(100);Cloacibacillus(100); | RI(1t)downUni | 53 |
| Otu051 | Bacteria(100);"Synergistetes"(100);Synergistia(100);Synergistales(100);Synergistaceae(100);Cloacibacillus(100); | RI(1t)upUni | 853 |
| Otu051 | Bacteria(100);"Synergistetes"(100);Synergistia(100);Synergistales(100);Synergistaceae(100);Cloacibacillus(100); | RI(2t)downUni | 75 |
| Otu051 | Bacteria(100);"Synergistetes"(100);Synergistia(100);Synergistales(100);Synergistaceae(100);Cloacibacillus(100); | RI(2t)upUni | 1618 |
| Otu051 | Bacteria(100);"Synergistetes"(100);Synergistia(100);Synergistales(100);Synergistaceae(100);Cloacibacillus(100); | RI(3t)downUni | 202 |
| Otu051 | Bacteria(100);"Synergistetes"(100);Synergistia(100);Synergistales(100);Synergistaceae(100);Cloacibacillus(100); | RI(3t)upUni | 62 |
| Otu051 | Bacteria(100);"Synergistetes"(100);Synergistia(100);Synergistales(100);Synergistaceae(100);Cloacibacillus(100); | RII(1t)downUni | 1480 |
| Otu051 | Bacteria(100);"Synergistetes"(100);Synergistia(100);Synergistales(100);Synergistaceae(100);Cloacibacillus(100); | RII(1t)upUni | 328 |
| Otu051 | Bacteria(100);"Synergistetes"(100);Synergistia(100);Synergistales(100);Synergistaceae(100);Cloacibacillus(100); | RII(2t)downUni | 2963 |
| Otu051 | Bacteria(100);"Synergistetes"(100);Synergistia(100);Synergistales(100);Synergistaceae(100);Cloacibacillus(100); | RII(2t)upUni | 435 |
| Otu051 | Bacteria(100);"Synergistetes"(100);Synergistia(100);Synergistales(100);Synergistaceae(100);Cloacibacillus(100); | RII(3t)downUni | 23 |
| Otu051 | Bacteria(100);"Synergistetes"(100);Synergistia(100);Synergistales(100);Synergistaceae(100);Cloacibacillus(100); | RII(3t)upUni | 109 |
| Otu051 | Bacteria(100);"Synergistetes"(100);Synergistia(100);Synergistales(100);Synergistaceae(100);Cloacibacillus(100); | S1Uni | 4 |
| Otu051 | Bacteria(100);"Synergistetes"(100);Synergistia(100);Synergistales(100);Synergistaceae(100);Cloacibacillus(100); | S2Uni | 5 |
| Otu052 | Bacteria(100);"Proteobacteria"(100);Deltaproteobacteria(100);Desulfovibrionales(100);Desulfovibrionaceae(100);Desulfovibrio(100); | RI(1t)downUni | 190 |
| Otu052 | Bacteria(100);"Proteobacteria"(100);Deltaproteobacteria(100);Desulfovibrionales(100);Desulfovibrionaceae(100);Desulfovibrio(100); | RI(1t)upUni | 1250 |
| Otu052 | Bacteria(100);"Proteobacteria"(100);Deltaproteobacteria(100);Desulfovibrionales(100);Desulfovibrionaceae(100);Desulfovibrio(100); | RI(2t)downUni | 127 |
| Otu052 | Bacteria(100);"Proteobacteria"(100);Deltaproteobacteria(100);Desulfovibrionales(100);Desulfovibrionaceae(100);Desulfovibrio(100); | RI(2t)upUni | 1389 |
| Otu052 | Bacteria(100);"Proteobacteria"(100);Deltaproteobacteria(100);Desulfovibrionales(100);Desulfovibrionaceae(100);Desulfovibrio(100); | RI(3t)downUni | 57 |
| Otu052 | Bacteria(100);"Proteobacteria"(100);Deltaproteobacteria(100);Desulfovibrionales(100);Desulfovibrionaceae(100);Desulfovibrio(100); | RI(3t)upUni | 113 |
| Otu052 | Bacteria(100);"Proteobacteria"(100);Deltaproteobacteria(100);Desulfovibrionales(100);Desulfovibrionaceae(100);Desulfovibrio(100); | RII(1t)downUni | 452 |
| Otu052 | Bacteria(100);"Proteobacteria"(100);Deltaproteobacteria(100);Desulfovibrionales(100);Desulfovibrionaceae(100);Desulfovibrio(100); | RII(1t)upUni | 1059 |
| Otu052 | Bacteria(100);"Proteobacteria"(100);Deltaproteobacteria(100);Desulfovibrionales(100);Desulfovibrionaceae(100);Desulfovibrio(100); | RII(2t)downUni | 239 |
| Otu052 | Bacteria(100);"Proteobacteria"(100);Deltaproteobacteria(100);Desulfovibrionales(100);Desulfovibrionaceae(100);Desulfovibrio(100); | RII(2t)upUni | 967 |
| Otu052 | Bacteria(100);"Proteobacteria"(100);Deltaproteobacteria(100);Desulfovibrionales(100);Desulfovibrionaceae(100);Desulfovibrio(100); | RII(3t)downUni | 146 |
| Otu052 | Bacteria(100);"Proteobacteria"(100);Deltaproteobacteria(100);Desulfovibrionales(100);Desulfovibrionaceae(100);Desulfovibrio(100); | RII(3t)upUni | 394 |
| Otu052 | Bacteria(100);"Proteobacteria"(100);Deltaproteobacteria(100);Desulfovibrionales(100);Desulfovibrionaceae(100);Desulfovibrio(100); | S1Uni | 171 |
| Otu052 | Bacteria(100);"Proteobacteria"(100);Deltaproteobacteria(100);Desulfovibrionales(100);Desulfovibrionaceae(100);Desulfovibrio(100); | S2Uni | 220 |
| Otu054 | Bacteria(100);"Proteobacteria"(100);Deltaproteobacteria(100);Desulfovibrionales(100);Desulfomicrobiaceae(100);Desulfomicrobium(100); | RI(1t)downUni | 192 |
| Otu054 | Bacteria(100);"Proteobacteria"(100);Deltaproteobacteria(100);Desulfovibrionales(100);Desulfomicrobiaceae(100);Desulfomicrobium(100); | RI(1t)upUni | 50 |
| Otu054 | Bacteria(100);"Proteobacteria"(100);Deltaproteobacteria(100);Desulfovibrionales(100);Desulfomicrobiaceae(100);Desulfomicrobium(100); | RI(2t)downUni | 63 |
| Otu054 | Bacteria(100);"Proteobacteria"(100);Deltaproteobacteria(100);Desulfovibrionales(100);Desulfomicrobiaceae(100);Desulfomicrobium(100); | RI(2t)upUni | 370 |
| Otu054 | Bacteria(100);"Proteobacteria"(100);Deltaproteobacteria(100);Desulfovibrionales(100);Desulfomicrobiaceae(100);Desulfomicrobium(100); | RI(3t)downUni | 72 |
| Otu054 | Bacteria(100);"Proteobacteria"(100);Deltaproteobacteria(100);Desulfovibrionales(100);Desulfomicrobiaceae(100);Desulfomicrobium(100); | RI(3t)upUni | 14 |
| Otu054 | Bacteria(100);"Proteobacteria"(100);Deltaproteobacteria(100);Desulfovibrionales(100);Desulfomicrobiaceae(100);Desulfomicrobium(100); | RII(1t)downUni | 527 |
| Otu054 | Bacteria(100);"Proteobacteria"(100);Deltaproteobacteria(100);Desulfovibrionales(100);Desulfomicrobiaceae(100);Desulfomicrobium(100); | RII(1t)upUni | 380 |
| Otu054 | Bacteria(100);"Proteobacteria"(100);Deltaproteobacteria(100);Desulfovibrionales(100);Desulfomicrobiaceae(100);Desulfomicrobium(100); | RII(2t)downUni | 147 |
| Otu054 | Bacteria(100);"Proteobacteria"(100);Deltaproteobacteria(100);Desulfovibrionales(100);Desulfomicrobiaceae(100);Desulfomicrobium(100); | RII(2t)upUni | 517 |
| Otu054 | Bacteria(100);"Proteobacteria"(100);Deltaproteobacteria(100);Desulfovibrionales(100);Desulfomicrobiaceae(100);Desulfomicrobium(100); | RII(3t)downUni | 227 |
| Otu054 | Bacteria(100);"Proteobacteria"(100);Deltaproteobacteria(100);Desulfovibrionales(100);Desulfomicrobiaceae(100);Desulfomicrobium(100); | RII(3t)upUni | 71 |
| Otu054 | Bacteria(100);"Proteobacteria"(100);Deltaproteobacteria(100);Desulfovibrionales(100);Desulfomicrobiaceae(100);Desulfomicrobium(100); | S1Uni | 970 |
| Otu054 | Bacteria(100);"Proteobacteria"(100);Deltaproteobacteria(100);Desulfovibrionales(100);Desulfomicrobiaceae(100);Desulfomicrobium(100); | S2Uni | 1164 |
| Otu057 | Bacteria(100);Firmicutes(100);Clostridia(100);Clostridiales(100);Clostridiaceae_1(100);unclassified(100); | RI(1t)downUni | 42 |
| Otu057 | Bacteria(100);Firmicutes(100);Clostridia(100);Clostridiales(100);Clostridiaceae_1(100);unclassified(100); | RI(1t)upUni | 88 |
| Otu057 | Bacteria(100);Firmicutes(100);Clostridia(100);Clostridiales(100);Clostridiaceae_1(100);unclassified(100); | RI(2t)downUni | 11 |
| Otu057 | Bacteria(100);Firmicutes(100);Clostridia(100);Clostridiales(100);Clostridiaceae_1(100);unclassified(100); | RI(2t)upUni | 98 |
| Otu057 | Bacteria(100);Firmicutes(100);Clostridia(100);Clostridiales(100);Clostridiaceae_1(100);unclassified(100); | RI(3t)downUni | 6 |
| Otu057 | Bacteria(100);Firmicutes(100);Clostridia(100);Clostridiales(100);Clostridiaceae_1(100);unclassified(100); | RI(3t)upUni | 13 |
| Otu057 | Bacteria(100);Firmicutes(100);Clostridia(100);Clostridiales(100);Clostridiaceae_1(100);unclassified(100); | RII(1t)downUni | 550 |
| Otu057 | Bacteria(100);Firmicutes(100);Clostridia(100);Clostridiales(100);Clostridiaceae_1(100);unclassified(100); | RII(1t)upUni | 113 |
| Otu057 | Bacteria(100);Firmicutes(100);Clostridia(100);Clostridiales(100);Clostridiaceae_1(100);unclassified(100); | RII(2t)downUni | 27 |
| Otu057 | Bacteria(100);Firmicutes(100);Clostridia(100);Clostridiales(100);Clostridiaceae_1(100);unclassified(100); | RII(2t)upUni | 120 |
| Otu057 | Bacteria(100);Firmicutes(100);Clostridia(100);Clostridiales(100);Clostridiaceae_1(100);unclassified(100); | RII(3t)downUni | 17 |
| Otu057 | Bacteria(100);Firmicutes(100);Clostridia(100);Clostridiales(100);Clostridiaceae_1(100);unclassified(100); | RII(3t)upUni | 29 |
| Otu057 | Bacteria(100);Firmicutes(100);Clostridia(100);Clostridiales(100);Clostridiaceae_1(100);unclassified(100); | S1Uni | 247 |
| Otu057 | Bacteria(100);Firmicutes(100);Clostridia(100);Clostridiales(100);Clostridiaceae_1(100);unclassified(100); | S2Uni | 564 |
| Otu058 | Bacteria(100);"Proteobacteria"(100);Deltaproteobacteria(100);Desulfuromonadales(100);Desulfuromonadaceae(100);Desulfuromonas(100); | RI(1t)downUni | 3 |
| Otu058 | Bacteria(100);"Proteobacteria"(100);Deltaproteobacteria(100);Desulfuromonadales(100);Desulfuromonadaceae(100);Desulfuromonas(100); | RI(1t)upUni | 2 |
| Otu058 | Bacteria(100);"Proteobacteria"(100);Deltaproteobacteria(100);Desulfuromonadales(100);Desulfuromonadaceae(100);Desulfuromonas(100); | RI(2t)downUni | 519 |
| Otu058 | Bacteria(100);"Proteobacteria"(100);Deltaproteobacteria(100);Desulfuromonadales(100);Desulfuromonadaceae(100);Desulfuromonas(100); | RI(2t)upUni | 2423 |
| Otu058 | Bacteria(100);"Proteobacteria"(100);Deltaproteobacteria(100);Desulfuromonadales(100);Desulfuromonadaceae(100);Desulfuromonas(100); | RI(3t)downUni | 1055 |
| Otu058 | Bacteria(100);"Proteobacteria"(100);Deltaproteobacteria(100);Desulfuromonadales(100);Desulfuromonadaceae(100);Desulfuromonas(100); | RI(3t)upUni | 389 |
| Otu058 | Bacteria(100);"Proteobacteria"(100);Deltaproteobacteria(100);Desulfuromonadales(100);Desulfuromonadaceae(100);Desulfuromonas(100); | RII(1t)downUni | 10 |
| Otu058 | Bacteria(100);"Proteobacteria"(100);Deltaproteobacteria(100);Desulfuromonadales(100);Desulfuromonadaceae(100);Desulfuromonas(100); | RII(1t)upUni | 1 |
| Otu058 | Bacteria(100);"Proteobacteria"(100);Deltaproteobacteria(100);Desulfuromonadales(100);Desulfuromonadaceae(100);Desulfuromonas(100); | RII(2t)downUni | 113 |
| Otu058 | Bacteria(100);"Proteobacteria"(100);Deltaproteobacteria(100);Desulfuromonadales(100);Desulfuromonadaceae(100);Desulfuromonas(100); | RII(2t)upUni | 562 |
| Otu058 | Bacteria(100);"Proteobacteria"(100);Deltaproteobacteria(100);Desulfuromonadales(100);Desulfuromonadaceae(100);Desulfuromonas(100); | RII(3t)downUni | 2297 |
| Otu058 | Bacteria(100);"Proteobacteria"(100);Deltaproteobacteria(100);Desulfuromonadales(100);Desulfuromonadaceae(100);Desulfuromonas(100); | RII(3t)upUni | 720 |
| Otu058 | Bacteria(100);"Proteobacteria"(100);Deltaproteobacteria(100);Desulfuromonadales(100);Desulfuromonadaceae(100);Desulfuromonas(100); | S1Uni | 7 |
| Otu058 | Bacteria(100);"Proteobacteria"(100);Deltaproteobacteria(100);Desulfuromonadales(100);Desulfuromonadaceae(100);Desulfuromonas(100); | S2Uni | 24 |
| Otu061 | Bacteria(100);Firmicutes(100);Clostridia(100);Clostridiales(100);Ruminococcaceae(100);Clostridium_III(100); | RI(1t)downUni | 272 |
| Otu061 | Bacteria(100);Firmicutes(100);Clostridia(100);Clostridiales(100);Ruminococcaceae(100);Clostridium_III(100); | RI(1t)upUni | 364 |
| Otu061 | Bacteria(100);Firmicutes(100);Clostridia(100);Clostridiales(100);Ruminococcaceae(100);Clostridium_III(100); | RI(2t)downUni | 57 |
| Otu061 | Bacteria(100);Firmicutes(100);Clostridia(100);Clostridiales(100);Ruminococcaceae(100);Clostridium_III(100); | RI(2t)upUni | 399 |
| Otu061 | Bacteria(100);Firmicutes(100);Clostridia(100);Clostridiales(100);Ruminococcaceae(100);Clostridium_III(100); | RI(3t)downUni | 35 |
| Otu061 | Bacteria(100);Firmicutes(100);Clostridia(100);Clostridiales(100);Ruminococcaceae(100);Clostridium_III(100); | RI(3t)upUni | 71 |
| Otu061 | Bacteria(100);Firmicutes(100);Clostridia(100);Clostridiales(100);Ruminococcaceae(100);Clostridium_III(100); | RII(1t)downUni | 631 |
| Otu061 | Bacteria(100);Firmicutes(100);Clostridia(100);Clostridiales(100);Ruminococcaceae(100);Clostridium_III(100); | RII(1t)upUni | 565 |
| Otu061 | Bacteria(100);Firmicutes(100);Clostridia(100);Clostridiales(100);Ruminococcaceae(100);Clostridium_III(100); | RII(2t)downUni | 152 |
| Otu061 | Bacteria(100);Firmicutes(100);Clostridia(100);Clostridiales(100);Ruminococcaceae(100);Clostridium_III(100); | RII(2t)upUni | 617 |
| Otu061 | Bacteria(100);Firmicutes(100);Clostridia(100);Clostridiales(100);Ruminococcaceae(100);Clostridium_III(100); | RII(3t)downUni | 314 |
| Otu061 | Bacteria(100);Firmicutes(100);Clostridia(100);Clostridiales(100);Ruminococcaceae(100);Clostridium_III(100); | RII(3t)upUni | 500 |
| Otu061 | Bacteria(100);Firmicutes(100);Clostridia(100);Clostridiales(100);Ruminococcaceae(100);Clostridium_III(100); | S1Uni | 278 |
| Otu061 | Bacteria(100);Firmicutes(100);Clostridia(100);Clostridiales(100);Ruminococcaceae(100);Clostridium_III(100); | S2Uni | 319 |
| Otu062 | Bacteria(100);"Tenericutes"(100);Mollicutes(100);Acholeplasmatales(100);Acholeplasmataceae(100);Acholeplasma(100); | RI(1t)downUni | 16 |
| Otu062 | Bacteria(100);"Tenericutes"(100);Mollicutes(100);Acholeplasmatales(100);Acholeplasmataceae(100);Acholeplasma(100); | RI(1t)upUni | 4 |
| Otu062 | Bacteria(100);"Tenericutes"(100);Mollicutes(100);Acholeplasmatales(100);Acholeplasmataceae(100);Acholeplasma(100); | RI(2t)downUni | 580 |
| Otu062 | Bacteria(100);"Tenericutes"(100);Mollicutes(100);Acholeplasmatales(100);Acholeplasmataceae(100);Acholeplasma(100); | RI(2t)upUni | 1521 |
| Otu062 | Bacteria(100);"Tenericutes"(100);Mollicutes(100);Acholeplasmatales(100);Acholeplasmataceae(100);Acholeplasma(100); | RI(3t)downUni | 629 |
| Otu062 | Bacteria(100);"Tenericutes"(100);Mollicutes(100);Acholeplasmatales(100);Acholeplasmataceae(100);Acholeplasma(100); | RI(3t)upUni | 224 |
| Otu062 | Bacteria(100);"Tenericutes"(100);Mollicutes(100);Acholeplasmatales(100);Acholeplasmataceae(100);Acholeplasma(100); | RII(1t)downUni | 8 |
| Otu062 | Bacteria(100);"Tenericutes"(100);Mollicutes(100);Acholeplasmatales(100);Acholeplasmataceae(100);Acholeplasma(100); | RII(1t)upUni | 50 |
| Otu062 | Bacteria(100);"Tenericutes"(100);Mollicutes(100);Acholeplasmatales(100);Acholeplasmataceae(100);Acholeplasma(100); | RII(2t)downUni | 898 |
| Otu062 | Bacteria(100);"Tenericutes"(100);Mollicutes(100);Acholeplasmatales(100);Acholeplasmataceae(100);Acholeplasma(100); | RII(2t)upUni | 3607 |
| Otu062 | Bacteria(100);"Tenericutes"(100);Mollicutes(100);Acholeplasmatales(100);Acholeplasmataceae(100);Acholeplasma(100); | RII(3t)downUni | 786 |
| Otu062 | Bacteria(100);"Tenericutes"(100);Mollicutes(100);Acholeplasmatales(100);Acholeplasmataceae(100);Acholeplasma(100); | RII(3t)upUni | 611 |
| Otu062 | Bacteria(100);"Tenericutes"(100);Mollicutes(100);Acholeplasmatales(100);Acholeplasmataceae(100);Acholeplasma(100); | S1Uni | 11 |
| Otu062 | Bacteria(100);"Tenericutes"(100);Mollicutes(100);Acholeplasmatales(100);Acholeplasmataceae(100);Acholeplasma(100); | S2Uni | 41 |
| Otu064 | Bacteria(100);Firmicutes(100);Clostridia(100);Clostridiales(100);Lachnospiraceae(100);unclassified(100); | RI(1t)downUni | 53 |
| Otu064 | Bacteria(100);Firmicutes(100);Clostridia(100);Clostridiales(100);Lachnospiraceae(100);unclassified(100); | RI(1t)upUni | 31 |
| Otu064 | Bacteria(100);Firmicutes(100);Clostridia(100);Clostridiales(100);Lachnospiraceae(100);unclassified(100); | RI(2t)downUni | 155 |
| Otu064 | Bacteria(100);Firmicutes(100);Clostridia(100);Clostridiales(100);Lachnospiraceae(100);unclassified(100); | RI(2t)upUni | 1033 |
| Otu064 | Bacteria(100);Firmicutes(100);Clostridia(100);Clostridiales(100);Lachnospiraceae(100);unclassified(100); | RI(3t)downUni | 237 |
| Otu064 | Bacteria(100);Firmicutes(100);Clostridia(100);Clostridiales(100);Lachnospiraceae(100);unclassified(100); | RI(3t)upUni | 156 |
| Otu064 | Bacteria(100);Firmicutes(100);Clostridia(100);Clostridiales(100);Lachnospiraceae(100);unclassified(100); | RII(1t)downUni | 278 |
| Otu064 | Bacteria(100);Firmicutes(100);Clostridia(100);Clostridiales(100);Lachnospiraceae(100);unclassified(100); | RII(1t)upUni | 76 |
| Otu064 | Bacteria(100);Firmicutes(100);Clostridia(100);Clostridiales(100);Lachnospiraceae(100);unclassified(100); | RII(2t)downUni | 133 |
| Otu064 | Bacteria(100);Firmicutes(100);Clostridia(100);Clostridiales(100);Lachnospiraceae(100);unclassified(100); | RII(2t)upUni | 597 |
| Otu064 | Bacteria(100);Firmicutes(100);Clostridia(100);Clostridiales(100);Lachnospiraceae(100);unclassified(100); | RII(3t)downUni | 267 |
| Otu064 | Bacteria(100);Firmicutes(100);Clostridia(100);Clostridiales(100);Lachnospiraceae(100);unclassified(100); | RII(3t)upUni | 361 |
| Otu064 | Bacteria(100);Firmicutes(100);Clostridia(100);Clostridiales(100);Lachnospiraceae(100);unclassified(100); | S1Uni | 23 |
| Otu064 | Bacteria(100);Firmicutes(100);Clostridia(100);Clostridiales(100);Lachnospiraceae(100);unclassified(100); | S2Uni | 42 |
| Otu065 | Bacteria(100);"Actinobacteria"(100);Actinobacteria(100);Actinomycetales(100);unclassified(100);unclassified(100); | RI(1t)downUni | 6 |
| Otu065 | Bacteria(100);"Actinobacteria"(100);Actinobacteria(100);Actinomycetales(100);unclassified(100);unclassified(100); | RI(1t)upUni | 33 |
| Otu065 | Bacteria(100);"Actinobacteria"(100);Actinobacteria(100);Actinomycetales(100);unclassified(100);unclassified(100); | RI(2t)downUni | 5 |
| Otu065 | Bacteria(100);"Actinobacteria"(100);Actinobacteria(100);Actinomycetales(100);unclassified(100);unclassified(100); | RI(2t)upUni | 45 |
| Otu065 | Bacteria(100);"Actinobacteria"(100);Actinobacteria(100);Actinomycetales(100);unclassified(100);unclassified(100); | RI(3t)downUni | 1 |
| Otu065 | Bacteria(100);"Actinobacteria"(100);Actinobacteria(100);Actinomycetales(100);unclassified(100);unclassified(100); | RI(3t)upUni | 16 |
| Otu065 | Bacteria(100);"Actinobacteria"(100);Actinobacteria(100);Actinomycetales(100);unclassified(100);unclassified(100); | RII(1t)downUni | 36 |
| Otu065 | Bacteria(100);"Actinobacteria"(100);Actinobacteria(100);Actinomycetales(100);unclassified(100);unclassified(100); | RII(1t)upUni | 37 |
| Otu065 | Bacteria(100);"Actinobacteria"(100);Actinobacteria(100);Actinomycetales(100);unclassified(100);unclassified(100); | RII(2t)downUni | 5 |
| Otu065 | Bacteria(100);"Actinobacteria"(100);Actinobacteria(100);Actinomycetales(100);unclassified(100);unclassified(100); | RII(2t)upUni | 20 |
| Otu065 | Bacteria(100);"Actinobacteria"(100);Actinobacteria(100);Actinomycetales(100);unclassified(100);unclassified(100); | RII(3t)downUni | 19 |
| Otu065 | Bacteria(100);"Actinobacteria"(100);Actinobacteria(100);Actinomycetales(100);unclassified(100);unclassified(100); | RII(3t)upUni | 29 |
| Otu065 | Bacteria(100);"Actinobacteria"(100);Actinobacteria(100);Actinomycetales(100);unclassified(100);unclassified(100); | S1Uni | 1416 |
| Otu065 | Bacteria(100);"Actinobacteria"(100);Actinobacteria(100);Actinomycetales(100);unclassified(100);unclassified(100); | S2Uni | 1626 |
| Otu066 | Bacteria(100);"Proteobacteria"(100);Deltaproteobacteria(100);Syntrophobacterales(100);Syntrophaceae(100);Syntrophus(100); | RI(1t)downUni | 140 |
| Otu066 | Bacteria(100);"Proteobacteria"(100);Deltaproteobacteria(100);Syntrophobacterales(100);Syntrophaceae(100);Syntrophus(100); | RI(1t)upUni | 45 |
| Otu066 | Bacteria(100);"Proteobacteria"(100);Deltaproteobacteria(100);Syntrophobacterales(100);Syntrophaceae(100);Syntrophus(100); | RI(2t)downUni | 80 |
| Otu066 | Bacteria(100);"Proteobacteria"(100);Deltaproteobacteria(100);Syntrophobacterales(100);Syntrophaceae(100);Syntrophus(100); | RI(2t)upUni | 460 |
| Otu066 | Bacteria(100);"Proteobacteria"(100);Deltaproteobacteria(100);Syntrophobacterales(100);Syntrophaceae(100);Syntrophus(100); | RI(3t)downUni | 22 |
| Otu066 | Bacteria(100);"Proteobacteria"(100);Deltaproteobacteria(100);Syntrophobacterales(100);Syntrophaceae(100);Syntrophus(100); | RI(3t)upUni | 8 |
| Otu066 | Bacteria(100);"Proteobacteria"(100);Deltaproteobacteria(100);Syntrophobacterales(100);Syntrophaceae(100);Syntrophus(100); | RII(1t)downUni | 367 |
| Otu066 | Bacteria(100);"Proteobacteria"(100);Deltaproteobacteria(100);Syntrophobacterales(100);Syntrophaceae(100);Syntrophus(100); | RII(1t)upUni | 121 |
| Otu066 | Bacteria(100);"Proteobacteria"(100);Deltaproteobacteria(100);Syntrophobacterales(100);Syntrophaceae(100);Syntrophus(100); | RII(2t)downUni | 37 |
| Otu066 | Bacteria(100);"Proteobacteria"(100);Deltaproteobacteria(100);Syntrophobacterales(100);Syntrophaceae(100);Syntrophus(100); | RII(2t)upUni | 125 |
| Otu066 | Bacteria(100);"Proteobacteria"(100);Deltaproteobacteria(100);Syntrophobacterales(100);Syntrophaceae(100);Syntrophus(100); | RII(3t)downUni | 21 |
| Otu066 | Bacteria(100);"Proteobacteria"(100);Deltaproteobacteria(100);Syntrophobacterales(100);Syntrophaceae(100);Syntrophus(100); | RII(3t)upUni | 3 |
| Otu066 | Bacteria(100);"Proteobacteria"(100);Deltaproteobacteria(100);Syntrophobacterales(100);Syntrophaceae(100);Syntrophus(100); | S1Uni | 2015 |
| Otu066 | Bacteria(100);"Proteobacteria"(100);Deltaproteobacteria(100);Syntrophobacterales(100);Syntrophaceae(100);Syntrophus(100); | S2Uni | 2031 |
| Otu071 | Bacteria(100);Firmicutes(100);Clostridia(100);Clostridiales(100);Clostridiaceae_1(100);Proteiniclasticum(100); | RI(1t)downUni | 1 |
| Otu071 | Bacteria(100);Firmicutes(100);Clostridia(100);Clostridiales(100);Clostridiaceae_1(100);Proteiniclasticum(100); | RI(1t)upUni | 4 |
| Otu071 | Bacteria(100);Firmicutes(100);Clostridia(100);Clostridiales(100);Clostridiaceae_1(100);Proteiniclasticum(100); | RI(2t)downUni | 311 |
| Otu071 | Bacteria(100);Firmicutes(100);Clostridia(100);Clostridiales(100);Clostridiaceae_1(100);Proteiniclasticum(100); | RI(2t)upUni | 1945 |
| Otu071 | Bacteria(100);Firmicutes(100);Clostridia(100);Clostridiales(100);Clostridiaceae_1(100);Proteiniclasticum(100); | RI(3t)downUni | 80 |
| Otu071 | Bacteria(100);Firmicutes(100);Clostridia(100);Clostridiales(100);Clostridiaceae_1(100);Proteiniclasticum(100); | RI(3t)upUni | 40 |
| Otu071 | Bacteria(100);Firmicutes(100);Clostridia(100);Clostridiales(100);Clostridiaceae_1(100);Proteiniclasticum(100); | RII(1t)downUni | 14 |
| Otu071 | Bacteria(100);Firmicutes(100);Clostridia(100);Clostridiales(100);Clostridiaceae_1(100);Proteiniclasticum(100); | RII(1t)upUni | 54 |
| Otu071 | Bacteria(100);Firmicutes(100);Clostridia(100);Clostridiales(100);Clostridiaceae_1(100);Proteiniclasticum(100); | RII(2t)downUni | 320 |
| Otu071 | Bacteria(100);Firmicutes(100);Clostridia(100);Clostridiales(100);Clostridiaceae_1(100);Proteiniclasticum(100); | RII(2t)upUni | 1223 |
| Otu071 | Bacteria(100);Firmicutes(100);Clostridia(100);Clostridiales(100);Clostridiaceae_1(100);Proteiniclasticum(100); | RII(3t)downUni | 229 |
| Otu071 | Bacteria(100);Firmicutes(100);Clostridia(100);Clostridiales(100);Clostridiaceae_1(100);Proteiniclasticum(100); | RII(3t)upUni | 139 |
| Otu071 | Bacteria(100);Firmicutes(100);Clostridia(100);Clostridiales(100);Clostridiaceae_1(100);Proteiniclasticum(100); | S1Uni | 26 |
| Otu071 | Bacteria(100);Firmicutes(100);Clostridia(100);Clostridiales(100);Clostridiaceae_1(100);Proteiniclasticum(100); | S2Uni | 16 |
| Otu073 | Bacteria(100);Firmicutes(100);Bacilli(100);Lactobacillales(100);Lactobacillaceae(100);Lactobacillus(100); | RI(1t)downUni | 84 |
| Otu073 | Bacteria(100);Firmicutes(100);Bacilli(100);Lactobacillales(100);Lactobacillaceae(100);Lactobacillus(100); | RI(1t)upUni | 966 |
| Otu073 | Bacteria(100);Firmicutes(100);Bacilli(100);Lactobacillales(100);Lactobacillaceae(100);Lactobacillus(100); | RI(2t)downUni | 386 |
| Otu073 | Bacteria(100);Firmicutes(100);Bacilli(100);Lactobacillales(100);Lactobacillaceae(100);Lactobacillus(100); | RI(2t)upUni | 2783 |
| Otu073 | Bacteria(100);Firmicutes(100);Bacilli(100);Lactobacillales(100);Lactobacillaceae(100);Lactobacillus(100); | RI(3t)downUni | 322 |
| Otu073 | Bacteria(100);Firmicutes(100);Bacilli(100);Lactobacillales(100);Lactobacillaceae(100);Lactobacillus(100); | RI(3t)upUni | 308 |
| Otu073 | Bacteria(100);Firmicutes(100);Bacilli(100);Lactobacillales(100);Lactobacillaceae(100);Lactobacillus(100); | RII(1t)downUni | 341 |
| Otu073 | Bacteria(100);Firmicutes(100);Bacilli(100);Lactobacillales(100);Lactobacillaceae(100);Lactobacillus(100); | RII(1t)upUni | 398 |
| Otu073 | Bacteria(100);Firmicutes(100);Bacilli(100);Lactobacillales(100);Lactobacillaceae(100);Lactobacillus(100); | RII(2t)downUni | 553 |
| Otu073 | Bacteria(100);Firmicutes(100);Bacilli(100);Lactobacillales(100);Lactobacillaceae(100);Lactobacillus(100); | RII(2t)upUni | 2271 |
| Otu073 | Bacteria(100);Firmicutes(100);Bacilli(100);Lactobacillales(100);Lactobacillaceae(100);Lactobacillus(100); | RII(3t)downUni | 1004 |
| Otu073 | Bacteria(100);Firmicutes(100);Bacilli(100);Lactobacillales(100);Lactobacillaceae(100);Lactobacillus(100); | RII(3t)upUni | 3127 |
| Otu073 | Bacteria(100);Firmicutes(100);Bacilli(100);Lactobacillales(100);Lactobacillaceae(100);Lactobacillus(100); | S1Uni | 1285 |
| Otu073 | Bacteria(100);Firmicutes(100);Bacilli(100);Lactobacillales(100);Lactobacillaceae(100);Lactobacillus(100); | S2Uni | 1449 |
| Otu074 | Bacteria(100);"Proteobacteria"(100);Deltaproteobacteria(100);Syntrophobacterales(100);Syntrophobacteraceae(100);unclassified(100); | RI(1t)downUni | 6 |
| Otu074 | Bacteria(100);"Proteobacteria"(100);Deltaproteobacteria(100);Syntrophobacterales(100);Syntrophobacteraceae(100);unclassified(100); | RI(1t)upUni | 11 |
| Otu074 | Bacteria(100);"Proteobacteria"(100);Deltaproteobacteria(100);Syntrophobacterales(100);Syntrophobacteraceae(100);unclassified(100); | RI(2t)downUni | 8 |
| Otu074 | Bacteria(100);"Proteobacteria"(100);Deltaproteobacteria(100);Syntrophobacterales(100);Syntrophobacteraceae(100);unclassified(100); | RI(2t)upUni | 69 |
| Otu074 | Bacteria(100);"Proteobacteria"(100);Deltaproteobacteria(100);Syntrophobacterales(100);Syntrophobacteraceae(100);unclassified(100); | RI(3t)downUni | 6 |
| Otu074 | Bacteria(100);"Proteobacteria"(100);Deltaproteobacteria(100);Syntrophobacterales(100);Syntrophobacteraceae(100);unclassified(100); | RI(3t)upUni | 1 |
| Otu074 | Bacteria(100);"Proteobacteria"(100);Deltaproteobacteria(100);Syntrophobacterales(100);Syntrophobacteraceae(100);unclassified(100); | RII(1t)downUni | 86 |
| Otu074 | Bacteria(100);"Proteobacteria"(100);Deltaproteobacteria(100);Syntrophobacterales(100);Syntrophobacteraceae(100);unclassified(100); | RII(1t)upUni | 41 |
| Otu074 | Bacteria(100);"Proteobacteria"(100);Deltaproteobacteria(100);Syntrophobacterales(100);Syntrophobacteraceae(100);unclassified(100); | RII(2t)downUni | 10 |
| Otu074 | Bacteria(100);"Proteobacteria"(100);Deltaproteobacteria(100);Syntrophobacterales(100);Syntrophobacteraceae(100);unclassified(100); | RII(2t)upUni | 27 |
| Otu074 | Bacteria(100);"Proteobacteria"(100);Deltaproteobacteria(100);Syntrophobacterales(100);Syntrophobacteraceae(100);unclassified(100); | RII(3t)downUni | 10 |
| Otu074 | Bacteria(100);"Proteobacteria"(100);Deltaproteobacteria(100);Syntrophobacterales(100);Syntrophobacteraceae(100);unclassified(100); | RII(3t)upUni | 1 |
| Otu074 | Bacteria(100);"Proteobacteria"(100);Deltaproteobacteria(100);Syntrophobacterales(100);Syntrophobacteraceae(100);unclassified(100); | S1Uni | 356 |
| Otu074 | Bacteria(100);"Proteobacteria"(100);Deltaproteobacteria(100);Syntrophobacterales(100);Syntrophobacteraceae(100);unclassified(100); | S2Uni | 459 |
| Otu076 | Bacteria(100);"Proteobacteria"(100);Alphaproteobacteria(100);unclassified(100);unclassified(100);unclassified(100); | RI(1t)downUni | 22 |
| Otu076 | Bacteria(100);"Proteobacteria"(100);Alphaproteobacteria(100);unclassified(100);unclassified(100);unclassified(100); | RI(1t)upUni | 49 |
| Otu076 | Bacteria(100);"Proteobacteria"(100);Alphaproteobacteria(100);unclassified(100);unclassified(100);unclassified(100); | RI(2t)downUni | 4 |
| Otu076 | Bacteria(100);"Proteobacteria"(100);Alphaproteobacteria(100);unclassified(100);unclassified(100);unclassified(100); | RI(2t)upUni | 51 |
| Otu076 | Bacteria(100);"Proteobacteria"(100);Alphaproteobacteria(100);unclassified(100);unclassified(100);unclassified(100); | RI(3t)downUni | 10 |
| Otu076 | Bacteria(100);"Proteobacteria"(100);Alphaproteobacteria(100);unclassified(100);unclassified(100);unclassified(100); | RI(3t)upUni | 3 |
| Otu076 | Bacteria(100);"Proteobacteria"(100);Alphaproteobacteria(100);unclassified(100);unclassified(100);unclassified(100); | RII(1t)downUni | 86 |
| Otu076 | Bacteria(100);"Proteobacteria"(100);Alphaproteobacteria(100);unclassified(100);unclassified(100);unclassified(100); | RII(1t)upUni | 58 |
| Otu076 | Bacteria(100);"Proteobacteria"(100);Alphaproteobacteria(100);unclassified(100);unclassified(100);unclassified(100); | RII(2t)downUni | 6 |
| Otu076 | Bacteria(100);"Proteobacteria"(100);Alphaproteobacteria(100);unclassified(100);unclassified(100);unclassified(100); | RII(2t)upUni | 41 |
| Otu076 | Bacteria(100);"Proteobacteria"(100);Alphaproteobacteria(100);unclassified(100);unclassified(100);unclassified(100); | RII(3t)downUni | 9 |
| Otu076 | Bacteria(100);"Proteobacteria"(100);Alphaproteobacteria(100);unclassified(100);unclassified(100);unclassified(100); | RII(3t)upUni | 2 |
| Otu076 | Bacteria(100);"Proteobacteria"(100);Alphaproteobacteria(100);unclassified(100);unclassified(100);unclassified(100); | S1Uni | 642 |
| Otu076 | Bacteria(100);"Proteobacteria"(100);Alphaproteobacteria(100);unclassified(100);unclassified(100);unclassified(100); | S2Uni | 688 |
| Otu081 | Bacteria(100);"Proteobacteria"(100);Alphaproteobacteria(100);Rhodobacterales(100);Rhodobacteraceae(100);unclassified(100); | RI(1t)downUni | 10 |
| Otu081 | Bacteria(100);"Proteobacteria"(100);Alphaproteobacteria(100);Rhodobacterales(100);Rhodobacteraceae(100);unclassified(100); | RI(1t)upUni | 271 |
| Otu081 | Bacteria(100);"Proteobacteria"(100);Alphaproteobacteria(100);Rhodobacterales(100);Rhodobacteraceae(100);unclassified(100); | RI(2t)downUni | 6 |
| Otu081 | Bacteria(100);"Proteobacteria"(100);Alphaproteobacteria(100);Rhodobacterales(100);Rhodobacteraceae(100);unclassified(100); | RI(2t)upUni | 91 |
| Otu081 | Bacteria(100);"Proteobacteria"(100);Alphaproteobacteria(100);Rhodobacterales(100);Rhodobacteraceae(100);unclassified(100); | RI(3t)downUni | 5 |
| Otu081 | Bacteria(100);"Proteobacteria"(100);Alphaproteobacteria(100);Rhodobacterales(100);Rhodobacteraceae(100);unclassified(100); | RI(3t)upUni | 11 |
| Otu081 | Bacteria(100);"Proteobacteria"(100);Alphaproteobacteria(100);Rhodobacterales(100);Rhodobacteraceae(100);unclassified(100); | RII(1t)downUni | 62 |
| Otu081 | Bacteria(100);"Proteobacteria"(100);Alphaproteobacteria(100);Rhodobacterales(100);Rhodobacteraceae(100);unclassified(100); | RII(1t)upUni | 132 |
| Otu081 | Bacteria(100);"Proteobacteria"(100);Alphaproteobacteria(100);Rhodobacterales(100);Rhodobacteraceae(100);unclassified(100); | RII(2t)downUni | 16 |
| Otu081 | Bacteria(100);"Proteobacteria"(100);Alphaproteobacteria(100);Rhodobacterales(100);Rhodobacteraceae(100);unclassified(100); | RII(2t)upUni | 27 |
| Otu081 | Bacteria(100);"Proteobacteria"(100);Alphaproteobacteria(100);Rhodobacterales(100);Rhodobacteraceae(100);unclassified(100); | RII(3t)downUni | 39 |
| Otu081 | Bacteria(100);"Proteobacteria"(100);Alphaproteobacteria(100);Rhodobacterales(100);Rhodobacteraceae(100);unclassified(100); | RII(3t)upUni | 42 |
| Otu081 | Bacteria(100);"Proteobacteria"(100);Alphaproteobacteria(100);Rhodobacterales(100);Rhodobacteraceae(100);unclassified(100); | S1Uni | 572 |
| Otu081 | Bacteria(100);"Proteobacteria"(100);Alphaproteobacteria(100);Rhodobacterales(100);Rhodobacteraceae(100);unclassified(100); | S2Uni | 642 |
| Otu084 | Bacteria(100);OD1(100);OD1_class_incertae_sedis(100);OD1_order_incertae_sedis(100);OD1_family_incertae_sedis(100);OD1_genus_incertae_sedis(100); | RI(1t)downUni | 19 |
| Otu084 | Bacteria(100);OD1(100);OD1_class_incertae_sedis(100);OD1_order_incertae_sedis(100);OD1_family_incertae_sedis(100);OD1_genus_incertae_sedis(100); | RI(1t)upUni | 65 |
| Otu084 | Bacteria(100);OD1(100);OD1_class_incertae_sedis(100);OD1_order_incertae_sedis(100);OD1_family_incertae_sedis(100);OD1_genus_incertae_sedis(100); | RI(2t)downUni | 64 |
| Otu084 | Bacteria(100);OD1(100);OD1_class_incertae_sedis(100);OD1_order_incertae_sedis(100);OD1_family_incertae_sedis(100);OD1_genus_incertae_sedis(100); | RI(2t)upUni | 1037 |
| Otu084 | Bacteria(100);OD1(100);OD1_class_incertae_sedis(100);OD1_order_incertae_sedis(100);OD1_family_incertae_sedis(100);OD1_genus_incertae_sedis(100); | RI(3t)downUni | 146 |
| Otu084 | Bacteria(100);OD1(100);OD1_class_incertae_sedis(100);OD1_order_incertae_sedis(100);OD1_family_incertae_sedis(100);OD1_genus_incertae_sedis(100); | RI(3t)upUni | 227 |
| Otu084 | Bacteria(100);OD1(100);OD1_class_incertae_sedis(100);OD1_order_incertae_sedis(100);OD1_family_incertae_sedis(100);OD1_genus_incertae_sedis(100); | RII(1t)downUni | 79 |
| Otu084 | Bacteria(100);OD1(100);OD1_class_incertae_sedis(100);OD1_order_incertae_sedis(100);OD1_family_incertae_sedis(100);OD1_genus_incertae_sedis(100); | RII(1t)upUni | 61 |
| Otu084 | Bacteria(100);OD1(100);OD1_class_incertae_sedis(100);OD1_order_incertae_sedis(100);OD1_family_incertae_sedis(100);OD1_genus_incertae_sedis(100); | RII(2t)downUni | 854 |
| Otu084 | Bacteria(100);OD1(100);OD1_class_incertae_sedis(100);OD1_order_incertae_sedis(100);OD1_family_incertae_sedis(100);OD1_genus_incertae_sedis(100); | RII(2t)upUni | 3644 |
| Otu084 | Bacteria(100);OD1(100);OD1_class_incertae_sedis(100);OD1_order_incertae_sedis(100);OD1_family_incertae_sedis(100);OD1_genus_incertae_sedis(100); | RII(3t)downUni | 436 |
| Otu084 | Bacteria(100);OD1(100);OD1_class_incertae_sedis(100);OD1_order_incertae_sedis(100);OD1_family_incertae_sedis(100);OD1_genus_incertae_sedis(100); | RII(3t)upUni | 1752 |
| Otu084 | Bacteria(100);OD1(100);OD1_class_incertae_sedis(100);OD1_order_incertae_sedis(100);OD1_family_incertae_sedis(100);OD1_genus_incertae_sedis(100); | S1Uni | 558 |
| Otu084 | Bacteria(100);OD1(100);OD1_class_incertae_sedis(100);OD1_order_incertae_sedis(100);OD1_family_incertae_sedis(100);OD1_genus_incertae_sedis(100); | S2Uni | 473 |
| Otu088 | Bacteria(100);"Proteobacteria"(100);Gammaproteobacteria(100);Xanthomonadales(100);Xanthomonadaceae(100);unclassified(100); | RI(1t)downUni | 41 |
| Otu088 | Bacteria(100);"Proteobacteria"(100);Gammaproteobacteria(100);Xanthomonadales(100);Xanthomonadaceae(100);unclassified(100); | RI(1t)upUni | 146 |
| Otu088 | Bacteria(100);"Proteobacteria"(100);Gammaproteobacteria(100);Xanthomonadales(100);Xanthomonadaceae(100);unclassified(100); | RI(2t)downUni | 7 |
| Otu088 | Bacteria(100);"Proteobacteria"(100);Gammaproteobacteria(100);Xanthomonadales(100);Xanthomonadaceae(100);unclassified(100); | RI(2t)upUni | 198 |
| Otu088 | Bacteria(100);"Proteobacteria"(100);Gammaproteobacteria(100);Xanthomonadales(100);Xanthomonadaceae(100);unclassified(100); | RI(3t)downUni | 2 |
| Otu088 | Bacteria(100);"Proteobacteria"(100);Gammaproteobacteria(100);Xanthomonadales(100);Xanthomonadaceae(100);unclassified(100); | RI(3t)upUni | 7 |
| Otu088 | Bacteria(100);"Proteobacteria"(100);Gammaproteobacteria(100);Xanthomonadales(100);Xanthomonadaceae(100);unclassified(100); | RII(1t)downUni | 145 |
| Otu088 | Bacteria(100);"Proteobacteria"(100);Gammaproteobacteria(100);Xanthomonadales(100);Xanthomonadaceae(100);unclassified(100); | RII(1t)upUni | 90 |
| Otu088 | Bacteria(100);"Proteobacteria"(100);Gammaproteobacteria(100);Xanthomonadales(100);Xanthomonadaceae(100);unclassified(100); | RII(2t)downUni | 10 |
| Otu088 | Bacteria(100);"Proteobacteria"(100);Gammaproteobacteria(100);Xanthomonadales(100);Xanthomonadaceae(100);unclassified(100); | RII(2t)upUni | 96 |
| Otu088 | Bacteria(100);"Proteobacteria"(100);Gammaproteobacteria(100);Xanthomonadales(100);Xanthomonadaceae(100);unclassified(100); | RII(3t)downUni | 11 |
| Otu088 | Bacteria(100);"Proteobacteria"(100);Gammaproteobacteria(100);Xanthomonadales(100);Xanthomonadaceae(100);unclassified(100); | RII(3t)upUni | 19 |
| Otu088 | Bacteria(100);"Proteobacteria"(100);Gammaproteobacteria(100);Xanthomonadales(100);Xanthomonadaceae(100);unclassified(100); | S1Uni | 498 |
| Otu088 | Bacteria(100);"Proteobacteria"(100);Gammaproteobacteria(100);Xanthomonadales(100);Xanthomonadaceae(100);unclassified(100); | S2Uni | 626 |
| Otu090 | Bacteria(100);"Proteobacteria"(100);Betaproteobacteria(100);Rhodocyclales(100);Rhodocyclaceae(100);Thauera(100); | RI(1t)downUni | 2 |
| Otu090 | Bacteria(100);"Proteobacteria"(100);Betaproteobacteria(100);Rhodocyclales(100);Rhodocyclaceae(100);Thauera(100); | RI(1t)upUni | 3 |
| Otu090 | Bacteria(100);"Proteobacteria"(100);Betaproteobacteria(100);Rhodocyclales(100);Rhodocyclaceae(100);Thauera(100); | RI(2t)downUni | 106 |
| Otu090 | Bacteria(100);"Proteobacteria"(100);Betaproteobacteria(100);Rhodocyclales(100);Rhodocyclaceae(100);Thauera(100); | RI(2t)upUni | 543 |
| Otu090 | Bacteria(100);"Proteobacteria"(100);Betaproteobacteria(100);Rhodocyclales(100);Rhodocyclaceae(100);Thauera(100); | RI(3t)downUni | 52 |
| Otu090 | Bacteria(100);"Proteobacteria"(100);Betaproteobacteria(100);Rhodocyclales(100);Rhodocyclaceae(100);Thauera(100); | RI(3t)upUni | 32 |
| Otu090 | Bacteria(100);"Proteobacteria"(100);Betaproteobacteria(100);Rhodocyclales(100);Rhodocyclaceae(100);Thauera(100); | RII(1t)downUni | 7 |
| Otu090 | Bacteria(100);"Proteobacteria"(100);Betaproteobacteria(100);Rhodocyclales(100);Rhodocyclaceae(100);Thauera(100); | RII(1t)upUni | 5 |
| Otu090 | Bacteria(100);"Proteobacteria"(100);Betaproteobacteria(100);Rhodocyclales(100);Rhodocyclaceae(100);Thauera(100); | RII(2t)downUni | 150 |
| Otu090 | Bacteria(100);"Proteobacteria"(100);Betaproteobacteria(100);Rhodocyclales(100);Rhodocyclaceae(100);Thauera(100); | RII(2t)upUni | 627 |
| Otu090 | Bacteria(100);"Proteobacteria"(100);Betaproteobacteria(100);Rhodocyclales(100);Rhodocyclaceae(100);Thauera(100); | RII(3t)downUni | 191 |
| Otu090 | Bacteria(100);"Proteobacteria"(100);Betaproteobacteria(100);Rhodocyclales(100);Rhodocyclaceae(100);Thauera(100); | RII(3t)upUni | 132 |
| Otu090 | Bacteria(100);"Proteobacteria"(100);Betaproteobacteria(100);Rhodocyclales(100);Rhodocyclaceae(100);Thauera(100); | S1Uni | 61 |
| Otu090 | Bacteria(100);"Proteobacteria"(100);Betaproteobacteria(100);Rhodocyclales(100);Rhodocyclaceae(100);Thauera(100); | S2Uni | 70 |
| Otu091 | Bacteria(100);"Proteobacteria"(100);Gammaproteobacteria(100);Xanthomonadales(100);Xanthomonadaceae(100);Stenotrophomonas(100); | RI(1t)downUni | 41 |
| Otu091 | Bacteria(100);"Proteobacteria"(100);Gammaproteobacteria(100);Xanthomonadales(100);Xanthomonadaceae(100);Stenotrophomonas(100); | RI(1t)upUni | 48 |
| Otu091 | Bacteria(100);"Proteobacteria"(100);Gammaproteobacteria(100);Xanthomonadales(100);Xanthomonadaceae(100);Stenotrophomonas(100); | RI(2t)downUni | 7 |
| Otu091 | Bacteria(100);"Proteobacteria"(100);Gammaproteobacteria(100);Xanthomonadales(100);Xanthomonadaceae(100);Stenotrophomonas(100); | RI(2t)upUni | 93 |
| Otu091 | Bacteria(100);"Proteobacteria"(100);Gammaproteobacteria(100);Xanthomonadales(100);Xanthomonadaceae(100);Stenotrophomonas(100); | RI(3t)downUni | 1 |
| Otu091 | Bacteria(100);"Proteobacteria"(100);Gammaproteobacteria(100);Xanthomonadales(100);Xanthomonadaceae(100);Stenotrophomonas(100); | RI(3t)upUni | 12 |
| Otu091 | Bacteria(100);"Proteobacteria"(100);Gammaproteobacteria(100);Xanthomonadales(100);Xanthomonadaceae(100);Stenotrophomonas(100); | RII(1t)downUni | 48 |
| Otu091 | Bacteria(100);"Proteobacteria"(100);Gammaproteobacteria(100);Xanthomonadales(100);Xanthomonadaceae(100);Stenotrophomonas(100); | RII(1t)upUni | 872 |
| Otu091 | Bacteria(100);"Proteobacteria"(100);Gammaproteobacteria(100);Xanthomonadales(100);Xanthomonadaceae(100);Stenotrophomonas(100); | RII(2t)downUni | 7 |
| Otu091 | Bacteria(100);"Proteobacteria"(100);Gammaproteobacteria(100);Xanthomonadales(100);Xanthomonadaceae(100);Stenotrophomonas(100); | RII(2t)upUni | 46 |
| Otu091 | Bacteria(100);"Proteobacteria"(100);Gammaproteobacteria(100);Xanthomonadales(100);Xanthomonadaceae(100);Stenotrophomonas(100); | RII(3t)downUni | 1 |
| Otu091 | Bacteria(100);"Proteobacteria"(100);Gammaproteobacteria(100);Xanthomonadales(100);Xanthomonadaceae(100);Stenotrophomonas(100); | RII(3t)upUni | 16 |
| Otu091 | Bacteria(100);"Proteobacteria"(100);Gammaproteobacteria(100);Xanthomonadales(100);Xanthomonadaceae(100);Stenotrophomonas(100); | S1Uni | 47 |
| Otu091 | Bacteria(100);"Proteobacteria"(100);Gammaproteobacteria(100);Xanthomonadales(100);Xanthomonadaceae(100);Stenotrophomonas(100); | S2Uni | 46 |
| Otu095 | Bacteria(100);"Proteobacteria"(100);Alphaproteobacteria(100);Rhizobiales(100);unclassified(100);unclassified(100); | RI(1t)downUni | 14 |
| Otu095 | Bacteria(100);"Proteobacteria"(100);Alphaproteobacteria(100);Rhizobiales(100);unclassified(100);unclassified(100); | RI(1t)upUni | 8 |
| Otu095 | Bacteria(100);"Proteobacteria"(100);Alphaproteobacteria(100);Rhizobiales(100);unclassified(100);unclassified(100); | RI(2t)downUni | 3 |
| Otu095 | Bacteria(100);"Proteobacteria"(100);Alphaproteobacteria(100);Rhizobiales(100);unclassified(100);unclassified(100); | RI(2t)upUni | 30 |
| Otu095 | Bacteria(100);"Proteobacteria"(100);Alphaproteobacteria(100);Rhizobiales(100);unclassified(100);unclassified(100); | RI(3t)downUni | 7 |
| Otu095 | Bacteria(100);"Proteobacteria"(100);Alphaproteobacteria(100);Rhizobiales(100);unclassified(100);unclassified(100); | RI(3t)upUni | 6 |
| Otu095 | Bacteria(100);"Proteobacteria"(100);Alphaproteobacteria(100);Rhizobiales(100);unclassified(100);unclassified(100); | RII(1t)downUni | 66 |
| Otu095 | Bacteria(100);"Proteobacteria"(100);Alphaproteobacteria(100);Rhizobiales(100);unclassified(100);unclassified(100); | RII(1t)upUni | 16 |
| Otu095 | Bacteria(100);"Proteobacteria"(100);Alphaproteobacteria(100);Rhizobiales(100);unclassified(100);unclassified(100); | RII(2t)downUni | 6 |
| Otu095 | Bacteria(100);"Proteobacteria"(100);Alphaproteobacteria(100);Rhizobiales(100);unclassified(100);unclassified(100); | RII(2t)upUni | 30 |
| Otu095 | Bacteria(100);"Proteobacteria"(100);Alphaproteobacteria(100);Rhizobiales(100);unclassified(100);unclassified(100); | RII(3t)downUni | 15 |
| Otu095 | Bacteria(100);"Proteobacteria"(100);Alphaproteobacteria(100);Rhizobiales(100);unclassified(100);unclassified(100); | RII(3t)upUni | 30 |
| Otu095 | Bacteria(100);"Proteobacteria"(100);Alphaproteobacteria(100);Rhizobiales(100);unclassified(100);unclassified(100); | S1Uni | 391 |
| Otu095 | Bacteria(100);"Proteobacteria"(100);Alphaproteobacteria(100);Rhizobiales(100);unclassified(100);unclassified(100); | S2Uni | 447 |
| Otu096 | Bacteria(100);Firmicutes(100);Clostridia(100);Clostridiales(100);Lachnospiraceae(100);Clostridium_XlVa(100); | RI(1t)downUni | 31 |
| Otu096 | Bacteria(100);Firmicutes(100);Clostridia(100);Clostridiales(100);Lachnospiraceae(100);Clostridium_XlVa(100); | RI(1t)upUni | 244 |
| Otu096 | Bacteria(100);Firmicutes(100);Clostridia(100);Clostridiales(100);Lachnospiraceae(100);Clostridium_XlVa(100); | RI(2t)downUni | 57 |
| Otu096 | Bacteria(100);Firmicutes(100);Clostridia(100);Clostridiales(100);Lachnospiraceae(100);Clostridium_XlVa(100); | RI(2t)upUni | 933 |
| Otu096 | Bacteria(100);Firmicutes(100);Clostridia(100);Clostridiales(100);Lachnospiraceae(100);Clostridium_XlVa(100); | RI(3t)downUni | 257 |
| Otu096 | Bacteria(100);Firmicutes(100);Clostridia(100);Clostridiales(100);Lachnospiraceae(100);Clostridium_XlVa(100); | RI(3t)upUni | 646 |
| Otu096 | Bacteria(100);Firmicutes(100);Clostridia(100);Clostridiales(100);Lachnospiraceae(100);Clostridium_XlVa(100); | RII(1t)downUni | 80 |
| Otu096 | Bacteria(100);Firmicutes(100);Clostridia(100);Clostridiales(100);Lachnospiraceae(100);Clostridium_XlVa(100); | RII(1t)upUni | 149 |
| Otu096 | Bacteria(100);Firmicutes(100);Clostridia(100);Clostridiales(100);Lachnospiraceae(100);Clostridium_XlVa(100); | RII(2t)downUni | 85 |
| Otu096 | Bacteria(100);Firmicutes(100);Clostridia(100);Clostridiales(100);Lachnospiraceae(100);Clostridium_XlVa(100); | RII(2t)upUni | 279 |
| Otu096 | Bacteria(100);Firmicutes(100);Clostridia(100);Clostridiales(100);Lachnospiraceae(100);Clostridium_XlVa(100); | RII(3t)downUni | 630 |
| Otu096 | Bacteria(100);Firmicutes(100);Clostridia(100);Clostridiales(100);Lachnospiraceae(100);Clostridium_XlVa(100); | RII(3t)upUni | 2342 |
| Otu096 | Bacteria(100);Firmicutes(100);Clostridia(100);Clostridiales(100);Lachnospiraceae(100);Clostridium_XlVa(100); | S1Uni | 9 |
| Otu096 | Bacteria(100);Firmicutes(100);Clostridia(100);Clostridiales(100);Lachnospiraceae(100);Clostridium_XlVa(100); | S2Uni | 5 |
| Otu097 | Bacteria(100);"Actinobacteria"(100);Actinobacteria(100);Actinomycetales(100);Mycobacteriaceae(100);Mycobacterium(100); | RI(1t)downUni | 47 |
| Otu097 | Bacteria(100);"Actinobacteria"(100);Actinobacteria(100);Actinomycetales(100);Mycobacteriaceae(100);Mycobacterium(100); | RI(1t)upUni | 56 |
| Otu097 | Bacteria(100);"Actinobacteria"(100);Actinobacteria(100);Actinomycetales(100);Mycobacteriaceae(100);Mycobacterium(100); | RI(2t)downUni | 7 |
| Otu097 | Bacteria(100);"Actinobacteria"(100);Actinobacteria(100);Actinomycetales(100);Mycobacteriaceae(100);Mycobacterium(100); | RI(2t)upUni | 202 |
| Otu097 | Bacteria(100);"Actinobacteria"(100);Actinobacteria(100);Actinomycetales(100);Mycobacteriaceae(100);Mycobacterium(100); | RI(3t)downUni | 6 |
| Otu097 | Bacteria(100);"Actinobacteria"(100);Actinobacteria(100);Actinomycetales(100);Mycobacteriaceae(100);Mycobacterium(100); | RI(3t)upUni | 20 |
| Otu097 | Bacteria(100);"Actinobacteria"(100);Actinobacteria(100);Actinomycetales(100);Mycobacteriaceae(100);Mycobacterium(100); | RII(1t)downUni | 140 |
| Otu097 | Bacteria(100);"Actinobacteria"(100);Actinobacteria(100);Actinomycetales(100);Mycobacteriaceae(100);Mycobacterium(100); | RII(1t)upUni | 60 |
| Otu097 | Bacteria(100);"Actinobacteria"(100);Actinobacteria(100);Actinomycetales(100);Mycobacteriaceae(100);Mycobacterium(100); | RII(2t)downUni | 22 |
| Otu097 | Bacteria(100);"Actinobacteria"(100);Actinobacteria(100);Actinomycetales(100);Mycobacteriaceae(100);Mycobacterium(100); | RII(2t)upUni | 49 |
| Otu097 | Bacteria(100);"Actinobacteria"(100);Actinobacteria(100);Actinomycetales(100);Mycobacteriaceae(100);Mycobacterium(100); | RII(3t)downUni | 22 |
| Otu097 | Bacteria(100);"Actinobacteria"(100);Actinobacteria(100);Actinomycetales(100);Mycobacteriaceae(100);Mycobacterium(100); | RII(3t)upUni | 25 |
| Otu097 | Bacteria(100);"Actinobacteria"(100);Actinobacteria(100);Actinomycetales(100);Mycobacteriaceae(100);Mycobacterium(100); | S1Uni | 964 |
| Otu097 | Bacteria(100);"Actinobacteria"(100);Actinobacteria(100);Actinomycetales(100);Mycobacteriaceae(100);Mycobacterium(100); | S2Uni | 1186 |
| Otu100 | Bacteria(100);"Proteobacteria"(100);Gammaproteobacteria(100);Aeromonadales(100);Aeromonadaceae(100);Aeromonas(100); | RI(1t)downUni | 116 |
| Otu100 | Bacteria(100);"Proteobacteria"(100);Gammaproteobacteria(100);Aeromonadales(100);Aeromonadaceae(100);Aeromonas(100); | RI(1t)upUni | 167 |
| Otu100 | Bacteria(100);"Proteobacteria"(100);Gammaproteobacteria(100);Aeromonadales(100);Aeromonadaceae(100);Aeromonas(100); | RI(2t)downUni | 38 |
| Otu100 | Bacteria(100);"Proteobacteria"(100);Gammaproteobacteria(100);Aeromonadales(100);Aeromonadaceae(100);Aeromonas(100); | RI(2t)upUni | 360 |
| Otu100 | Bacteria(100);"Proteobacteria"(100);Gammaproteobacteria(100);Aeromonadales(100);Aeromonadaceae(100);Aeromonas(100); | RI(3t)downUni | 125 |
| Otu100 | Bacteria(100);"Proteobacteria"(100);Gammaproteobacteria(100);Aeromonadales(100);Aeromonadaceae(100);Aeromonas(100); | RI(3t)upUni | 124 |
| Otu100 | Bacteria(100);"Proteobacteria"(100);Gammaproteobacteria(100);Aeromonadales(100);Aeromonadaceae(100);Aeromonas(100); | RII(1t)downUni | 280 |
| Otu100 | Bacteria(100);"Proteobacteria"(100);Gammaproteobacteria(100);Aeromonadales(100);Aeromonadaceae(100);Aeromonas(100); | RII(1t)upUni | 168 |
| Otu100 | Bacteria(100);"Proteobacteria"(100);Gammaproteobacteria(100);Aeromonadales(100);Aeromonadaceae(100);Aeromonas(100); | RII(2t)downUni | 29 |
| Otu100 | Bacteria(100);"Proteobacteria"(100);Gammaproteobacteria(100);Aeromonadales(100);Aeromonadaceae(100);Aeromonas(100); | RII(2t)upUni | 151 |
| Otu100 | Bacteria(100);"Proteobacteria"(100);Gammaproteobacteria(100);Aeromonadales(100);Aeromonadaceae(100);Aeromonas(100); | RII(3t)downUni | 81 |
| Otu100 | Bacteria(100);"Proteobacteria"(100);Gammaproteobacteria(100);Aeromonadales(100);Aeromonadaceae(100);Aeromonas(100); | RII(3t)upUni | 427 |
| Otu100 | Bacteria(100);"Proteobacteria"(100);Gammaproteobacteria(100);Aeromonadales(100);Aeromonadaceae(100);Aeromonas(100); | S1Uni | 137 |
| Otu100 | Bacteria(100);"Proteobacteria"(100);Gammaproteobacteria(100);Aeromonadales(100);Aeromonadaceae(100);Aeromonas(100); | S2Uni | 148 |
| Otu108 | Bacteria(100);Firmicutes(100);Bacilli(100);Lactobacillales(100);Enterococcaceae(100);Enterococcus(100); | RI(1t)downUni | 2 |
| Otu108 | Bacteria(100);Firmicutes(100);Bacilli(100);Lactobacillales(100);Enterococcaceae(100);Enterococcus(100); | RI(1t)upUni | 109 |
| Otu108 | Bacteria(100);Firmicutes(100);Bacilli(100);Lactobacillales(100);Enterococcaceae(100);Enterococcus(100); | RI(2t)downUni | 81 |
| Otu108 | Bacteria(100);Firmicutes(100);Bacilli(100);Lactobacillales(100);Enterococcaceae(100);Enterococcus(100); | RI(2t)upUni | 585 |
| Otu108 | Bacteria(100);Firmicutes(100);Bacilli(100);Lactobacillales(100);Enterococcaceae(100);Enterococcus(100); | RI(3t)downUni | 181 |
| Otu108 | Bacteria(100);Firmicutes(100);Bacilli(100);Lactobacillales(100);Enterococcaceae(100);Enterococcus(100); | RI(3t)upUni | 176 |
| Otu108 | Bacteria(100);Firmicutes(100);Bacilli(100);Lactobacillales(100);Enterococcaceae(100);Enterococcus(100); | RII(1t)downUni | 34 |
| Otu108 | Bacteria(100);Firmicutes(100);Bacilli(100);Lactobacillales(100);Enterococcaceae(100);Enterococcus(100); | RII(1t)upUni | 50 |
| Otu108 | Bacteria(100);Firmicutes(100);Bacilli(100);Lactobacillales(100);Enterococcaceae(100);Enterococcus(100); | RII(2t)downUni | 129 |
| Otu108 | Bacteria(100);Firmicutes(100);Bacilli(100);Lactobacillales(100);Enterococcaceae(100);Enterococcus(100); | RII(2t)upUni | 481 |
| Otu108 | Bacteria(100);Firmicutes(100);Bacilli(100);Lactobacillales(100);Enterococcaceae(100);Enterococcus(100); | RII(3t)downUni | 505 |
| Otu108 | Bacteria(100);Firmicutes(100);Bacilli(100);Lactobacillales(100);Enterococcaceae(100);Enterococcus(100); | RII(3t)upUni | 1123 |
| Otu108 | Bacteria(100);Firmicutes(100);Bacilli(100);Lactobacillales(100);Enterococcaceae(100);Enterococcus(100); | S1Uni | 6 |
| Otu108 | Bacteria(100);Firmicutes(100);Bacilli(100);Lactobacillales(100);Enterococcaceae(100);Enterococcus(100); | S2Uni | 8 |
| Otu126 | Bacteria(100);Firmicutes(100);Bacilli(100);Bacillales(100);unclassified(100);unclassified(100); | RI(1t)downUni | 17 |
| Otu126 | Bacteria(100);Firmicutes(100);Bacilli(100);Bacillales(100);unclassified(100);unclassified(100); | RI(1t)upUni | 10 |
| Otu126 | Bacteria(100);Firmicutes(100);Bacilli(100);Bacillales(100);unclassified(100);unclassified(100); | RI(2t)downUni | 3 |
| Otu126 | Bacteria(100);Firmicutes(100);Bacilli(100);Bacillales(100);unclassified(100);unclassified(100); | RI(2t)upUni | 11 |
| Otu126 | Bacteria(100);Firmicutes(100);Bacilli(100);Bacillales(100);unclassified(100);unclassified(100); | RI(3t)downUni | 1 |
| Otu126 | Bacteria(100);Firmicutes(100);Bacilli(100);Bacillales(100);unclassified(100);unclassified(100); | RI(3t)upUni | 1 |
| Otu126 | Bacteria(100);Firmicutes(100);Bacilli(100);Bacillales(100);unclassified(100);unclassified(100); | RII(1t)downUni | 19 |
| Otu126 | Bacteria(100);Firmicutes(100);Bacilli(100);Bacillales(100);unclassified(100);unclassified(100); | RII(1t)upUni | 13 |
| Otu126 | Bacteria(100);Firmicutes(100);Bacilli(100);Bacillales(100);unclassified(100);unclassified(100); | RII(2t)downUni | 2 |
| Otu126 | Bacteria(100);Firmicutes(100);Bacilli(100);Bacillales(100);unclassified(100);unclassified(100); | RII(2t)upUni | 11 |
| Otu126 | Bacteria(100);Firmicutes(100);Bacilli(100);Bacillales(100);unclassified(100);unclassified(100); | RII(3t)downUni | 8 |
| Otu126 | Bacteria(100);Firmicutes(100);Bacilli(100);Bacillales(100);unclassified(100);unclassified(100); | RII(3t)upUni | 7 |
| Otu126 | Bacteria(100);Firmicutes(100);Bacilli(100);Bacillales(100);unclassified(100);unclassified(100); | S1Uni | 108 |
| Otu126 | Bacteria(100);Firmicutes(100);Bacilli(100);Bacillales(100);unclassified(100);unclassified(100); | S2Uni | 137 |
| Otu134 | Bacteria(100);"Proteobacteria"(100);Alphaproteobacteria(100);Caulobacterales(100);Caulobacteraceae(100);Brevundimonas(100); | RI(1t)downUni | 44 |
| Otu134 | Bacteria(100);"Proteobacteria"(100);Alphaproteobacteria(100);Caulobacterales(100);Caulobacteraceae(100);Brevundimonas(100); | RI(1t)upUni | 18 |
| Otu134 | Bacteria(100);"Proteobacteria"(100);Alphaproteobacteria(100);Caulobacterales(100);Caulobacteraceae(100);Brevundimonas(100); | RI(2t)downUni | 42 |
| Otu134 | Bacteria(100);"Proteobacteria"(100);Alphaproteobacteria(100);Caulobacterales(100);Caulobacteraceae(100);Brevundimonas(100); | RI(2t)upUni | 573 |
| Otu134 | Bacteria(100);"Proteobacteria"(100);Alphaproteobacteria(100);Caulobacterales(100);Caulobacteraceae(100);Brevundimonas(100); | RI(3t)downUni | 19 |
| Otu134 | Bacteria(100);"Proteobacteria"(100);Alphaproteobacteria(100);Caulobacterales(100);Caulobacteraceae(100);Brevundimonas(100); | RI(3t)upUni | 13 |
| Otu134 | Bacteria(100);"Proteobacteria"(100);Alphaproteobacteria(100);Caulobacterales(100);Caulobacteraceae(100);Brevundimonas(100); | RII(1t)downUni | 78 |
| Otu134 | Bacteria(100);"Proteobacteria"(100);Alphaproteobacteria(100);Caulobacterales(100);Caulobacteraceae(100);Brevundimonas(100); | RII(1t)upUni | 36 |
| Otu134 | Bacteria(100);"Proteobacteria"(100);Alphaproteobacteria(100);Caulobacterales(100);Caulobacteraceae(100);Brevundimonas(100); | RII(2t)downUni | 76 |
| Otu134 | Bacteria(100);"Proteobacteria"(100);Alphaproteobacteria(100);Caulobacterales(100);Caulobacteraceae(100);Brevundimonas(100); | RII(2t)upUni | 309 |
| Otu134 | Bacteria(100);"Proteobacteria"(100);Alphaproteobacteria(100);Caulobacterales(100);Caulobacteraceae(100);Brevundimonas(100); | RII(3t)downUni | 51 |
| Otu134 | Bacteria(100);"Proteobacteria"(100);Alphaproteobacteria(100);Caulobacterales(100);Caulobacteraceae(100);Brevundimonas(100); | RII(3t)upUni | 35 |
| Otu134 | Bacteria(100);"Proteobacteria"(100);Alphaproteobacteria(100);Caulobacterales(100);Caulobacteraceae(100);Brevundimonas(100); | S1Uni | 127 |
| Otu134 | Bacteria(100);"Proteobacteria"(100);Alphaproteobacteria(100);Caulobacterales(100);Caulobacteraceae(100);Brevundimonas(100); | S2Uni | 100 |
| Otu135 | Bacteria(100);"Verrucomicrobia"(100);unclassified(100);unclassified(100);unclassified(100);unclassified(100); | RI(1t)downUni | 14 |
| Otu135 | Bacteria(100);"Verrucomicrobia"(100);unclassified(100);unclassified(100);unclassified(100);unclassified(100); | RI(1t)upUni | 14 |
| Otu135 | Bacteria(100);"Verrucomicrobia"(100);unclassified(100);unclassified(100);unclassified(100);unclassified(100); | RI(2t)downUni | 6 |
| Otu135 | Bacteria(100);"Verrucomicrobia"(100);unclassified(100);unclassified(100);unclassified(100);unclassified(100); | RI(2t)upUni | 17 |
| Otu135 | Bacteria(100);"Verrucomicrobia"(100);unclassified(100);unclassified(100);unclassified(100);unclassified(100); | RI(3t)downUni | 42 |
| Otu135 | Bacteria(100);"Verrucomicrobia"(100);unclassified(100);unclassified(100);unclassified(100);unclassified(100); | RI(3t)upUni | 40 |
| Otu135 | Bacteria(100);"Verrucomicrobia"(100);unclassified(100);unclassified(100);unclassified(100);unclassified(100); | RII(1t)downUni | 29 |
| Otu135 | Bacteria(100);"Verrucomicrobia"(100);unclassified(100);unclassified(100);unclassified(100);unclassified(100); | RII(1t)upUni | 16 |
| Otu135 | Bacteria(100);"Verrucomicrobia"(100);unclassified(100);unclassified(100);unclassified(100);unclassified(100); | RII(2t)downUni | 6 |
| Otu135 | Bacteria(100);"Verrucomicrobia"(100);unclassified(100);unclassified(100);unclassified(100);unclassified(100); | RII(2t)upUni | 43 |
| Otu135 | Bacteria(100);"Verrucomicrobia"(100);unclassified(100);unclassified(100);unclassified(100);unclassified(100); | RII(3t)downUni | 39 |
| Otu135 | Bacteria(100);"Verrucomicrobia"(100);unclassified(100);unclassified(100);unclassified(100);unclassified(100); | RII(3t)upUni | 70 |
| Otu135 | Bacteria(100);"Verrucomicrobia"(100);unclassified(100);unclassified(100);unclassified(100);unclassified(100); | S1Uni | 31 |
| Otu135 | Bacteria(100);"Verrucomicrobia"(100);unclassified(100);unclassified(100);unclassified(100);unclassified(100); | S2Uni | 19 |
| Otu139 | Bacteria(100);Firmicutes(100);Clostridia(100);Clostridiales(100);Peptococcaceae_2(100);Pelotomaculum(100); | RI(1t)downUni | 29 |
| Otu139 | Bacteria(100);Firmicutes(100);Clostridia(100);Clostridiales(100);Peptococcaceae_2(100);Pelotomaculum(100); | RI(1t)upUni | 21 |
| Otu139 | Bacteria(100);Firmicutes(100);Clostridia(100);Clostridiales(100);Peptococcaceae_2(100);Pelotomaculum(100); | RI(2t)downUni | 1 |
| Otu139 | Bacteria(100);Firmicutes(100);Clostridia(100);Clostridiales(100);Peptococcaceae_2(100);Pelotomaculum(100); | RI(2t)upUni | 36 |
| Otu139 | Bacteria(100);Firmicutes(100);Clostridia(100);Clostridiales(100);Peptococcaceae_2(100);Pelotomaculum(100); | RI(3t)downUni | 6 |
| Otu139 | Bacteria(100);Firmicutes(100);Clostridia(100);Clostridiales(100);Peptococcaceae_2(100);Pelotomaculum(100); | RI(3t)upUni | 11 |
| Otu139 | Bacteria(100);Firmicutes(100);Clostridia(100);Clostridiales(100);Peptococcaceae_2(100);Pelotomaculum(100); | RII(1t)downUni | 81 |
| Otu139 | Bacteria(100);Firmicutes(100);Clostridia(100);Clostridiales(100);Peptococcaceae_2(100);Pelotomaculum(100); | RII(1t)upUni | 29 |
| Otu139 | Bacteria(100);Firmicutes(100);Clostridia(100);Clostridiales(100);Peptococcaceae_2(100);Pelotomaculum(100); | RII(2t)downUni | 8 |
| Otu139 | Bacteria(100);Firmicutes(100);Clostridia(100);Clostridiales(100);Peptococcaceae_2(100);Pelotomaculum(100); | RII(2t)upUni | 15 |
| Otu139 | Bacteria(100);Firmicutes(100);Clostridia(100);Clostridiales(100);Peptococcaceae_2(100);Pelotomaculum(100); | RII(3t)downUni | 31 |
| Otu139 | Bacteria(100);Firmicutes(100);Clostridia(100);Clostridiales(100);Peptococcaceae_2(100);Pelotomaculum(100); | RII(3t)upUni | 12 |
| Otu139 | Bacteria(100);Firmicutes(100);Clostridia(100);Clostridiales(100);Peptococcaceae_2(100);Pelotomaculum(100); | S1Uni | 170 |
| Otu139 | Bacteria(100);Firmicutes(100);Clostridia(100);Clostridiales(100);Peptococcaceae_2(100);Pelotomaculum(100); | S2Uni | 222 |
| Otu159 | Bacteria(100);"Spirochaetes"(100);Spirochaetes(100);Spirochaetales(100);Spirochaetaceae(100);Spirochaeta(100); | RI(1t)downUni | 40 |
| Otu159 | Bacteria(100);"Spirochaetes"(100);Spirochaetes(100);Spirochaetales(100);Spirochaetaceae(100);Spirochaeta(100); | RI(1t)upUni | 5 |
| Otu159 | Bacteria(100);"Spirochaetes"(100);Spirochaetes(100);Spirochaetales(100);Spirochaetaceae(100);Spirochaeta(100); | RI(2t)downUni | 8 |
| Otu159 | Bacteria(100);"Spirochaetes"(100);Spirochaetes(100);Spirochaetales(100);Spirochaetaceae(100);Spirochaeta(100); | RI(2t)upUni | 90 |
| Otu159 | Bacteria(100);"Spirochaetes"(100);Spirochaetes(100);Spirochaetales(100);Spirochaetaceae(100);Spirochaeta(100); | RI(3t)downUni | 31 |
| Otu159 | Bacteria(100);"Spirochaetes"(100);Spirochaetes(100);Spirochaetales(100);Spirochaetaceae(100);Spirochaeta(100); | RI(3t)upUni | 3 |
| Otu159 | Bacteria(100);"Spirochaetes"(100);Spirochaetes(100);Spirochaetales(100);Spirochaetaceae(100);Spirochaeta(100); | RII(1t)downUni | 44 |
| Otu159 | Bacteria(100);"Spirochaetes"(100);Spirochaetes(100);Spirochaetales(100);Spirochaetaceae(100);Spirochaeta(100); | RII(1t)upUni | 29 |
| Otu159 | Bacteria(100);"Spirochaetes"(100);Spirochaetes(100);Spirochaetales(100);Spirochaetaceae(100);Spirochaeta(100); | RII(2t)downUni | 8 |
| Otu159 | Bacteria(100);"Spirochaetes"(100);Spirochaetes(100);Spirochaetales(100);Spirochaetaceae(100);Spirochaeta(100); | RII(2t)upUni | 75 |
| Otu159 | Bacteria(100);"Spirochaetes"(100);Spirochaetes(100);Spirochaetales(100);Spirochaetaceae(100);Spirochaeta(100); | RII(3t)downUni | 45 |
| Otu159 | Bacteria(100);"Spirochaetes"(100);Spirochaetes(100);Spirochaetales(100);Spirochaetaceae(100);Spirochaeta(100); | RII(3t)upUni | 71 |
| Otu159 | Bacteria(100);"Spirochaetes"(100);Spirochaetes(100);Spirochaetales(100);Spirochaetaceae(100);Spirochaeta(100); | S1Uni | 100 |
| Otu159 | Bacteria(100);"Spirochaetes"(100);Spirochaetes(100);Spirochaetales(100);Spirochaetaceae(100);Spirochaeta(100); | S2Uni | 208 |
| Otu176 | Bacteria(100);"Actinobacteria"(100);Actinobacteria(100);Actinomycetales(100);Dietziaceae(100);Dietzia(100); | RI(1t)downUni | 2 |
| Otu176 | Bacteria(100);"Actinobacteria"(100);Actinobacteria(100);Actinomycetales(100);Dietziaceae(100);Dietzia(100); | RI(1t)upUni | 19 |
| Otu176 | Bacteria(100);"Actinobacteria"(100);Actinobacteria(100);Actinomycetales(100);Dietziaceae(100);Dietzia(100); | RI(2t)downUni | 3 |
| Otu176 | Bacteria(100);"Actinobacteria"(100);Actinobacteria(100);Actinomycetales(100);Dietziaceae(100);Dietzia(100); | RI(2t)upUni | 29 |
| Otu176 | Bacteria(100);"Actinobacteria"(100);Actinobacteria(100);Actinomycetales(100);Dietziaceae(100);Dietzia(100); | RI(3t)downUni | 4 |
| Otu176 | Bacteria(100);"Actinobacteria"(100);Actinobacteria(100);Actinomycetales(100);Dietziaceae(100);Dietzia(100); | RI(3t)upUni | 5 |
| Otu176 | Bacteria(100);"Actinobacteria"(100);Actinobacteria(100);Actinomycetales(100);Dietziaceae(100);Dietzia(100); | RII(1t)downUni | 13 |
| Otu176 | Bacteria(100);"Actinobacteria"(100);Actinobacteria(100);Actinomycetales(100);Dietziaceae(100);Dietzia(100); | RII(1t)upUni | 16 |
| Otu176 | Bacteria(100);"Actinobacteria"(100);Actinobacteria(100);Actinomycetales(100);Dietziaceae(100);Dietzia(100); | RII(2t)downUni | 6 |
| Otu176 | Bacteria(100);"Actinobacteria"(100);Actinobacteria(100);Actinomycetales(100);Dietziaceae(100);Dietzia(100); | RII(2t)upUni | 14 |
| Otu176 | Bacteria(100);"Actinobacteria"(100);Actinobacteria(100);Actinomycetales(100);Dietziaceae(100);Dietzia(100); | RII(3t)downUni | 13 |
| Otu176 | Bacteria(100);"Actinobacteria"(100);Actinobacteria(100);Actinomycetales(100);Dietziaceae(100);Dietzia(100); | RII(3t)upUni | 28 |
| Otu176 | Bacteria(100);"Actinobacteria"(100);Actinobacteria(100);Actinomycetales(100);Dietziaceae(100);Dietzia(100); | S1Uni | 4 |
| Otu176 | Bacteria(100);"Actinobacteria"(100);Actinobacteria(100);Actinomycetales(100);Dietziaceae(100);Dietzia(100); | S2Uni | 4 |
| Otu207 | Bacteria(100);"Proteobacteria"(100);Alphaproteobacteria(100);Rhizobiales(100);Hyphomicrobiaceae(100);Devosia(100); | RI(1t)downUni | 1 |
| Otu207 | Bacteria(100);"Proteobacteria"(100);Alphaproteobacteria(100);Rhizobiales(100);Hyphomicrobiaceae(100);Devosia(100); | RI(1t)upUni | 3 |
| Otu207 | Bacteria(100);"Proteobacteria"(100);Alphaproteobacteria(100);Rhizobiales(100);Hyphomicrobiaceae(100);Devosia(100); | RI(2t)downUni | 12 |
| Otu207 | Bacteria(100);"Proteobacteria"(100);Alphaproteobacteria(100);Rhizobiales(100);Hyphomicrobiaceae(100);Devosia(100); | RI(2t)upUni | 95 |
| Otu207 | Bacteria(100);"Proteobacteria"(100);Alphaproteobacteria(100);Rhizobiales(100);Hyphomicrobiaceae(100);Devosia(100); | RI(3t)downUni | 1 |
| Otu207 | Bacteria(100);"Proteobacteria"(100);Alphaproteobacteria(100);Rhizobiales(100);Hyphomicrobiaceae(100);Devosia(100); | RI(3t)upUni | 2 |
| Otu207 | Bacteria(100);"Proteobacteria"(100);Alphaproteobacteria(100);Rhizobiales(100);Hyphomicrobiaceae(100);Devosia(100); | RII(1t)downUni | 3 |
| Otu207 | Bacteria(100);"Proteobacteria"(100);Alphaproteobacteria(100);Rhizobiales(100);Hyphomicrobiaceae(100);Devosia(100); | RII(1t)upUni | 2 |
| Otu207 | Bacteria(100);"Proteobacteria"(100);Alphaproteobacteria(100);Rhizobiales(100);Hyphomicrobiaceae(100);Devosia(100); | RII(2t)downUni | 12 |
| Otu207 | Bacteria(100);"Proteobacteria"(100);Alphaproteobacteria(100);Rhizobiales(100);Hyphomicrobiaceae(100);Devosia(100); | RII(2t)upUni | 42 |
| Otu207 | Bacteria(100);"Proteobacteria"(100);Alphaproteobacteria(100);Rhizobiales(100);Hyphomicrobiaceae(100);Devosia(100); | RII(3t)downUni | 29 |
| Otu207 | Bacteria(100);"Proteobacteria"(100);Alphaproteobacteria(100);Rhizobiales(100);Hyphomicrobiaceae(100);Devosia(100); | RII(3t)upUni | 7 |
| Otu207 | Bacteria(100);"Proteobacteria"(100);Alphaproteobacteria(100);Rhizobiales(100);Hyphomicrobiaceae(100);Devosia(100); | S1Uni | 50 |
| Otu207 | Bacteria(100);"Proteobacteria"(100);Alphaproteobacteria(100);Rhizobiales(100);Hyphomicrobiaceae(100);Devosia(100); | S2Uni | 64 |
| Otu215 | Bacteria(100);"Actinobacteria"(100);Actinobacteria(100);Actinomycetales(100);Microbacteriaceae(100);Leucobacter(100); | RI(1t)downUni | 2 |
| Otu215 | Bacteria(100);"Actinobacteria"(100);Actinobacteria(100);Actinomycetales(100);Microbacteriaceae(100);Leucobacter(100); | RI(1t)upUni | 1 |
| Otu215 | Bacteria(100);"Actinobacteria"(100);Actinobacteria(100);Actinomycetales(100);Microbacteriaceae(100);Leucobacter(100); | RI(2t)downUni | 4 |
| Otu215 | Bacteria(100);"Actinobacteria"(100);Actinobacteria(100);Actinomycetales(100);Microbacteriaceae(100);Leucobacter(100); | RI(2t)upUni | 11 |
| Otu215 | Bacteria(100);"Actinobacteria"(100);Actinobacteria(100);Actinomycetales(100);Microbacteriaceae(100);Leucobacter(100); | RI(3t)downUni | 2 |
| Otu215 | Bacteria(100);"Actinobacteria"(100);Actinobacteria(100);Actinomycetales(100);Microbacteriaceae(100);Leucobacter(100); | RI(3t)upUni | 2 |
| Otu215 | Bacteria(100);"Actinobacteria"(100);Actinobacteria(100);Actinomycetales(100);Microbacteriaceae(100);Leucobacter(100); | RII(1t)downUni | 1 |
| Otu215 | Bacteria(100);"Actinobacteria"(100);Actinobacteria(100);Actinomycetales(100);Microbacteriaceae(100);Leucobacter(100); | RII(1t)upUni | 2 |
| Otu215 | Bacteria(100);"Actinobacteria"(100);Actinobacteria(100);Actinomycetales(100);Microbacteriaceae(100);Leucobacter(100); | RII(2t)downUni | 2 |
| Otu215 | Bacteria(100);"Actinobacteria"(100);Actinobacteria(100);Actinomycetales(100);Microbacteriaceae(100);Leucobacter(100); | RII(2t)upUni | 4 |
| Otu215 | Bacteria(100);"Actinobacteria"(100);Actinobacteria(100);Actinomycetales(100);Microbacteriaceae(100);Leucobacter(100); | RII(3t)downUni | 9 |
| Otu215 | Bacteria(100);"Actinobacteria"(100);Actinobacteria(100);Actinomycetales(100);Microbacteriaceae(100);Leucobacter(100); | RII(3t)upUni | 12 |
| Otu215 | Bacteria(100);"Actinobacteria"(100);Actinobacteria(100);Actinomycetales(100);Microbacteriaceae(100);Leucobacter(100); | S1Uni | 54 |
| Otu215 | Bacteria(100);"Actinobacteria"(100);Actinobacteria(100);Actinomycetales(100);Microbacteriaceae(100);Leucobacter(100); | S2Uni | 43 |
