# Supplementary material for: Qualitative Analysis of Microbial Dynamics during Anaerobic Digestion of Microalgal Biomass in a UASB Reactor
Source: Int J Microbiol. 2017 Nov 13;2017:5291283. doi: 10.1155/2017/5291283 (PMC5702946; doi:10.1155/2017/5291283)
Supplement: Supplementary file 1 — Supplementary Table 1: Primers used in the reported study. Supplementary Table 2: Core set of OTUs, shared among all sampling time points during the anaerobic digestion of microalgal biomass and sodium acetate in duplicates of UASB reactors. Supplemental Figure 1: Schematics of the Upflow Anaerobic Sludge Blanket reactor (UASB) used in the study. Supplemental Figure 2: Heatmap, calculated with jclass algorithm in MOTHUR, representing beta-diversity (internal compositional heterogeneity) of samples taken at the same time point from two reactors. Labels “Uni” represent 16S rRNA universal primer set used in the study. Red-colored scale from 0.0 to 1.0 should be interpreted as the 1.0 bright color correspond to the closely related samples. Opposite is true for the 0.0 marking and dark red color. Supplementary Figure 3: A. General workflow anaerobic digestion of microalgal biomass and analysis of eubacterial and methanogenic communities. B. Workflow for the sequence analysis and identification of microorganisms (via MOTHUR MiSeq_SOP). [file 5291283.f1.zip › Supplementary materials/Supplemetal Figure 1_IJMICRO_2043576.docx]

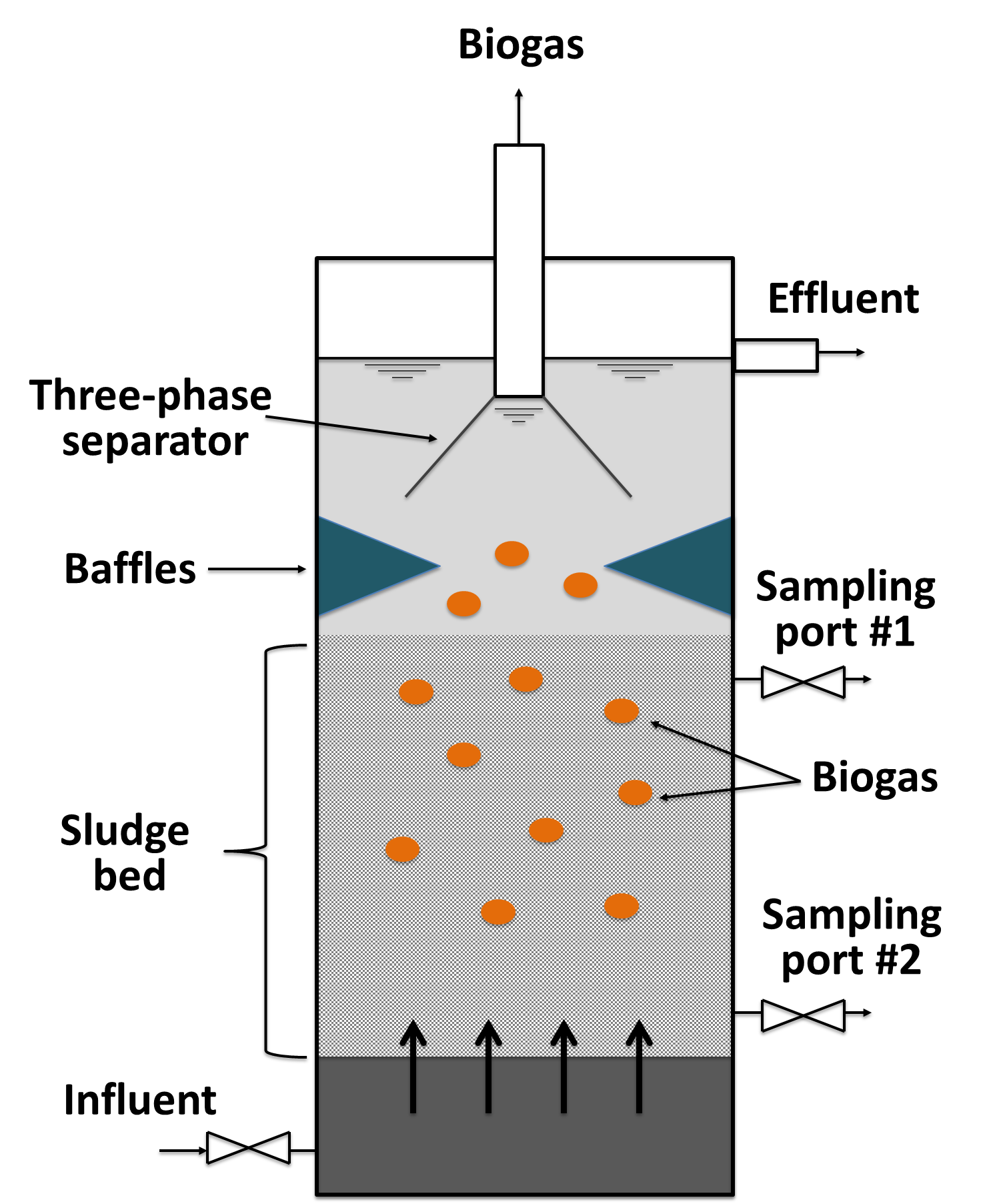


Supplemental Figure 1. Schematics of the Upflow Anaerobic Sludge Blanket reactor (UASB) used in the study.
